# Biomarkers to predict relapse in myelin oligodendrocyte glycoprotein antibody-associated disease: a systematic review and meta-analysis

**DOI:** 10.1136/jnnp-2025-337039

**Published:** 2025-10-01

**Authors:** Jane Andersen, Benjamin Peter Trewin, Russell C Dale, Sudarshini Ramanathan, Fabienne Brilot

**Affiliations:** 1 Kids Neuroscience Centre, Kids Research at the Children’s Hospital at Westmead, Sydney, NSW, Australia; 2 Faculty of Medicine and Health, Sydney Medical School, The University of Sydney, Sydney, NSW, Australia; 3 Brain and Mind Centre, The University of Sydney, Sydney, NSW, Australia; 4 Department of Neurology, Concord Hospital, Concord, NSW, Australia; 5 Faculty of Medicine and Health, School of Medical Sciences, The University of Sydney, Sydney, NSW, Australia

## Abstract

**Background:**

Detection of immunoglobulin G targeting myelin oligodendrocyte glycoprotein (MOG-IgG) is the mainstay of laboratory diagnosis of MOG antibody-associated disease. Laboratory biomarkers have the potential to predict disease course and activity, thus informing prompt therapeutic decisions to minimise relapse-associated disability accrual.

**Methods:**

This systematic review with meta-analysis was registered in PROSPERO (CRD42024554429). MEDLINE, Embase and Scopus databases were searched. Random-effects or mixed-effects modelling was performed and OR or HR with 95% CIs reported.

**Results:**

106 studies with ≥1710 individuals were included. A relapsing course was associated with persistent seropositivity on serial samples collected ≥3 months apart (OR 2.7 (95% CI 1.8 to 4.0), p<0.0001), lower likelihood of seroreversion to negative status (HR 0.19 (95% CI 0.14 to 0.26), p<0.0001) and delayed seroreversion compared with monophasic participants (median 19 years vs 2.5 years, p<0.0001). Acute disseminated encephalomyelitis was associated with a non-relapsing course (OR 0.049 (95% CI 0.0029 to 0.84), p=0.037). Serum MOG-IgG titre—negative, low positive or clear positive—discriminated disease state: attack was associated with clear positive titre (OR 3.6 (95% CI 2.6 to 5.0), p<0.0001), but not negative titre (OR 0.073 (95% CI 0.028 to 0.19), p<0.0001). Cerebrospinal fluid (CSF) leucocytosis (≥5 cells/µL) was associated with attack (OR 3.1 (95% CI 1.7 to 5.9), p=0.0004). Neither serum glial fibrillary acidic protein nor neurofilament light chain correlated with disease activity. Novel biomarkers of disease course and activity have also been assessed qualitatively.

**Conclusions:**

MOG-IgG serostatus and titre and CSF leucocytosis are biomarkers of disease course and activity. The findings provide rationale for serial serum MOG-IgG testing at an interval of 3–6 months in the first 12 months of disease to assist in relapse risk stratification.

**PROSPERO registration number:**

CRD42024554429.

**WHAT IS ALREADY KNOWN ON THIS TOPIC**

- Myelin oligodendrocyte glycoprotein (MOG) immunoglobulin G (IgG) seropositivity is a prerequisite in the diagnosis of MOG antibody-associated disease (MOGAD). It is unclear whether laboratory biomarkers may be used to predict disease course or activity.

**WHAT THIS STUDY ADDS**

- This systematic review with meta-analysis addresses the common constraint in MOGAD research of limited sample sizes due to the rarity of its incidence. Relapsing disease course is shown to be associated with persistent seropositivity on serial serum MOG-IgG measurement, reduced likelihood of seroreverting to MOG-IgG negative status and delayed seroreversion compared with monophasic individuals; additionally, both serum MOG-IgG titre and cerebrospinal fluid (CSF) white cell count (WCC) effectively discriminate attack from remission state.

**HOW THIS STUDY MIGHT AFFECT RESEARCH, PRACTICE OR POLICY**

- Clinicians may consider serial measurement of serum MOG-IgG at intervals of 3–6 months in the 12 months following disease onset to stratify risk of relapsing disease course. Measurement of serum MOG-IgG titre and CSF WCC may be diagnostically useful in settings of clinical uncertainty whereby an individual, who has an existing diagnosis of MOGAD, has symptoms that are not clearly attributable to a MOGAD relapse.

## Introduction

 Myelin oligodendrocyte glycoprotein antibody-associated disease (MOGAD) is an inflammatory demyelinating disease of the central nervous system (CNS), characterised by the presence of anti-MOG autoantibodies (MOG-IgG).^1^ MOGAD is typically associated with the clinical phenotypes acute disseminated encephalomyelitis (ADEM), optic neuritis (ON) or transverse myelitis (TM) and is less commonly associated with cerebral cortical encephalitis, brainstem presentations or cerebellar presentations.^1^ MOGAD affects both children and adults; however, there is a distinct divergence of phenotype—ADEM is the most frequent paediatric phenotype compared with ON as the primary manifestation in adults.^2^ The disease course is either monophasic or relapsing, with a recent review reporting that relapsing disease course occurs in 72% of individuals when follow-up is extended beyond 5 years, suggesting that this is a predominantly relapsing disorder in the context of current diagnostic and therapeutic approaches.^3^ The morbidity burden associated with MOGAD is significant, with residual neurological disability manifesting as visual acuity loss, motor deficits, sensory deficits, cognitive impairment and sphincter dysfunction.^4^,^6^ Moreover, disability may accumulate with relapses.^4^ Thus, disease course prediction is a paramount yet elusive goal, which has the potential to facilitate early institution of maintenance immunotherapy to limit the accrual of disability that occurs with relapse while simultaneously avoiding unnecessary immunosuppression in monophasic individuals. Laboratory biomarkers offer an attractive avenue to address this conundrum. To date, temporal dynamics of serum MOG-IgG have been the most thoroughly investigated potential biomarker, with some studies reporting a significant association between persistent seropositive measurements and relapsing disease course^7^,^9^; however, the literature has lacked consensus regarding this relationship.^6 10^ Additionally, biomarkers of disease activity, rather than of disease course, may be diagnostically useful in settings of clinical uncertainty to discriminate whether symptoms represent a MOGAD attack as opposed to an alternate pathology. A widespread challenge of MOGAD research has been the rarity of its incidence—estimated at 1.6–3.4 per 1 000 000 person-years—which often translates to limited sample sizes.^11 12^ For these reasons, we aimed to conduct a systematic review with meta-analysis to investigate the capability of laboratory biomarkers to predict disease course, as well as differentiate remission compared with attack disease activity.

## Methods

### Search strategy

**Figure 1:**
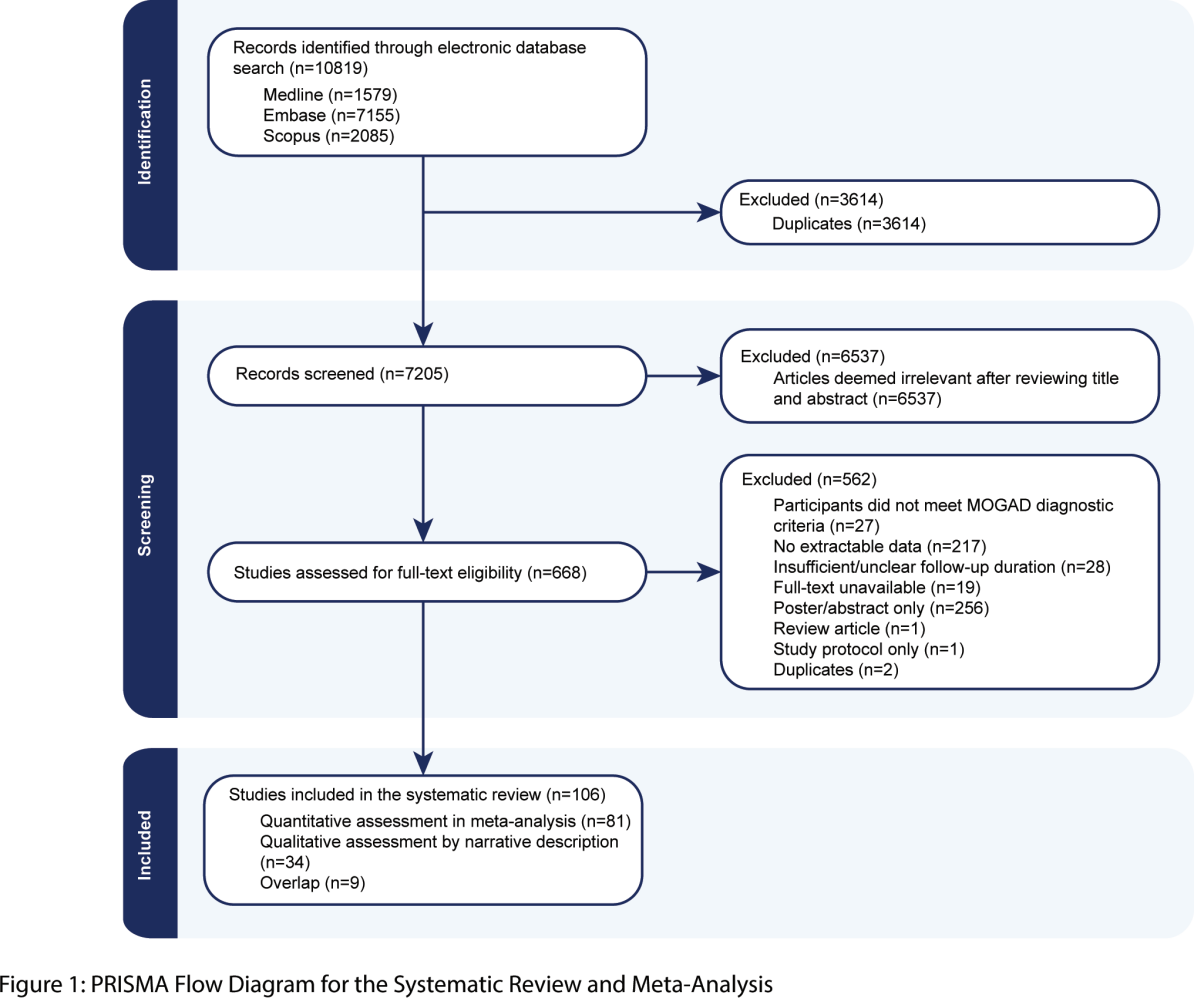
Preferred Reporting Items for Systematic Reviews and Meta-Analyses flow diagram. MOGAD, myelin oligodendrocyte glycoprotein antibody-associated disease.

This systematic review with meta-analysis was conducted in accordance with the Preferred Reporting Items for Systematic Reviews and Meta-Analyses (PRISMA) 2020 statement and using the Covidence platform.^13^ The PRISMA flowchart is presented (figure 1). The study was registered with PROSPERO (registration number CRD42024554429). A systematic search of the databases MEDLINE, Embase and Scopus was performed of all studies from inception to 21 February 2024, with English language restriction. The complete search strategy is presented in online supplemental table 1. The literature search was performed by one author (JA) and reviewed by the senior author (FB).

### Key definitions

Key definitions are presented in online supplemental tables 2 and 3.

### Study selection

Study eligibility was assessed based on the following inclusion criteria: (1) published, full-text original research articles in English, (2) measured laboratory biomarker(s) in terms of disease course and/or disease activity, (3) participants met the 2023 International MOGAD Panel proposed diagnostic criteria, broadly defined by positive serum MOG-IgG measurement on cell-based assay (CBA) and characteristic clinical phenotype as per definitions outlined in online supplemental tables 2 and 3, (4) studies that measured disease course clearly reported follow-up duration as ≥1 year either in a direct statement or able to be inferred from descriptive statistics such as minimum-maximum range (reporting of only IQR or SD was insufficient) and (5) extractable data for ≥2 individuals, with data only being extracted for individuals meeting the inclusion criteria. There were no restrictions on follow-up duration for studies assessing disease activity. There were no restrictions on the types of study design eligible for inclusion. There were no restrictions based on individuals’ age, sex or comorbidity. All disagreements were resolved by consensus.

### Data extraction, study risk of bias assessment and certainty of evidence assessment

One author (JA) independently extracted data from all included studies with a standardised data extraction form hosted on Microsoft Excel software (V.16.97.2). Data were sought for the following outcomes of interest: (1) laboratory biomarker(s) sampled at onset or any time during disease that were associated with disease course and (2) laboratory biomarker(s) that were associated with disease activity. Data were extracted as reported in the text, tables and figures of the original papers and their published supplementary/appendix material. In the event of missing or incomplete data, the data or study (if there was no other extractable data for inclusion) were excluded; study investigators were not contacted. One author (JA) independently assessed the risk of bias of included studies using the Newcastle-Ottawa Quality Assessment Scale. Risk of bias scores were categorised into ‘low’ (7–9), ‘medium’ (4–6) and ‘high’ (0–3). One author (JA) assessed outcomes in terms of association, of any magnitude, with disease course and/or disease activity using the Grading of Recommendations Assessment, Development and Evaluation (GRADE)approach.^14^ The certainty of the evidence was rated as ‘high’, ‘moderate’, ‘low’ or ‘very low’. This study assessed association rather than causation questions; thus, the most appropriate study design to answer these questions was observational studies.^14^ Accordingly, the assessment of certainty started at ‘high’ and was downgraded one level for serious (or by two levels for very serious) limitations in the study design, inconsistency, imprecision, indirectness and publication bias.^14^ The senior author (FB) reviewed the extracted data and risk of bias and certainty of evidence assessments. All disagreements were resolved by consensus.

### Statistical analysis

All analyses were performed using R software (V.4.2.2) with the metafor, forestplot, lme4, coxme, dplyr and estmeansd packages. Individual participant data (IPD) were extracted where available, otherwise aggregate data (AD) were extracted. Missing or incomplete data were excluded. For studies that reported median (range or IQR) summary statistics, the method for unknown non-normal distributions approach was used to estimate mean±SD Biomarker(s) were analysed quantitatively if there were extractable data from ≥3 studies, otherwise analysis was qualitative with tabulated and in-text description. A minimum subgroup sample size criterion (n≥5) was applied to optimise model performance. Effect measures were: (1) ordinal outcome data were described with ORs with 95% CIs, with univariable analyses using a two-stage approach, where IPD was reduced to study-level effect estimates then combined with random-effects meta-analysis and multivariable analyses using a one-stage IPD meta-analysis with logistic mixed-effects regression model, to account for clustering of participants within studies, (2) time-to-event outcome data were described with HRs with 95% CI, with univariable and multivariable analyses using a one-stage IPD meta-analysis with mixed-effects Cox regression model, to account for clustering of participants within studies, and using Kaplan-Meier survival curve for visualisation and (3) continuous outcome data were described with mean differences with 95% CI, where AD were combined in a random-effects meta-analysis of mean differences. The measurement scales and collection methodologies of continuous outcome data did not differ across studies; thus, standardisation was not required. All random-effects meta-analyses employed restricted maximum likelihood estimation, apart from one model which failed to converge and, thus, employed DerSimonian-Laird method.

In univariable random-effects meta-analyses, the degree of variability or inconsistency across different studies, termed ‘heterogeneity’, was assessed using the I^2^ statistic.^15^ For mixed-effects meta-analyses, an R statistic was estimated, which compared the impact of heterogeneity in the fixed-effect and random-effects models as previously described.^15^ The R statistic was used to calculate an estimated I^2^ statistic with the equation: I^2^ = (R^2^ – 1)/R^2^.^16^ The following interpretation for I^2^ was applied^17 18^; 0–40%: might not be important; 30–60%: may represent moderate between-study heterogeneity; 50–90%: may represent substantial heterogeneity; 75–100%: considerable heterogeneity.

Potential publication bias was evaluated using the Egger regression test, with p<0.05 defined as statistically significant and funnel plots for visualisation. Subgroup analyses were conducted to explore potential sources of heterogeneity after it was identified.

## Results

The database search identified 10 819 articles. Following exclusion of duplicates, 7205 articles were screened by title and abstract, of which 668 were deemed relevant and screened by full text. 106 articles, all observational studies, met the inclusion criteria and were included in the systematic review. 81 studies had extractable data sets able to be assessed quantitatively in the meta-analysis (online supplemental table 4). Of these, 77 studies contributed 1710 participants. Four studies specified sample numbers for part or all of their dataset and so the exact number of participants could not be inferred. 34 included studies were assessed in a qualitative capacity by narrative description (online supplemental table 4). Nine studies overlapped and were assessed in both a qualitative and quantitative capacity (online supplemental table 4).

Characteristics of included studies are outlined (online supplemental table 4). 72% (76/106) of studies had a low risk of bias, 28% (30/106) had a medium risk and 0 studies had a high risk (online supplemental table 4).

48 studies had extractable data for follow-up duration of both monophasic and relapsing groups, which allowed pairwise comparison at the level of the individual study (online supplemental table 5). 25% (12/48) of studies were found to have statistically significant longer follow-up duration for individuals with a relapsing course compared with monophasic course (online supplemental table 5).

### Biomarkers of disease course

**Figure 2:**
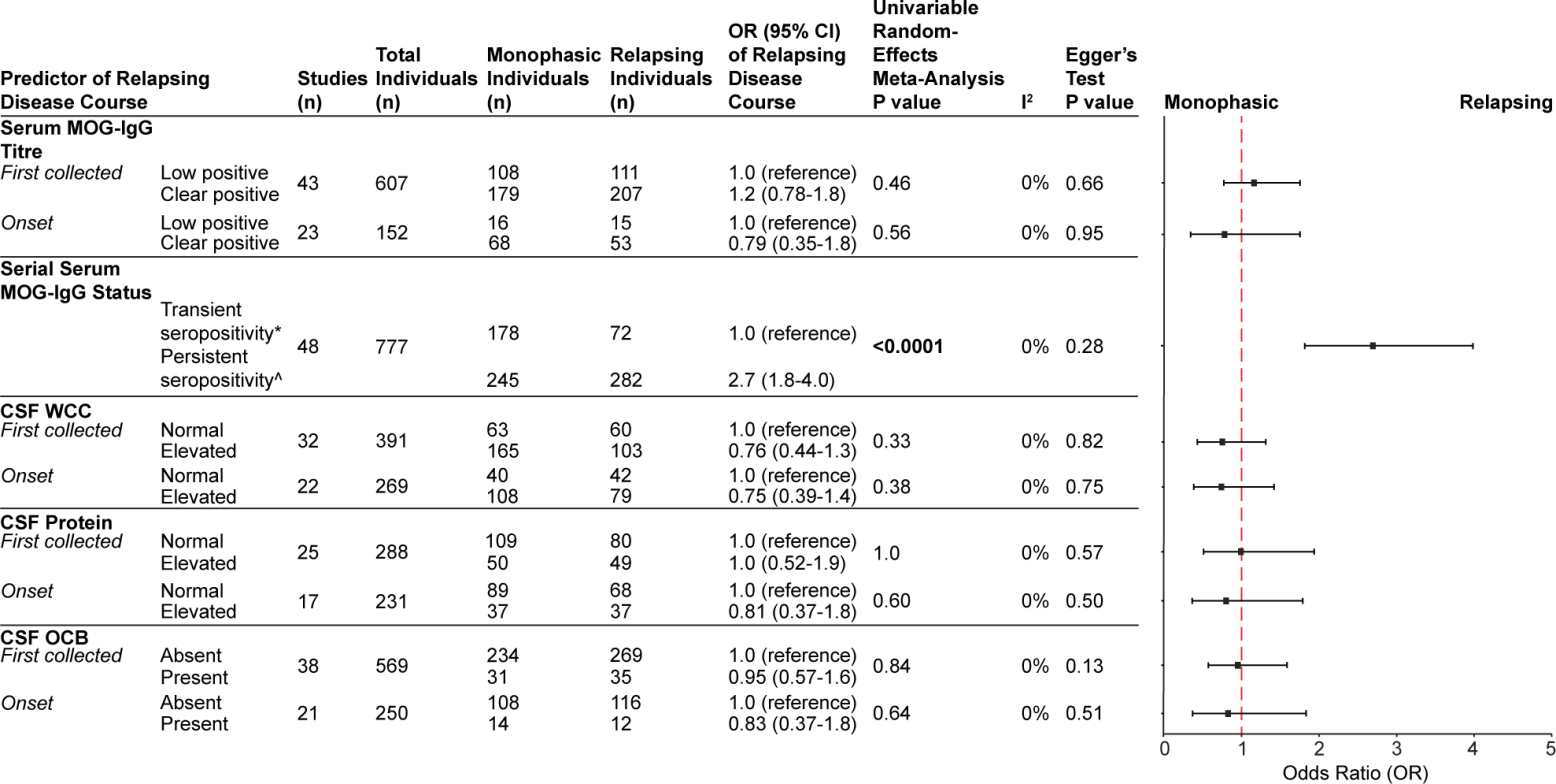
Investigating the association of biomarkers and relapsing disease course. ‘First collected’ analyses included the first collected serum or CSF specimen for an individual including, but not limited to, all samples collected at onset. ‘Onset’ analyses only included serum or CSF specimens collected <30 days following first clinical symptoms of MOGAD. *Transient seropositivity was defined as <2 positive serum MOG-IgG results on serial measurement ≥3 months apart. ˆPersistent seropositivity was defined as ≥2 positive serum MOG-IgG results on serial measurement ≥3 months apart; individuals whose MOG-IgG measurements fluctuated between positive and negative were categorised as persistent seropositive if there were ≥2 positive results, ≥3 months apart. CSF, cerebrospinal fluid; OCB, oligoclonal band; WCC, white cell count.

Biomarkers collected at disease onset (<30 days following first MOGAD attack) or from the first collected biospecimen (including but not limited to those collected strictly at onset) were assessed for capability to predict disease course. The most frequently reported biomarkers for disease course prediction, in descending order, were: (1) serial serum MOG Ig-G status, (2) serum MOG-IgG titre, (3) cerebrospinal fluid (CSF) oligoclonal band (OCB) status, (4) CSF white cell count (WCC) and (5) CSF protein. Importantly, treatment status at time of sample collection was incompletely available and, thus, was not included in any proceeding analyses. Persistent seropositivity on serial serum MOG-IgG measurement collected ≥3 months apart conferred statistically significant increased odds of relapsing course compared with transient seropositivity (OR 2.7 (95% CI 1.8 to 4.0), p<0.0001) (figure 2 and online supplemental figure 1). No significant heterogeneity was detected (I^2^=0%) and Egger’s test did not indicate significant publication bias (p=0.28). Persistent seropositivity remained associated with increased odds of relapsing course compared with transient seropositivity when the sample collection interval was lengthened to ≥6 months apart (OR 2.3 (95% CI 1.5 to 3.4), p<0.0001), as well as ≥12 months apart (OR 2.8 (95% CI 1.8 to 4.5), p<0.0001) (online supplemental table 6). None of the other variables were significantly associated with relapsing course either in the first collected or onset samples (figure 2). No significant heterogeneity was observed (I^2^=0% for all analyses) nor was any significant publication bias detected (p>0.05 for all analyses).

The association between persistent seropositivity and relapsing course was further explored in terms of individual-level covariables, including sex, age and clinical phenotype (table 1). Individuals who had serial serum MOG-IgG samples collected ≥3 months apart were examined for complete demographic data, which was only extractable for 131 individuals from 52% (25/48) of studies that reported serial serum MOG-IgG status. Data for three individuals with ‘other’ phenotype were excluded due to insufficient subgroup sample size. The final analysis included 128 individuals. Persistent seropositivity remained significantly associated with increased odds of relapsing disease course in the multivariable analysis (OR 15, 95% CI 2.2 to 98, p=0.0059) (table 1). There was substantial heterogeneity (I^2^=77%), likely due to limited participant numbers. In addition, ADEM phenotype was found to be significantly associated with reduced risk of relapsing disease course (OR 0.049 (0.0029–0.84, p=0.037) (table 1). Individual follow-up duration was available for 117/128 individuals. No statistically significant difference in follow-up duration between phenotype subgroups was identified with the Kruskal-Wallis test (results not shown). Complete demographic data were available for 110 individuals with serial serum MOG-IgG samples collected ≥6 months apart and for 96 individuals with serial serum MOG-IgG samples collected ≥12 months apart. Interestingly, persistent seropositivity was not significantly associated with relapsing course in either of these multivariable analyses (online supplemental table 7). There is substantial heterogeneity with both analyses (I^2^=94% and I^2^=83%, respectively).

**Table 1:** Investigating the multivariable association of persistent seropositivity and relapsing disease course alongside individual-level demographics

Predictor of relapsing disease course*	Total participants (n)	Monophasic participants (n)	Relapsing participants (n)	OR (95% CI) of relapsing disease course	P value†
Serial MOG-IgG status					
Transient seropositivity‡	32	23	9	1.0 (reference)	0.0059
Persistent seropositivity§	96	18	78	15 (2.2 to 98)	
Sex					
Male	59	25	34	1.0 (reference)	0.93
Female	69	16	53	1.1 (0.27 to 4.2)	
Age					
Paediatric (<18 years)	77	27	50	1.0 (reference)	0.71
Adult (≥18 years)	51	14	37	1.5 (0.17 to 14)	
Phenotype					
Brain/brainstem	23	7	16	1.0 (reference)	—
ADEM	26	18	8	0.049 (0.0029 to 0.84)	0.037
ON	20	8	12	0.99 (0.072 to 14)	1.0
TM	5	3	2	0.20 (0.0059 to 6.4)	0.36
ADEM+ON	16	1	15	5.6 (0.15 to 205)	0.35
ON+TM	10	2	8	6. 7 (0.12 to 360)	0.35
Mixed	28	2	26	11 (0.61 to 193)	0.10

* Minimum subgroup sample size criterion (n ≥5); phenotype ‘other’ was excluded due to insufficient participant numbers (n=3).

† Analysis was with a one-stage IPD meta-analysis with multivariable logistic mixed-effects regression. There was substantial heterogeneity (I2=77%).

‡ Transient seropositivity was defined as <2 positive serum MOG-IgG results on serial measurement ≥3 months apart.

§ Persistent seropositivity was defined as ≥2 positive serum MOG-IgG results on serial measurement ≥3 months apart. Individuals whose MOG-IgG measurements fluctuated between seropositive and seronegative were categorised as persistent seropositive if there were ≥2 positive results ≥3 months apart.

ADEM, acute disseminated encephalomyelitis; IPD, Individual participant data; MOG-IgG, immunoglobulin G targeting myelin oligodendrocyte glycoprotein; ON, optic neuritis; TM, transverse myelitis.

Next, we analysed whether any clinical predictors were associated with seroreversion to MOG-IgG negative status (table 2). In the univariable analysis, relapsing disease course, female sex and ON+TM phenotype all had significantly decreased likelihood of seroreversion to negative status, while ADEM phenotype conferred significantly increased likelihood of seroreversion to negative status (table 2). In the multivariable analysis, only the association between relapsing course and reduced likelihood of seroreversion to negative status remained statistically significant (table 2).

**Table 2:** Investigating the association of clinical predictors and seroreversion to MOG-IgG negative status*

Predictor of seroreversion to MOG-IgG negative status†	Univariable			Multivariable		
	Participants (n) (studies (n))	HR (95% CI) of seroreversion to MOG-IgG negative status	P value (I^2^)	Participants (n) (studies (n))	HR (95% CI) of seroreversion to MOG-IgG negative status	P value (I^2^)
Disease course						
Monophasic	382 (45)	1.0 (reference)	<0.0001 (52%)	135 (28)	1.0 (reference)	—
Relapsing	369 (45)	0.19 (0.14 to 0.26)			0.11 (0.034 to 0.39)	0.00049 (73%)‡
Sex						
Male	64 (29)	1.0 (reference)	0.03 (71%)		1.0 (reference)	—
Female	74 (29)	0.47 (0.29 to 0.92)			0.80 (0.36 to 1.8)	0.57
Age						
Paediatric (<18 years)	400 (38)	1.0 (reference)	0.53 (68%)		1.00 (reference)	—
Adult (≥18 years)	55 (38)	0.75 (0.31 to 1.8)			0.92 (0.28 to 3.1)	0.89
Not <18 years	55 (38)	1.0 (reference)	0.43 (68%)		—	—
<18 years	400 (38)	1.4 (0.60 to 3.3)				
Not 18–49 years	406 (38)	1.0 (reference)	0.92 (67%)		—	—
18–49 years	49 (38)	1.1 (0.43–2.5)				
Not ≥50 years	449 (38)	1.0 (reference)	1.0 (-1×10^8^%)§		—	—
≥50 years	6§ (38)	3.7×10^-10^ (0-∞)§				
Phenotype						
Not brain/brainstem	159 (32)	1.0 (reference)	0.46 (75%)		—	—
Brain/brainstem	24 (32)	1.5 (0.51 to 4.5)			1.0 (reference)	
Not ADEM	129 (32)	1.0 (reference)	0.040 (73%)		—	—
ADEM	54 (32)	2.6 (1.1 to 6.4)			1.2 (0.25 to 5.8)	0.83
Not ON	150 (32)	1.0 (reference)	0.47 (77%)		—	—
ON	33 (32)	1.3 (0.63 to 2.7)			0.59 (0.11 to 3.1)	0.53
Not TM	177 (32)	1.0 (reference)	0.51 (72%)		—	—
TM	6 (32)	1.5 (0.42 to 5.6)			0.50 (0.060 to 4.2)	0.53
Not ADEM+ON	164 (32)	1.0 (reference)	0.52 (73%)		—	—
ADEM+ON	19 (32)	0.71 (0.25 to 2.1)			1.8 (0.27 to 12)	0.54
Not ON+TM	167 (32)	1.0 (reference)	0.010 (75%)		—	—
ON+TM	16 (32)	0.22 (0.067 to 0.70)			0.16 (0.018 to 1.4)	0.091
Not mixed	152 (32)	1.0 (reference)	0.56 (74%)		—	—
Mixed	31 (32)	0.75 (0.28 to 2.0)			0.69 (0.19 to 2.5)	0.57

* Analysis was with one-stage IPD meta-analysis with univariable and multivariable mixed-effects Cox regression models.

† Minimum subgroup sample size criterion (n ≥5); phenotype ‘Other’ was excluded due to insufficient participant numbers (n=2 in univariable analysis and n=1 in multivariable analysis).

‡ I2=73% for entire multivariable analysis.

§ Results for the expanded age category analysis should be interpreted with caution due to instability in estimates for the ‘≥50’ group due to limited data; to address this limitation, an alternate analysis was performed by collapsing the age groups into ‘<18’ and ‘≥18’, which improved model stability.

ADEM, acute disseminated encephalomyelitis ; IPD, individual participant data; MOG-IgG, immunoglobulin G targeting myelin oligodendrocyte glycoprotein; ON, optic neuritis; TM, transverse myelitis.

**Figure 3:**
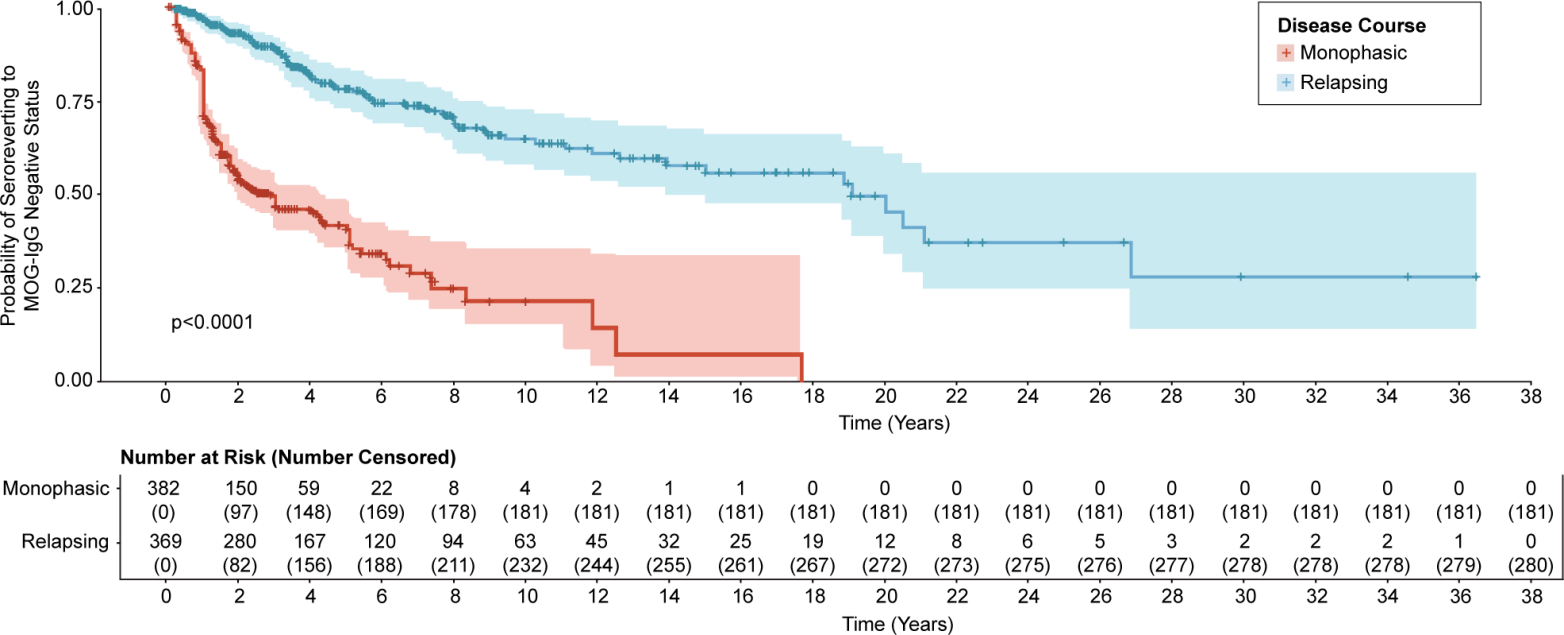
Kaplan-Meier survival curve of time to seroreversion to MOG-IgG negative status. MOG-IgG, immunoglobulin G targeting myelin oligodendrocyte glycoprotein.

Individuals with relapsing disease course became MOG-IgG seronegative significantly later than their monophasic counterparts (median 19 years vs 2.5 years, respectively, p<0.0001) (figure 3).

### Biomarkers of disease activity

**Figure 4:**
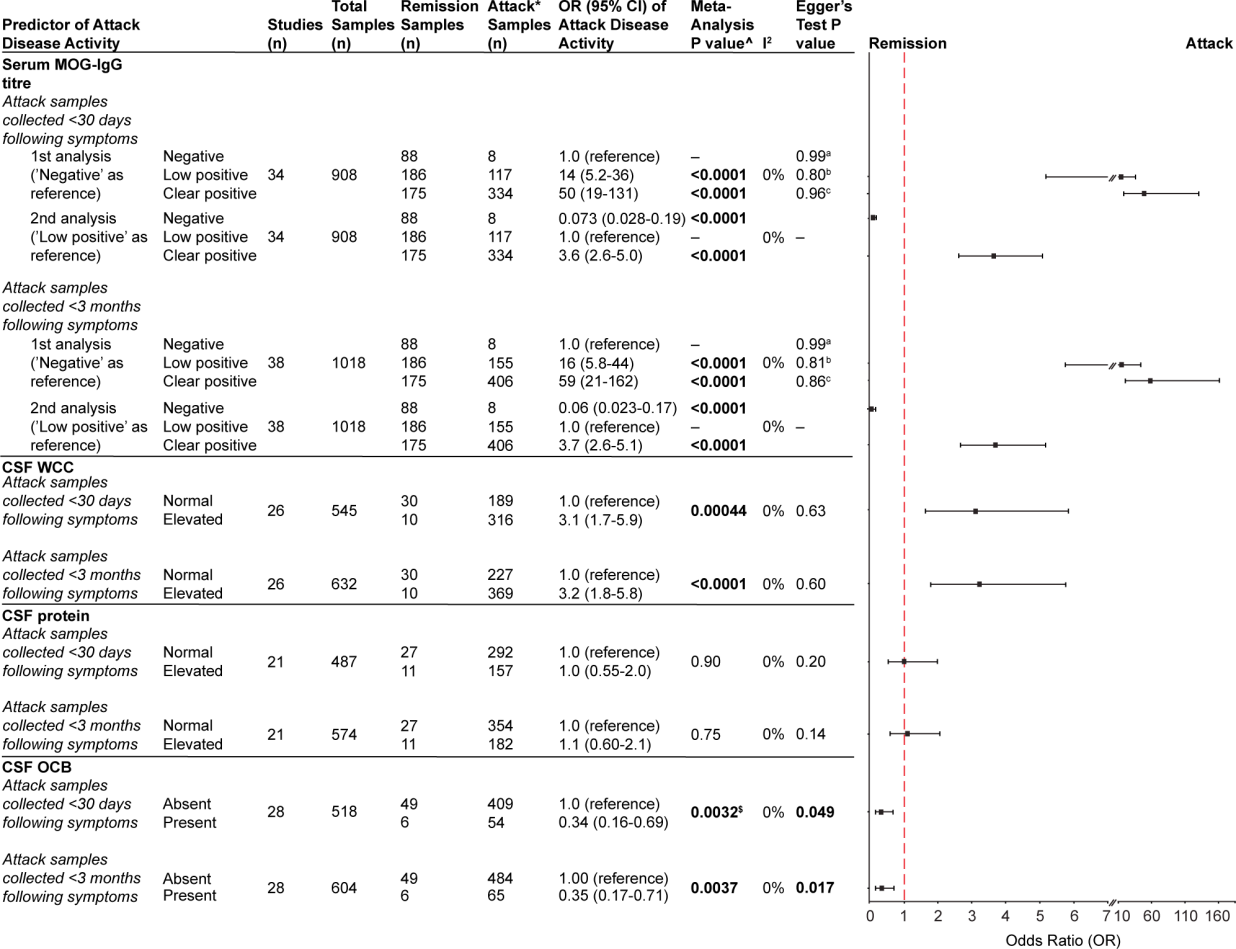
Investigating the association of biomarkers and attack disease activity. ˆAnalysis of serum MOG-IgG titre used one-stage IPD meta-analysis with mixed-effects logistic regression while analyses of CSF WCC, protein and OCB used univariable random-effects meta-analysis. *Attack samples included serum collected at disease onset and during relapse. ^a^Egger’s test comparison ‘negative’ versus ‘low positive’. ^b^Egger’s test comparison ‘negative’ versus ‘clear positive’. ^c^Egger’s test comparison ‘low positive’ versus ‘clear positive’. ^$^Due to convergence failure of this model with restricted maximum likelihood, the DerSimonian-Laird method was used for random-effects meta-analysis. CSF, cerebrospinal fluid; IPD, individual participant data; MOG-IgG, immunoglobulin G targeting myelin oligodendrocyte glycoprotein; OCB, oligoclonal band; WCC, white cell count.

Biomarkers were next assessed for capability to differentiate between remission and attack. The most frequently reported biomarkers, in descending order, were: (1) serum MOG-IgG titre, (2) CSF WCC, (3) CSF protein, (4) CSF OCB, (5) serum neurofilament light chain (NfL) and (6) serum glial fibrillary acidic protein (GFAP) (figure 4, table 3 and online supplemental table 8). It was common for individuals to have multiple serum MOG-IgG samples collected over the course of their disease and so titre was examined for its capability to correlate with disease activity. Serum MOG-IgG titre—categorised as negative, low positive or clear positive as per the latest international MOGAD diagnostic criteria^1^—was shown to effectively discriminate between remission (disease stability ≥30 days following symptoms) and attack (<30 days following symptoms). Low or clear positive titre was significantly associated with attack, while negative titre was significantly associated with remission (figure 4). This relationship persisted even when parameters were relaxed, with attack samples defined as those collected <3 months following symptoms (figure 4). No significant heterogeneity was observed (I^2^=0% for both analyses). In addition, the presence of CSF leucocytosis (≥5 cells/µL), compared with a normal WCC, was significantly associated with attack (figure 4). In contrast, the presence of CSF OCB, compared with the absence, was significantly associated with remission (figure 4). Egger’s test identified funnel plot asymmetry for CSF OCB analyses (figure 4 and online supplemental figure 2).

**Table 3:** Investigating serum GFAP and NfL biomarker association with disease activity

Predictor of disease activity	Study	Age category	Disease activity at time of sampling	Samples (n)	Mean±SD (pg/mL)	Mean difference (95% CI)	P value* (I^2^)
**GFAP**	Kim *et al*^37^	Adult	Remission	9	75±20	54 (−7.1 to 115) pg/mL higher at attack compared with remission	0.083 (79%)
			Attack†	7	90±22		
	Chang *et al*^45^	Combined	Remission	19	158±232		
			Attack†	23	310±367		
	Hyun *et al*^46^	Adult	Remission	54	107±47		
			Attack	17	186±81		
**NfL**	Kim *et al*^37^	Adult	Remission	9	11±5.0	47 (−2.1 to 97) pg/mL higher at attack compared with remission	0.061 (89%)
			Attack†	7	19±23		
	Chang *et al*^45^	Combined	Remission	19	44±78		
			Attack†	23	68±85		
	Hyun *et al*^46^	Adult	Remission	55	22±36		
			Attack	17	165±154		
	Luo *et al*^47^	Combined	Remission	47	13±12		
			Attack	22	60±61		

* Analysis was with random-effects meta-analysis of mean differences.

† Sample collected within 3 months of last clinical symptoms.

GFAP, glial fibrillar acidic protein; NfL, neurofilament light chain.

Both serum GFAP and serum NfL were measured at remission and at the time of clinically suspected MOGAD attack; however, neither biomarker appeared to effectively correlate with disease activity at time of sampling (table 3). Both analyses were characterised by considerable heterogeneity (I^2^=79% and I^2^=89%, respectively). Notably, 66% (2/3) of studies reporting GFAP and 50% (2/4) of studies reporting NfL defined attack samples as those collected <3 months following symptoms but, due to limited data, were analysed alongside studies which collected samples <30 days following symptoms. Heterogeneity was explored with subgroup analyses stratified by attack sample collection time (<30 days vs <3 months following symptoms) and sample size (n<10 vs n≥10). Regarding GFAP, all observed heterogeneity was accounted for by sample size (I^2^=0%, R^2^=100%). Regarding NfL, attack sample collection time accounted for approximately one-third of the initially observed heterogeneity (I^2^=73%, R^2^=35%), while sample size was not impactful (I^2^=81%, R^2^=0%). There were additional studies that reported serum GFAP or serum NfL levels collected at a single disease state (either attack or remission) and, thus, were not amenable to direct statistical comparison due to the lack of paired attack-remission data; however, random-effects meta-analysis of these means is presented (online supplemental table 8). Importantly, validated normative reference data for these biomarkers has not yet been established.

### Novel biomarkers of disease course and activity

Multiple novel biomarkers were assessed qualitatively and are described in online supplemental table 9. Serum NfL was most frequently reported (all relevant studies appear in online supplemental table 9, including those that were assessed earlier quantitatively). There was no consensus whether serum NfL demonstrated a significant attack-related association. Three studies were against (two with Class I evidence and one with Class III evidence) and three studies were in favour (two with Class I evidence and one with Class II evidence) (online supplemental table 9). Numerous other biomarkers were reported to significantly correlate with disease activity, including serum interleukin-1β (IL-1β), CSF microfibrillar-associated protein 4 (MFAP4), CSF-secreted ectodomain of soluble triggering receptor expressed on myeloid cells 2 (sTREM2), tumour necrosis factor alpha-induced protein 3 (TNFAIP3) and serum total thiol and native thiol (online supplemental table 9). Moreover, MOG epitope binding pattern, human leucocyte antigen genotype and peripheral blood mononuclear cells were highlighted as promising biomarkers for disease course prediction (online supplemental table 9).

**Table 4:** Summary of findings

Outcomes	Relative effect (95% CI)	P value	Studies (n)	Participants (n)	Quality of the evidence (GRADE)*	Recommendations
Persistent seropositivity† on serial serum MOG-IgG measurement was associated with relapsing disease course	OR 2.7 (1.8 to 4.0)	<0.0001	48	777	Moderate‡	Serial measurement of serum MOG-IgG at 3–6 month intervals in the 12 months following disease onset may be considered to stratify risk of relapsing disease course; however, increased burden of sample collection and cost are important caveats.
Relapsing disease course was associated with decreased likelihood of seroreverting to MOG-IgG negative status	HR 0.19 (0.14 to 0.26)	<0.0001	43	751	Moderate§	
Serum MOG IgG titre—categorised as negative, low positive, or clear positive—effectively discriminated between remission and attack, with a positive correlation between titre and odds of attack	Odds of attack with titre: Negative: OR 0.073 (0.028 to 0.19) Low: OR 1.0 (reference) Clear: OR 3.6 (2.6 to 5.0)	<0.0001	34	908	Moderate¶	Measurement of serum MOG-IgG titre may be diagnostically useful in settings of clinical uncertainty whereby an individual, who has an existing diagnosis of MOGAD, has symptoms that are not clearly attributable to a relapse of MOGAD.
CSF leucocytosis (≥5 cells/mL), compared with a normal WCC, was significantly associated with attack	OR 3.1 (1.7 to 5.9)	p=0.00044	26	545	Moderate**	Balancing the risk of lumbar puncture procedure, the presence of CSF leukocytosis may be a useful auxiliary finding to diagnose MOGAD attack; however, CNS infection must be effectively ruled out.

* GRADE working group grades of evidence: high quality: further research is very unlikely to change our confidence in the estimate of effect; moderate quality: further research is likely to have an important impact on our confidence in the estimate of effect and may change the estimate; low quality: further research is very likely to have an important impact on our confidence in the estimate of effect and is likely to change the estimate. Very low quality: we are very uncertain about the estimate.

† Persistent seropositivity was defined as ≥2 positive serum MOG-IgG results on serial measurement ≥3 months apart; individuals whose MOG-IgG measurements fluctuated between positive and negative were categorised as persistent seropositive if there were ≥2 positive results ≥3 months apart.

‡ Downgrade one point due to study design concerns as 38% (18/48) studies had ‘medium’ risk of bias, with the remaining having ‘low’ risk of bias.

§ Downgrade one point due to heterogeneity (I2=56%).

¶ Downgrade one point due to study design concerns as 34% (13/38) studies had ‘medium’ risk of bias, with the remaining having ‘low’ risk of bias.

** Despite no significant heterogeneity (I2=0%), downgraded one point due to study design concerns as 54% (14/26) studies had ‘medium’ risk of bias, with the remaining having ‘low’ risk of bias.

CNS, central nervous system; CSF, cerebrospinal fluid; GRADE, Grading of Recommendations Assessment, Development and Evaluation; MOGAD, myelin oligodendrocyte glycoprotein antibody-associated disease; MOG-IgG, immunoglobulin G targeting myelin oligodendrocyte glycoprotein.

Key outcomes of the quantitative component of this review are presented in table 4.

## Discussion

This systematic review and meta-analysis assessed the capability of laboratory biomarkers to predict MOGAD disease course and activity. Continued detection of serum MOG-IgG was significantly associated with a relapsing course. Furthermore, individuals with a relapsing course were significantly less likely to serorevert to MOG-IgG negative status. Categorisation of serum MOG-IgG titres as negative, low positive and clear positive per international MOGAD diagnostic criteria enabled comparison of measurements from various CBAs collected throughout the disease duration.^1^ Serum MOG-IgG titre effectively discriminated between remission and attack; there was a positive correlation between titre (clear positive >low positive) and attack and a clear association of negative titre with remission. Elevated CSF WCC was similarly associated with MOGAD attack. The presence of CSF OCB favoured remission state; however, this is likely to reflect the overall scarcity of OCB positivity in MOGAD. Many novel biomarkers were reported, with NfL and GFAP being the most frequent; however, based on the literature published within the timeframe of this systematic review, neither effectively correlated with disease activity. We have transparently reported the heterogeneity of the literature, especially with regards to timing of sample collection.

To date, serial serum MOG-IgG status has been the most thoroughly investigated biomarker candidate for disease course prediction, with some groups reporting a significant association between persistent seropositive measurements and relapsing disease course,^7^,^9^ while others did not.^6 10^ It is likely that limited sample sizes contributed to this lack of consensus. With meta-analysis, we demonstrated that relapsing disease course was associated with persistent seropositivity, lower likelihood of seroreversion to negative status and delayed seroreversion compared with monophasic individuals. Univariable analyses confirmed that relapsing course was significantly associated with persistent seropositivity on serial serum MOG-IgG measurements collected ≥3 months, ≥6 months and ≥12 months apart, reinforcing that relapsing individuals remained MOG-IgG seropositive for >7.5 times longer than monophasic individuals. Multivariable analyses that investigated this association alongside individual-level demographics (sex, age and phenotype) yielded significance when the sample collection interval to determine persistent seropositivity was set at ≥3 months but were likely underpowered at ≥6 months and ≥12 months. Overall, the data suggest that the highest rate of seroreversion to negative status occurred within 12 months following disease onset; hence, we support serial measurement of serum MOG-IgG at 3–6 month intervals in the 12 months following disease onset to stratify risk of relapsing disease course; however, increased burden of sample collection and cost are important caveats.

The International MOGAD Panel proposed criteria outlines the ‘low positive’ and ‘clear positive’ CBA categories to standardise serum MOG-IgG titres and to strengthen diagnostic criteria as individuals with low positive tests require at least one supportive clinical and/or radiological criteria for MOGAD diagnosis.^1^ Our meta-analysis observed a significant positive correlation between serum MOG-IgG titre and odds of attack (clear positive >low positive) as well as negative titre being significantly associated with remission. These findings highlight the value of serum MOG-IgG measurement in individuals with known diagnosis of MOGAD for settings of clinical uncertainty to discriminate whether symptoms are likely to represent a MOGAD attack as opposed to an alternate pathology. Pragmatically, the applicability of serum MOG-IgG titre as a biomarker of disease activity is limited by the following important caveats: (1) the range of titres within the clear positive category is extremely broad and further reference data to characterise additional thresholds appropriately sensitive and specific for prediction of attack or remission are currently lacking, (2) longitudinal sampling has revealed that titres may fluctuate independently of attacks,^10^ (3) international standardisation of all CBAs is required and (4) there is often a delay of days to weeks for result availability, which highlights that clinical diagnosis of demyelination remains the foundation on which acute treatment is initiated. Serum MOG IgG-titre, at this stage, is to confirm diagnosis of MOGAD attack and guide subsequent maintenance immunotherapy.

Lumbar puncture is a common investigation in the workup of MOGAD. Elevated CSF WCC, compared with normal CSF WCC, was significantly associated with MOGAD attack. Crucially, the most common aetiology of CSF leukocytosis is CNS infection, which requires emergent investigation, while autoimmune and neoplastic culprits occur less commonly.^19^ In addition, the presence of CSF OCB, compared with the absence, was significantly associated with remission. The overall positive rate of CSF OCB in all pooled samples was 14% (77/551). It is well-known that CSF OCB positivity occurs at a much lower rate in MOGAD than multiple sclerosis (MS), in which it is a core component of diagnosis.^3 20^ Our observed association of CSF OCB positivity with remission is biologically challenging to reconcile; it is likely that this estimate has been considerably impacted by the overall scarcity of CSF OCB positivity in MOGAD alongside limited remission CSF samples due to the relatively invasive nature of lumbar puncture and need to rationalise this procedure only in the setting of active disease. Egger’s test identified significant funnel plot asymmetry for CSF OCB analyses; visual inspection appeared to highlight potential sampling error with smaller studies. MOG-IgG synthesis appears to occur primarily in the periphery; thus, serum was the primary biospecimen focus of this review; however, intrathecal synthesis has also been reported.^21^,^24^ Individuals with intrathecal MOG-IgG synthesis may exhibit comparable clinical and pathological characteristics and, as such, the International MOGAD Panel proposed criteria supported CSF MOG-IgG testing in seronegative individuals whose pretest assessment was suggestive of MOGAD.^125^,^27^ Nonetheless, caution is warranted when interpreting CSF-restricted MOG-IgG in the absence of co-existing clinicoradiological features compatible with MOGAD, as alternative diagnoses such as MS and CNS vasculitis have been identified in over half the cases.^28^ Balancing this, paired serum and CSF MOG-IgG positivity as well as CSF-restricted MOG-IgG—in individuals with a typical MOGAD phenotype—has been associated with increased disease severity, thus demonstrating prognostic relevance, and is important for diagnostic utility in individuals with likely intrathecal synthesis of MOG-IgG underpinning their disease.^29^,^31^ Practically, MOG-IgG-specific antibody index, calculated using serum and CSF titres to assess MOG-IgG intrathecal synthesis, may enhance the clinical relevance of CSF MOG-IgG testing.^29^

Serum GFAP—an astrocyte-associated protein—has been identified as a useful candidate biomarker of disease activity in AQP4-IgG seropositive neuromyelitis optica spectrum disorder (NMOSD)—a primary astrocytopathy.^32^ In contrast, astrocytes appear to be largely unaffected in multiple histopathological studies of MOGAD specimens.^2133^,^35^ This aligns with our meta-analysis, which did not identify a significant association between serum GFAP and disease activity. Serum levels of NfL—an axon-associated protein—were also not found to be significantly associated with disease activity in this meta-analysis. Importantly, both analyses were weakened by considerable heterogeneity attributable to variable timing of sample collection and sample size. A challenge of these biomarkers is that elevated levels can occur with a multitude of neurological disorders such as stroke, traumatic brain injury and Parkinson’s disease^36^; however, interpretation of GFAP in conjunction with NfL may enhance their clinical relevance as patterns appear distinct at least between MOGAD and AQP4-IgG seropositive NMOSD.^37^ Since our literature search, a large retrospective cohort study has been published, which found that both serum GFAP and serum NfL were elevated in MOGAD at baseline with longitudinal decrement and that serum NfL values effectively predicted risk of relapse when samples were collected within 3 months of attack.^38^ Given this study was published outside the timeframe of our database search, we only conducted a preliminary analysis. Incorporation of this GFAP data found that GFAP levels were significantly elevated at attack compared with remission. NfL data were unable to be assessed as raw values could not be calculated from the published age-adjusted Z-scores. While serum NfL and GFAP currently remain largely restricted to the research setting, their ease of testing with commercially available kits is favourable for a potential future in diagnostics. Nonetheless, the lack of validated normative reference data and variability of results based on testing platform used may limit the ability to incorporate these data into future meta-analyses. Similar challenges apply for cytokines, such as IL-6, a potentially viable therapeutic target elevated in MOGAD compared with healthy controls,^39^ although with insufficient data for quantitative analysis in our study.

At this stage, serum MOG-IgG itself is the strongest biomarker of MOGAD disease course and activity. This highlights the MOG epitope binding pattern, capable of being tested at disease onset and highly stable over time, as a particularly promising biomarker candidate for disease course prediction; however, the current evidence is mixed. While some recent studies have observed a significant association of non-P42 MOG binding pattern with relapsing disease course,^40 41^ others have not.^42 43^ While the MOG epitope binding pattern has been shown to be highly stable over time, which would enable the use of serum collected at onset or remission prior to first relapse, thorough validation in the clinical setting is needed.^40 41 43^

Our systematic review with meta-analysis was the first of its kind to examine biomarkers of disease course and activity in MOGAD and, thus, these findings reflect the highest quality of evidence available. Nonetheless, our study was limited by the degree of heterogeneity in some of the analysed outcomes, likely due to between-study methodological diversity including treatment status, sample size, geographic location, sample collection timing and the presence of supportive radiological findings. In particular, treatment status at time of sample collection was not accounted for due to limited reporting; this methodological decision aimed to capture maximal relevant published works. We hypothesise that steroid-sparing immunosuppressants that act via mechanisms of B-cell and T-cell depletion would likely reduce serum MOG-IgG titre and promote seroreversion to negative status. Omission of treatment status at time of sample collection may have underestimated serum MOG-IgG titres as well as the longitudinal trends of serial MOG-IgG measurements. Additionally, many studies relied primarily on clinical and serological diagnosis of MOGAD and, thus, included individuals who did not necessarily have supportive MRI features. While this may have weakened diagnostic accuracy of some individuals included in our study, this does still align with the 2023 international MOGAD diagnostic criteria.

Finally, our findings may be extended to generate a tool capable of algorithmically incorporating individual-level clinical and laboratory parameters to estimate risk of relapse and guide personalised treatment recommendations. This would complement a recently proposed predictive risk score.^44^

## Conclusion

Our study supports the use of serial sampling of serum MOG-IgG as well as serum MOG-IgG titre and CSF WCC as biomarkers to stratify risk of relapsing disease course and to diagnose MOGAD attacks, respectively. Multiple novel biomarkers of disease course and activity have been identified and require further investigation. Our study highlights the need for future research, for which we propose the adoption of similar definitions for disease course, disease activity and sample collection timing to reduce the impact of between-study heterogeneity in future meta-analyses.

## Supplementary material

10.1136/jnnp-2025-337039 online supplemental file 1 online supplemental file

## Data Availability

Data are available upon reasonable request.
