# Supplementary material for: Biomarkers to predict relapse in myelin oligodendrocyte glycoprotein antibody-associated disease: a systematic review and meta-analysis
Source: J Neurol Neurosurg Psychiatry. 2025 Oct 1;97(2):e337039. doi: 10.1136/jnnp-2025-337039 (PMC12911599; doi:10.1136/jnnp-2025-337039)
Supplement: online supplemental file 1 [file jnnp-97-2-s001.docx]

**Biomarkers to predict relapse in myelin oligodendrocyte glycoprotein antibody-associated disease: a systematic review and meta-analysis**

***Andersen J et al***

**Online supplemental files**

**Online supplemental figure 1**: Forest plot demonstrating that persistent seropositivity on serial serum MOG-IgG measurement ≥3 months apart is associated with relapsing disease course (OR 2.7 (95% CI 1.8–4.0), p<0.0001). TS = transient seropositivity; PS = persistent seropositivity.

**Online supplemental figure 2**: Funnel plot asymmetry identified when investigating the association of CSF OCB and disease activity; (A) attack samples were collected <30 days following clinical symptoms, (B) attack samples were collected <3 months following clinical symptoms.

**Online supplemental table 1**: Systematic search strategy as of February 21, 2024. All terms were searched as text words and as exploded medical subject headings where possible. All terms within a concept were combined with “OR” and concepts were combined with “AND”.

**Online supplemental table 2**: Key definitions

**Online supplemental table 3**: Clinical phenotype categories

**Online supplemental table 4**: Characteristics of 106 studies included in systematic review

**Online supplemental table 5**: Comparison of follow-up duration between included monophasic and relapsing participants as reported by 48 studies. Statistical significance assessed with Mann-Whitney U Test.

**Online supplemental table 6**: Investigating the association of serial serum MOG-IgG measurement at various sample collection intervals and relapsing disease course.

**Online supplemental table 7**: Investigating the multivariable association of serial serum MOG-IgG measurement at various sample collection intervals and relapsing disease course alongside participant-level demographics.

**Online supplemental table 8**: Random-effects meta-analysis of means to investigate serum GFAP and NfL biomarker levels during different disease activity

**Online supplemental table 9**: Qualitative assessment of studies reporting novel biomarkers.

**Online supplemental References**


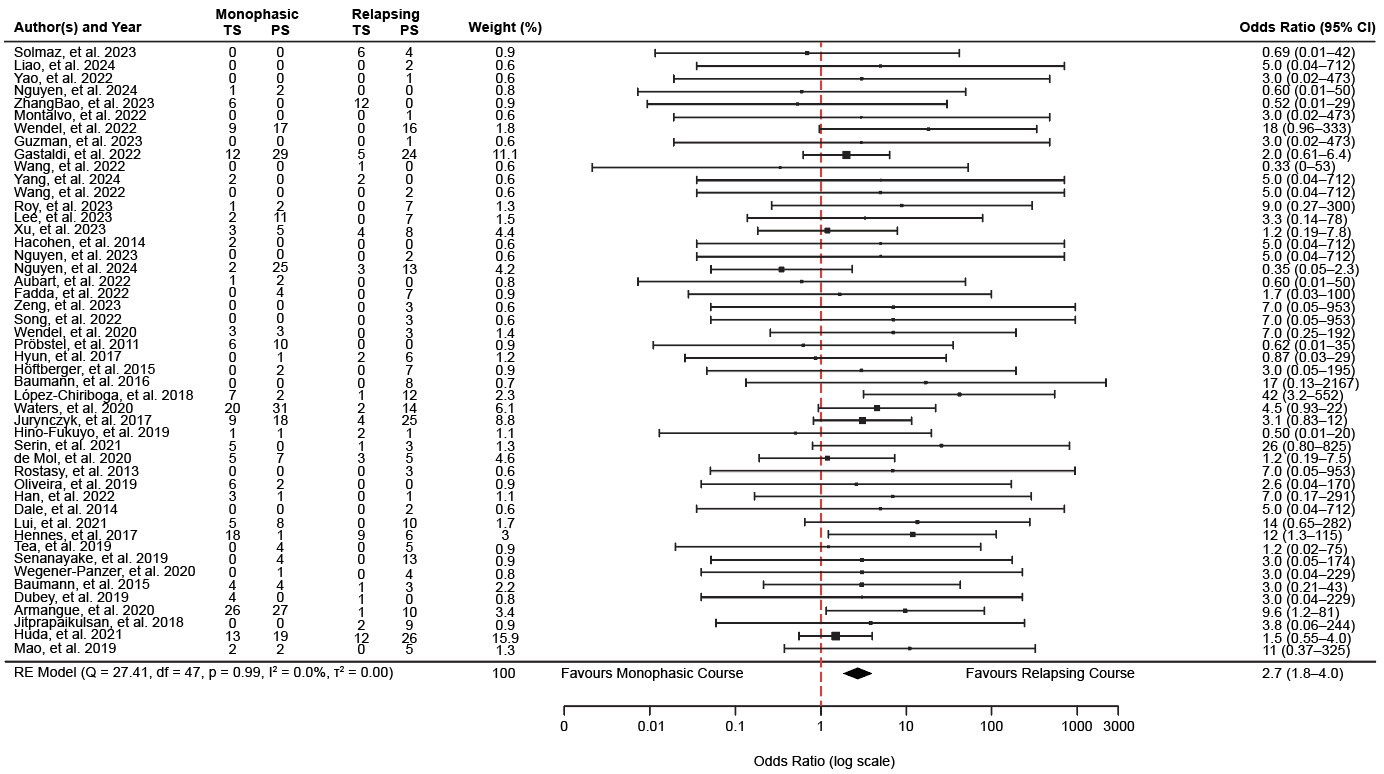


**Online supplemental figure 1: Forest plot demonstrating that persistent seropositivity on serial serum MOG-IgG measurement ≥3 months apart is associated with relapsing disease course (OR 2.7 (95% CI 1.8–4.0), p<0.0001)**. TS = transient seropositivity; PS = persistent seropositivity.


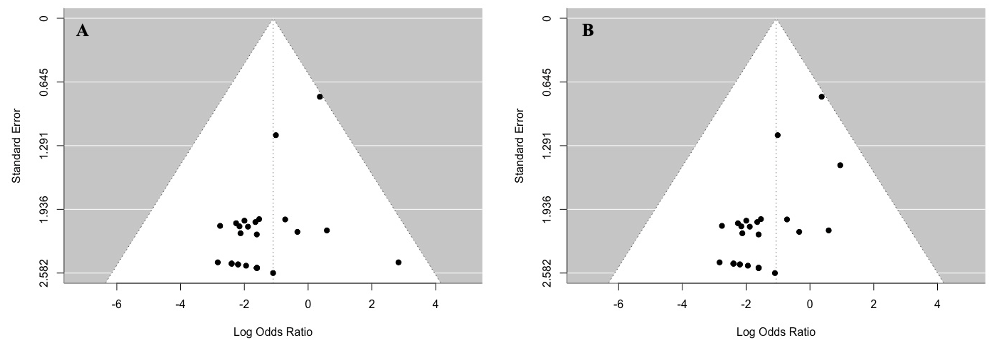


**Online supplemental figure 2: Funnel plot asymmetry identified when investigating the association of CSF OCB and disease activity**. (A) attack samples were collected <30 days following clinical symptoms. (B) attack samples were collected <3 months following clinical symptoms.

| **Online supplemental table 1: Systematic search strategy as of February 21, 2024.** All terms were searched as text words and as exploded medical subject headings where possible. All terms within a concept were combined with “OR” and concepts were combined with “AND”. | | | | |
| --- | --- | --- | --- | --- |
| Concept 1 | AND | Concept 2 | AND | Concept 3 |
| “myelin oligodendrocyte glycoprotein”  “myelin oligodendrocyte glycoprotein antibody associated disease”  “MOG antibody associated disease”  “MOGAD”  “MOG-AAD”  “myelin oligodendrocyte glycoprotein immunoglobulin”  “myelin oligodendrocyte glycoprotein antibody”  “MOG IgG”  “MOG Ab”  “acquired demyelinating syndrome”  “ADS” |  | “relaps*”  “recur*”  “monophasic”  “prognosis”  “disease progression”  “disease course”  “demyelinating attack”  “demyelinating episode”  “annualised relapse rate”  “annualized relapse rate”  “ARR” |  | “cell-based assay”  “CBA”  “immunoglobulin G”  “seropositive*”  “antibod*”  “IgG”  “cerebrospinal fluid”  “CSF”  “cytokine”  “chemokine”  “complement”  “neurofilament”  “pleocytosis”  “leukocytosis”  “lymphocytosis”  “monocytosis”  “oligoclonal band”  “OCB”  “titre”  “biomarker”  “IgG index”  “erythrocyte sedimentation rate”  “ESR”  “C reactive protein”  “CRP”  “inflammation”  “intrathecal”  “Epstein-Barr virus”  “EBV”  “Epstein-Barr virus nuclear antigen”  “EBNA”  “infectious mononucleosis” |

| **Online supplemental table 2: Key definitions** | |
| --- | --- |
| Term | Definition |
| Relapsing disease course | ≥2 episodes of new CNS symptoms or signs lasting >24 hours each and separated by ≥30 days, in the absence of other causes, and clinically and/or radiologically compatible with MOGAD episodes in an individual with ≥1 year of follow-up. |
| Monophasic disease course | 1 episode of new CNS symptoms or signs lasting >24 hours, in the absence of other causes, and clinically and/or radiologically compatible with a MOGAD episode in an individual with ≥1 year of follow-up. |
| Disease onset | <30 days following >24 hours of new CNS symptoms or signs, in the absence of other causes, and clinically and/or radiologically compatible with the first MOGAD episode. |
| Attack disease activity | <30 days following >24 hours of new CNS symptoms or signs, in the absence of other causes, and clinically and/or radiologically compatible with a MOGAD episode (including both onset and relapse events). |
| Remission disease activity | Disease stability ≥30 days following >24 hours of new CNS symptoms or signs, in the absence of other causes, and clinically and/or radiologically compatible with a MOGAD episode (after onset or relapse event). |
| Onset sample | Serum or CSF sample collected at disease onset. |
| First collected sample | The first collected serum or CSF sample for an individual, including but not limited to samples collected at disease onset and irrespective of disease activity at time of sampling. |
| Transient seropositivity | <2 positive serum MOG-IgG results on serial measurement ≥3 months apart.* |
| Persistent seropositivity | ≥2 positive serum MOG-IgG results on serial measurement ≥3 months apart. Individuals whose MOG-IgG measurements fluctuated between seropositive and seronegative were categorized as persistent seropositive if there were ≥2 positive results ≥3 months apart.* |
| MOG-IgG titre | Absolute MOG-IgG titres were semi-quantitatively categorised as ‘negative’, ‘low positive’, or ‘clear positive’ in accordance with Supplementary Table 5 of the international MOGAD diagnostic criteria.^1^ |
| Elevated CSF WCC | ≥5 cells/μL |
| Elevated CSF protein | >0.4g/L |

*≥3 months was defined as the primary sample collection interval to maximise the number of included studies; however, additional analyses utilising ≥6 and ≥12 month sample collection intervals were also explored.

| **Online supplemental table 3: Clinical phenotype categories** | |
| --- | --- |
| Phenotype | Comments |
| Brain/Brainstem | Included CCE as well as brainstem and cerebellar presentations but excluded ADEM. |
| ADEM | — |
| ON | — |
| TM | — |
| ON+TM | Included simultaneous ON and TM as well as isolated ON or TM with sequential presentation of the other. |
| ADEM+ON | Included simultaneous ADEM and ON as well as isolated ADEM or ON with sequential presentation of the other. |
| Mixed | Included simultaneous and/or sequential presentation of any combination of the above phenotype categories. |
| Other | Included demyelinating phenotypes with insufficient detail to be otherwise categorized e.g. ‘uncategorized relapsing MOGAD’ or ‘clinically isolated syndrome (CIS)’. |

| **Online supplemental table 4: Characteristics of 106 studies included in systematic review** | | | | | | | | | | | | | | |
| --- | --- | --- | --- | --- | --- | --- | --- | --- | --- | --- | --- | --- | --- | --- |
| **Source** | **MOG-IgG Seropositive Participants** | | | | | | **Cell-Based Assay (CBA)** | | | | **Contributed Biomarker(s)** | | **Risk of Bias** | **Assessment** |
|  | *Participants (n)* | *Age at Onset, Median (range) (years)* | *Sex*  *(n female [%])* | *Disease Course (n relapsing [%])* | *Clinical Phenotype (n)* | *Follow-up Duration, Median (range) (months)* | *Fixed or Live* | *Titre positivity threshold* | *Type of CBA* | *Centre* | *Disease Course Correlation* | *Disease Activity Correlation* |  |  |
| Baumann et al, 2016 ^2^  *Total* | 8 | 3 (1–7) | 5 [62.5] | 8 [100.0] | ADEM (8) | 48 (12-96) | Live | ≥1:160 | In-house CBA at Medical University of Innsbruck, Innsbruck, Austria | Medical University of Innsbruck, Innsbruck, Austria | (i) Serum MOG-IgG titre  (ii) Serial serum MOG-IgG status  (iii) CSF WCC  (iv) CSF protein  (v) CSF OCB | (i) Serum MOG-IgG titre  (ii) CSF WCC  (iii) CSF protein  (iv) CSF OCB | Medium | Quantitative |
| *Included* | 8 | 3 (1–7) | 5 [62.5] | 8 [100.0] | ADEM (8) | 48 (12-96) |  |  |  |  |  |  |  |  |
| Kwon et al, 2020 ^3^  *Total* | 21 | 45.6 (17-70) | 9 [42.9] | 13 [61.9] | ON (7), CRION (10), AQP4-IgG negative NMOSD (2), ADEM (2) | NA | Live | Semi-quantitative | In-house CBA at Seoul National University Hospital, Seoul, Republic of Korea | Seoul National University Hospital, Seoul, Republic of Korea | — | (i) Serum cytokines | Low | Qualitative |
| *Included* | 21 | 45.6 (17-70) | 9 [42.9] | 13 [61.9] | ON (7), CRION (10), AQP4-IgG negative NMOSD (2), ADEM (2) | NA |  |  |  |  |  |  |  |  |
| Kim et al., 2020 ^4^  *Total* | 16 | 40.5 (21-57)^$^ | 7 [43.8] | 8 [50.0] | ON (7), TM (3), Brain (2), ON+Brain (2), ON+TM+Brain (2) | NA | Fixed | >1:40 | Commercially available Euroimmun, Lubeck, Germany | Asan Medical Centre, Seoul, Republic of Korea | — | (i) Serum NfL  (ii) Serum GFAP  (iii) Serum tau | Low | Qualitative and quantitative |
| *Included* | 16 | 40.5 (21-57)^$^ | 7 [43.8] | 8 [50.0] | ON (7), TM (3), Brain (2), ON+Brain (2), ON+TM+Brain (2) | NA |  |  |  |  |  |  |  |  |
| Wendel et al, 2020 ^5^  *Total* | 22 | 7.5 (2-15)**^** | 10 [45.5] | 4 [18.2] | bON (16), ADEM (1), recurrent ON (2), ON+LETM (3) | 17 (4-141) | Live | ≥1:160 | In-house CBA at Medical University of Innsbruck, Innsbruck, Austria | Medical University of Innsbruck, Innsbruck, Austria | (i) Serum MOG-IgG titre  (ii) Serial serum MOG-IgG status | (i) Serum MOG-IgG titre | Medium | Quantitative |
| *Included* | 9 | NA | NA | 3 [33.3] | bON (6), bON+LETM (1), bON+uON (2) | 36 (12-141) |  |  |  |  |  |  |  |  |
| Pröbstel et al, 2011 ^6^  *Total* | 31 | 7 (1-16)^%^^ | 146 [58.2] | 11 [35.5] | ADEM (19), MS (10), CIS (2) | 44 (12-75)^%^ | Live | MCF > mean+4 SD of control (>1.45) | In-house CBA at Ludwig-Maximilians-University, Munich, Germany | Max Planck Institute of Neurobiology, Martinsried, Germany | (i) Serum MOG-IgG titre  (ii) Serial serum MOG-IgG status | (i) Serum MOG-IgG titre | Low | Quantitative |
| *Included* | 16 | NA | NA | 0 [0] | ADEM (16) | ≥12 |  |  |  |  |  |  |  |  |
| Hyun, et al., 2017 ^7^  *Total* | 22 | 30 (4-50) | 14 [63.6] | 17 [77.3] | Brain (2), ON (2), bON (2), TM (1), LETM (3), Brain+ON (3), Brain+bON (1), Brain+TM (2), Brain+LETM (1), ON+LETM (2), Brain+ON+LETM (1), Brain+ bON +TM (2) | 63 (7-200) | Live | ≥1 | Referred CBA at University of Oxford, UK | National Cancer Center, Republic of Korea | (i) Serum MOG-IgG titre  (ii) Serial serum MOG-IgG status  (iii) CSF OCB | — | Medium | Quantitative |
| *Included* | 19 | 30 (4-50) | 12 [63.2] | 15 [79.0] | Brain (2), ON (2), bON (2), TM (1), LETM (3), Brain+ON (3), Brain+bON (1), Brain+TM (1), Brain+LETM (1), Brain+ON+LETM (1), Brain+ bON +TM (2) | 61 (23-200) |  |  |  |  |  |  |  |  |
| Höftberger, et al., 2015 ^8^  *Total* | 17 | 27 (18-59) | 9 [52.9] | 10 [58.8] | ON (7), LETM (6), ON+LETM (5), ADEM (1) | 67 (11–415) | Live | ≥1:160 | In-house CBA at IDIBAPS, University of Barcelona, Spain | IDIBAPS, University of Barcelona, Spain | (i) Serum MOG-IgG titre  (ii) Serial serum MOG-IgG status  (iii) CSF WCC  (iv) CSF OCB | (i) Serum MOG-IgG titre  (ii) CSF WCC  (iii) CSF OCB | Low | Quantitative |
| *Included* | 15 | 27 (18-45) | 8 [53.3] | 10 [66.7] | ON (6), LETM (6), ADEM (1), ON+LETM (2) | 87 (17-415) |  |  |  |  |  |  |  |  |
| Horellou, et al., 2021 ^9^  *Total* | 12 | Monophasic: 10.2 ± 5.2; Relapsing: 9.9 ± 2.4* | 7 [58.3] | 7 [58.3] | ON (3), TM (6), ADEM (2), Brainstem (2)^#^ | Not available | Live and fixed | MFI > mean+6 SD of control (live) and ≥1:160 (fixed) | In-house CBA at Universite Paris-Saclay, Le Kremlin Bicetre, France | Universite Paris-Saclay, Le Kremlin Bicetre, France | (i) PBMCs | — | Low | Qualitative |
| *Included* | 12 | Monophasic: 10.2 ± 5.2; Relapsing: 9.9 ± 2.4* | 7 [58.3] | 7 [58.3] | ON (3), TM (6), ADEM (2), Brainstem (2)^#^ | Monophasic: 22.8 ± 26.4; Relapsing: 58.8 ± 26.4^&^ |  |  |  |  |  |  |  |  |
| Chang, et al., 2021 ^10^  *Total* | 42 | 27 (17-38)^$^ | 22 [52.4] | NA | ON (14), TM (3), Brain (10), ON+TM (3), ON+Brain (4), Brain+TM (7), ON+Brain+TM (1) | NA | Fixed | ≥1:32 | Commercially available Euroimmun, Lubeck, Germany | Huashan Hospital, Shanghai Medical College, Fudan University, Shanghai, China | — | (i) Serum NfL  (ii) Serum GFAP | Low | Qualitative and quantitative |
| *Included* | 42 | 27 (17-38)^$^ | 22 [52.4] | NA | ON (14), TM (3), Brain (10), ON+TM (3), ON+Brain (4), Brain+TM (7), ON+Brain+TM (1) | NA |  |  |  |  |  |  |  |  |
| Jarius, et al., 2020 ^11^  *Total* | 80 | 6 (0.6-17.7)^!^ | 45 [56.3] | 38 [47.5] | Attack phenotype (n=94 samples): TM+/-other symptoms (30), uON (15), bON (12), ADEM (34), Brainstem/Cerebellar (3) | 34.5 (0-229)^@^ | Live and fixed | NA | (i) Live CBA referred to Medical University Innsbruck, Austria, University of Vienna, Austria, and Ludwig Maximilian University Munich, Germany  (ii) Fixed CBA in-house at University of Heidelberg, Germany and commercially available Euroimmun, Lubeck, Germany | University of Heidelberg, Heidelberg, Germany | — | (i) CSF WCC  (ii) CSF protein  (iii) CSF OCB | Medium | Quantitative |
| *Included* | 80 | 6 (0.6-17.7)^!^ | 45 [56.3] | 38 [47.5] | Attack phenotype (n=94 samples): TM+/-other symptoms (30), uON (15), bON (12), ADEM (34), Brainstem/Cerebellar (3) | 34.5 (0-229)^@^ |  |  |  |  |  |  |  |  |
| Cobo-Calvo, et al., 2017 ^12^  *Total* | 27 | 16.8 (6.8–33.7)^$^ | 14 [51.9] | 11 [40.7] | ADEM (8), ON (8), TM (1), LETM (4), NMOSD (4), MS (2) | 17.8 (11.5–68.3)^$^ | Live | ≥1:640 | In-house CBA at Université Hospital of Lyon, France | Université Hospital of Lyon, France | (i) CSF WCC  (ii) CSF protein  (iii) CSF OCB | (i) Serum MOG-IgG titre | Medium | Quantitative |
| *Included* | 3 | 13 (3-16)^ | 1 [33.3] | 0 [0.0] | ADEM (2), TM (1) | 13 (12-24) |  |  |  |  |  |  |  |  |
| Hyun, et al., 2021 ^13^  *Total* | 15 | 38 (27-41)^$!^ | 6 [40.0] | 15 [100.0] | NA | 24 (21–43) | Live | NA | In-house CBA at National Cancer Center, Republic of Korea | National Cancer Center, Republic of Korea | — | (i) Serum NfL  (ii) Serum GFAP | Low | Qualitative and quantitative |
| *Included* | 15 | 38 (27-41)^$!^ | 6 [40.0] | 15 [100.0] | NA | 24 (21–43) |  |  |  |  |  |  |  |  |
| Ikeda, et al., 2019 ^14^  *Total* | 4 | 8 (3-12) | 2 [50.0] | 3 [75.0] | Brain+TM (1), ON+Brain+TM (1), bON+Brain+TM (1), bON+uON+Brain+TM (1) | 32.5 (18-87) | Live | ≥1:160 | Referred CBA to Tohoku University, Japan | Yokohama City University Medical Centre, Japan | (i) Serum MOG-IgG titre  (ii) CSF WCC  (iii) CSF protein  (iv) CSF OCB | (i) Serum MOG-IgG titre  (ii) CSF WCC  (iii) CSF protein  (iv) CSF OCB | Low | Quantitative |
| *Included* | 4 | 8 (3-12) | 2 [50.0] | 3 [75.0] | Brain+TM (1), ON+Brain+TM (1), bON+Brain+TM (1), bON+uON+Brain+TM (1) | 32.5 (18-87) |  |  |  |  |  |  |  |  |
| López-Chiriboga, et al., 2018 ^15^  *Total* | 25 | (i) 4 (2-9)  (ii) 6.5 (4-8)  (iii) 26 (22-45)  (iv) 22.5 (18-45) ^~^ | 15 [60.0] | 16 [64.0] | ADEM (9), ADEM+ON (7), ADEM+Brain (3), ADEM+ON+TM (2), ADEM+Brain+ON (2)^a^ | (i) 75 (15-236)  (ii) 32 (24-114)  (iii) 39 (10-161)  (iv) 16 (13-27) ^~^ | Live | ≥1:20 | In-house CBA at Mayo Clinic, USA | Mayo Clinic, USA | (i) Serum MOG-IgG titre  (ii) Serial serum MOG-IgG status | (i) Serum MOG-IgG titre | Low | Quantitative |
| *Included* | 24 | (i) 4 (2-9)  (ii) 6.5 (4-8)  (iii) 26 (22-45)  (iv) 22.5 (18-45) ^~^ | 14 [58.3] | 15 [62.5] | ADEM (9), ADEM+ON (7), ADEM+Brain (3), ADEM+ON+TM (2), ADEM+Brain+ON (2)^a^ | (i) 75 (15-236)  (ii) 32 (24-114)  (iii) 57 (15-161)  (iv) 16 (13-27) ^~^ |  |  |  |  |  |  |  |  |
| Waters, et al., 2020 ^16^  *Total* | 84 | 7.31 (4.93-10.57)^$^ | 46 [54.8] | 16/82 [19.5] | ADEM (32), ADEM+ON (3), ADEM+TM (9), ADEM+ON+TM (1), ON (34), TM (12), ON+TM (3), Other (3)^c^ | 6.74 (4.77-8.75)^$^ | Live | ≥1:200 | In-house CBA at University of Oxford, UK | University of Oxford, UK | (i) Serial serum MOG-IgG status | — | Low | Quantitative |
| *Included* | 67 | (i) 9.06 (6.60-13.36)^$^  (ii) 6.95 (5.28-9.96)^$^ | 33 [53.2] | 16 [23.9] | ADEM (22), ADEM+ON (2), ADEM+TM (4), ADEM+ON+TM (1), ON (24), TM (9), ON+TM (4), Other (3) | (i) 4.29 (3.00-5.96)^$^  (ii) 4.04 (2.99-6.01)^$b^  All ≥12-months |  |  |  |  |  |  |  |  |
| Jurynczyk, et al., 2017 ^17^  *Total* | 252 | 30.1 ± 18.3* | 144 [57.1] | 111 [44.1] | ON (44), TM (17), ADEM (32), ON+TM (26)^a^ | NA | Live | NA | In-house CBA at University of Oxford, UK | University of Oxford, UK | (i) Serial serum MOG-IgG status | — | Low | Quantitative |
| *Included* | 56 | NA | NA | 29 [51.8] | NA | 27.5 (12-438) |  |  |  |  |  |  |  |  |
| Hino-Fukuyo, et al., 2019 ^18^  *Total* | 5 | 5 (2-10) | 2 [40.0] | 3 [60.0] | ADEM (2), uON (1), bON (1), ADEM+bON (1) | 150 (68-322) | Live | NA | In-house CBA at Tohoku University, Japan | Tohoku University, Japan | (i) Serum MOG-IgG titre  (ii) Serial serum MOG-IgG status | (i) Serum MOG-IgG titre | Low | Quantitative |
| *Included* | 5 | 5 (2-10) | 2 [40.0] | 3 [60.0] | ADEM (2), uON (1), bON (1), ADEM+bON (1) | 150 (68-322) |  |  |  |  |  |  |  |  |
| Serin, et al., 2021 ^19^  *Total* | 9 | 6 (3-13) | 6 [66.7] | 4 [44.4] | ADEM (3), ON (2), CIS (1), ADEM+ON (1), Uncategorized relapsing MOGAD (2) | 28 (24-196) | Fixed | ≥1:10 | Commercially available Euroimmun, Lubeck, Germany | Ege University Medical Faculty, Izmir, Turkey | (i) Serum MOG-IgG titre  (ii) Serial serum MOG-IgG status  (iii) CSF WCC  (iv) CSF OCB | (i) Serum MOG-IgG titre | Low | Quantitative |
| *Included* | 9 | 6 (3-13) | 6 [66.7] | 4 [44.4] | ADEM (3), ON (2), CIS (1), ADEM+ON (1), Uncategorized relapsing MOGAD (2) | 28 (24-196) |  |  |  |  |  |  |  |  |
| Jarius, et al., 2020 ^20^  *Total* | 100 | 38 (18-78)^!^ | 58 [58.3] | NA | Attack phenotype (n=123 samples): TM+/-other symptoms (56), ON without TM (53), isolated brain or brainstem/cerebellar (11)^a^ | 29 (0-511)^@^ | Live and fixed | NA | (i) Live CBA referred to Medical University Innsbruck, Austria, University of Vienna, Austria, and Ludwig Maximilian University Munich, Germany  (ii) Fixed CBA in-house at University of Heidelberg, Germany and commercially available Euroimmun, Lubeck, Germany | University of Heidelberg, Heidelberg, Germany | — | (i) CSF WCC  (ii) CSF protein  (iii) CSF OCB | Medium | Quantitative |
| *Included* | 100 | 38 (18-78)^!^ | 58 [58.3] | NA | Attack phenotype (n=123 samples): TM+/-other symptoms (56), ON without TM (53), isolated brain or brainstem/cerebellar (11)^a^ | 29 (0-511)^@^ |  |  |  |  |  |  |  |  |
| Jarius, et al., 2016 ^21^  *Total* | 50 | 39 (range not stated) | 35 [70.0] | NA | ON (22), LETM (6), ON+TM (22) | NA | Live | ≥1:160 | In-house CBA at University of Heidelberg, Germany | University of Heidelberg, Germany | — | (i) Serum MOG-IgG titre | Low | Quantitative |
| *Included* | 75 samples; unclear participants | NA | NA | NA | NA | NA |  |  |  |  |  |  |  |  |
| Oliveira, et al., 2019 ^22^  *Total* | 31 | 33 (8-52) | 17 [54.8] | 23 [74.2] | bON (10), ON (11), Brainstem (2), LETM (4), ON+LETM (1), ON+TM (1), bON+TM (1), Brainstem+LETM (1) | 79 (38-104)^$^  All ≥12 months | Live | ≥1:128 | In-house CBA at HC-FMUSP, São Paulo, Brazil | HC-FMUSP, São Paulo, Brazil | (i) Serum MOG-IgG titre  (ii) Serial serum MOG-IgG status | — | Low | Quantitative |
| *Included* | 31 | 33 (8-52) | 17 [54.8] | 23 [74.2] | bON (10), ON (11), Brainstem (2), LETM (4), ON+LETM (1), ON+TM (1), bON+TM (1), Brainstem+LETM (1) | 79 (38-104)^$^  All ≥12 months |  |  |  |  |  |  |  |  |
| Arslan, et al., 2021 ^23^  *Total* | 8 | 32.1 ±10.0* | 3 [37.5] | 2 [25.0] | ON (6), TM (1), ON+TM (1) | NA | Fixed | ≥1:10 | Commercially available Euroimmun, Lubeck, Germany | Gazi University Faculty of Medicine, Ankara, Turkey | — | (i) Serum thiol | Low | Qualitative |
| *Included* | 8 | 32.1 ±10.0* | 3 [37.5] | 2 [25.0] | ON (6), TM (1), ON+TM (1) | NA |  |  |  |  |  |  |  |  |
| Liu, et al., 2020 ^24^  *Total* | 26 | (i) 48 ± 20.4*  (ii) 46 ± 19.7*^d^ | (i) 7 [58.3]  (ii) 14 [63.6] ^d^ | NA | Attack phenotype: ON (5), TM (5), Brain (3)^a^ | NA | Live | NA | In-house CBA at Chiba University, Chiba, Japan | Chiba University, Chiba, Japan | — | (i) PBMCs  (ii) CSF OCB | Low | Qualitative and quantitative |
| *Included* | 26 | (i) 48 ± 20.4*  (ii) 46 ± 19.7*^d^ | (i) 7 [58.3]  (ii) 14 [63.6] ^d^ | NA | Attack phenotype: ON (5), TM (5), Brain (3)^a^ | NA |  |  |  |  |  |  |  |  |
| de Mol, et al., 2020 ^25^  *Total* | 61 | 16.6 (7.9–31.9)^$^ | 28 [45.9] | 20 [32.8] | ON (7), bON (9), ADEM (14), Brainstem (1), TM (5), LETM (4), ON+ADEM (3), ON+Brain (1), ON+TM (13), ADEM+TM (1), CIS (1), RRMS (1)^a^ | 27.5 (1–329) | Live | MFI > 10 SD of control | In-house CBA at MS Centre ErasMS, Erasmus MC, Rotterdam, The Netherlands | MS Centre ErasMS, Erasmus MC, Rotterdam, The Netherlands | (i) Serial serum MOG-IgG status | — | Medium | Quantitative |
| *Included* | 20 | NA | NA | 8 [40.0] | NA | 30.5 (12-246) |  |  |  |  |  |  |  |  |
| Rostásy, et al., 2012 ^26^  *Total* | 17 | 10 (2-16) | 10 [58.8] | 12 [70.6] | uON (9), bON (3), uON+ADEM (2), MS (3) | 30 (11-74) | Live | ≥1:160 | In-house CBA at Medical University of Innsbruck, Innsbruck, Austria | Medical University of Innsbruck, Innsbruck, Austria | (i) Serum MOG-IgG titre  (ii) CSF OCB | (i) Serum MOG-IgG titre | Low | Quantitative |
| *Included* | 13 | 10 (2-16) | 7 [53.9] | 11 [84.6] | uON (9), bON (3), uON+ADEM (1) | 35 (19-74) |  |  |  |  |  |  |  |  |
| Rostásy, et al., 2013 ^27^  *Total* | 3 | 3 (3-14) | 3 [100] | 3 [100] | uON+TM (1), LETM+ON (2) | 28 (18-48) | Live | ≥1:160 | In-house CBA at Medical University of Innsbruck, Innsbruck, Austria | Medical University of Innsbruck, Innsbruck, Austria | (i) Serum MOG-IgG titre  (ii) Serial serum MOG-IgG status  (iii) CSF WCC  (iv) CSF protein  (v) CSF OCB | (i) Serum MOG-IgG titre  (ii) CSF WCC  (iii) CSF protein  (iv) CSF OCB | Low | Quantitative |
| *Included* | 3 | 3 (3-14) | 3 [100.0] | 3 [100.0] | uON+TM (1), LETM+ON (2) | 28 (18-48) |  |  |  |  |  |  |  |  |
| Saxena, et al., 2020 ^28^  *Total* | 24 | 4.5–52^e^^ | 15 [62.5] | NA | ADEM (1), ON+TM (2) ADEM+ON (7), ADEM+TM (1), MS (7), CIS (1), demyelinating neurological disease (1)^a^ | NA | Live | NA | Referred CBA to Harvard University, USA and Mayo Clinic, USA | Brigham and Women’s Hospital, Harvard Medical School, Boston, USA | — | (i) Serum TNFAIP3 | Low | Qualitative |
| *Included* | 24 | 4.5–52^e^^ | 15 [62.5] | NA | ADEM (1), ON+TM (2) ADEM+ON (7), ADEM+TM (1), MS (7), CIS (1), demyelinating neurological disease (1)^a^ | NA |  |  |  |  |  |  |  |  |
| Luo, et al., 2021 ^29^  *Total* | 49 | 25 (10-29.5)^$^ | 28 [57.1] | 34 [69.4] | Most recent attack phenotype: ON (23), Brain (15), TM (6), ON+Brain (2), ON+TM (2), Brain+TM (1) | 12^f^ | Live | NA | In-house CBA at the Third Affiliated Hospital of Sun Yat-Sen University, Guangzhou, China | The Third Affiliated Hospital of Sun Yat-Sen University, Guangzhou, China | — | (i) Serum NfL | Low | Qualitative and quantitative |
| *Included* | 49 | 25 (10-29.5)^$^ | 28 [57.1] | 34 [69.4] | Most recent attack phenotype: ON (23), Brain (15), TM (6), ON+Brain (2), ON+TM (2), Brain+TM (1) | 12^f^ |  |  |  |  |  |  |  |  |
| Tanaka, et al., 2020 ^30^  *Total* | 17 | 32 (8-76) | 10 [58.8] | 9 [52.9] | Onset attack: uON (10), bON (1), Brain (1), TM (4), ON+Brainstem (1) | NA | Live | NA | Referred CBA to Tohoku University School of Medicine, Sendai, Japan | Saitama Medical University, Kawagoe, Japan | — | (i) PBMCs | Low | Qualitative |
| *Included* | 17 | 32 (8-76) | 10 [58.8] | 9 [52.9] | Onset attack: uON (10), bON (1), Brain (1), TM (4), ON+Brainstem (1) | NA |  |  |  |  |  |  |  |  |
| Han, et al., 2022 ^31^  *Total* | 8 | 11 (1-17) | 3 [37.5] | 1 [12.5] | Brain (7), Brain+TM (1) | 15.5 (2-54) | Live | Borderline: >2.60–≤3.65;  Positive: >3.65 | In-house CBA at Seoul National University Children’s Hospital, Seoul, Republic of Korea | Seoul National University Children’s Hospital, Seoul, Republic of Korea | (i) Serum MOG-IgG titre  (ii) Serial serum MOG-IgG status  (iii) CSF WCC  (iv) CSF protein  (v) CSF OCB | — | Low | Quantitative |
| *Included* | 6 | 8 (1-17) | 3 [50.0] | 1 [16.7] | Brain (5), Brain+TM (1) | 18 (12-54) |  |  |  |  |  |  |  |  |
| Mariotto, et al., 2021 ^32^  *Total* | 18 | 34.5 (6-75) | 8 [44.4] | 7 [38.9] | ON (7), TM (3), Idiopathic demyelinating disorders (8) | 19 (3-93) | Live | ≥1:160 | In-house CBA at the University of Verona, Verona, Italy | University of Verona, Verona, Italy | — | (i) Serum NfL | Medium | Qualitative |
| *Included* | 18 | 34.5 (6-75) | 8 [44.4] | 7 [38.9] | ON (7), TM (3), Idiopathic demyelinating disorders (8) | 19 (3-93) |  |  |  |  |  |  |  |  |
| Sun, et al., 2020 ^33^  *Total* | 95 | 13 (2–67) | NA | NA | Onset attack: ON (58), Brain (30), Brainstem (6), TM (11), Other (4) | 12 (4–36)^&^ | Live | NA | In-house CBA at the Third Affiliated Hospital of Sun Yat-Sen University, Guangzhou, China | The Third Affiliated Hospital of Sun Yat-Sen University, Guangzhou, China | (i) HLA genotype | — | Low | Qualitative |
| *Included* | 95 | 13 (2–67) | NA | NA | Onset attack: ON (58), Brain (30), Brainstem (6), TM (11), Other (4) | 12 (4–36)^&^ |  |  |  |  |  |  |  |  |
| Mariotto, et al., 2019 ^34^  *Total* | 38 | 35.5 (6–75)^!^ | 16 [42.1] | 18 [47.4] | ON (14), ADEM (2), TM (4), ON+TM (4), Idiopathic demyelinating disorder (12), MS (2) | 19.5 (2–266) | Live | ≥1:160 | In-house CBA at the University of Verona, Verona, Italy | University of Verona, Verona, Italy | — | (i) Serum NfL | Low | Qualitative |
| *Included* | 38 | 35.5 (6–75)^!^ | 16 [42.1] | 18 [47.4] | ON (14), ADEM (2), TM (4), ON+TM (4), Idiopathic demyelinating disorder (12), MS (2) | 19.5 (2–266) |  |  |  |  |  |  |  |  |
| Alshamrani, et al., 2021 ^35^  *Total* | 9 | 35 (28-69) | 7 [77.8] | 6 [66.7] | ON (3), Brain (1), Brainstem (2), TM (1), LETM (2) | 48 (1-132) | NA | NA | Referred CBA to London (Ontario) MS clinic | King Fahad University Hospital, Imam Abdulrahman Bin Faisal University, Dammam, Saudi Arabia | (i) CSF OCB | — | Medium | Quantitative |
| *Included* | 5 | 43 (28-58) | 3 [60.0] | 4 [80.0] | ON (2), Brain (1), Brainstem (2) | 120 (13-132) |  |  |  |  |  |  |  |  |
| Dale, et al., 2014 ^36^  *Total* | 31 | 6.7 (2.0-15.3)^ | 18 [58.1] | 10 [32.3] | ON (9), ADEM (11), TM (4), MS (7) | 48 (3-164) | Live | MFI > mean+6 SD of control | In-house CBA at the Kids Research Institute at the Children’s Hospital at Westmead, Sydney Medical School, University of Sydney, Australia | The Kids Research Institute at the Children’s Hospital at Westmead, Sydney Medical School, University of Sydney, Australia | (i) Serum MOG-IgG titre  (ii) Serial serum MOG-IgG status  (iii) CSF OCB | (i) Serum MOG-IgG titre | Low | Quantitative |
| *Included* | 2 | 9.5 (5-14)^ | 1 [50.0] | 2 [100.0] | LETM+ON (1), ADEM+Brain (1) | 17.5 (16-19) |  |  |  |  |  |  |  |  |
| Lui, et al., 2021 ^37^  *Total* | 65 | 7.6 (4.3-10.9)^$^ | 36 [55.4] | NA | ADEM (2), ON+TM (1), CIS (1), RRMS (7), Demyelinating disorder not otherwise specified (54) | 42 (6-78)^$^ | Live | ≥1:20 | Referred CBA to Mayo Clinic, USA | University of California, USA | (i) Serum MOG-IgG titre  (ii) Serial serum MOG-IgG status | (i) Serum MOG-IgG titre | Low | Quantitative |
| *Included* | 25 | NA | NA | 9 [36.0] | NA | 60 (15-300) |  |  |  |  |  |  |  |  |
| Dubey, et al., 2019 ^38^  *Total* | 54 | 25 (3-73) | 24 [44.4] | NA | TM (21), ON+TM (32), ADEM+TM (1) | 24 (2-120) | Live | ≥1:20 | Referred CBA to Mayo Clinic, USA | Mayo Clinic, USA | (i) Serial serum MOG-IgG status | — | Medium | Quantitative |
| *Included* | 5 | NA | NA | 1 [20.0] | NA | 35 (15-120) |  |  |  |  |  |  |  |  |
| Hennes, et al., 2017 ^39^  *Total* | 65 | (i) 9 (3–15)  (ii) 5 (0–17)^g^^ | 35 [53.9] | 25 [38.5] | ON (8), ADEM (22), ADEM+ON (11), ON+TM (9), CIS (12), MS (3) | 24^f^ | Live | ≥1:160 | Referred CBA to Medical University of Innsbruck, Innsbruck, Austria | Olga Hospital, Stuttgart, Germany | (i) Serum MOG-IgG titre  (ii) Serial serum MOG-IgG status  (iii) CSF OCB | (i) Serum MOG-IgG titre  (ii) CSF OCB | Low | Quantitative |
| *Included* | 62 | NA | NA | 22 [35.5] | ON (8), ADEM (22), ADEM+ON (11), ON+TM (9), CIS (12) | 24^f^ |  |  |  |  |  |  |  |  |
| Tea, et al., 2019 ^40^  *Total* | 287 | 22 (8–43)^$^ | 165 [57.5]^i^ | 105 [43.6]^j^ | uON (62), bON (58), mixed ON (9), ADEM (54), LETM (18), ADEM+ON (10), ADEM+LETM (1), Brainstem+LETM (1), Brainstem+ON+TM (1), ON+TM (9), uON+LETM (2), TM (6), bON+LETM (2), bON+LETM+ADEM (2), CIS (1), Other (12), Unknown (39) | NA | Live | MFI > mean+6 SD of control | In-house CBA at the Kids Research Institute at the Children’s Hospital at Westmead, Sydney Medical School, University of Sydney, Australia | The Kids Research Institute at the Children’s Hospital at Westmead, Sydney Medical School, University of Sydney, Australia | (i) Serial serum MOG-IgG status  (ii) CSF WCC  (iii) CSF protein  (iv) CSF OCB | (i) CSF WCC  (ii) CSF protein  (iii) CSF OCB | Low | Quantitative |
| *Included* | 16 | 13 (3-45)^$h^ | 5 [71.4]^i^ | 8 [50.0] | uON (1), bON (3), ADEM (6), ADEM+ON (2), ADEM+LETM (1), Brainstem+LETM (1), ON+LETM (2) | 34 (17-99) |  |  |  |  |  |  |  |  |
| Senanayake, et al., 2019 ^41^  *Total* | 126 | 26 (3–68) | 70 [55.6] | 43 [34.1] | ON (58), TM (24), ADEM (12), NMOSD (32) | 48 (1–240) | NA | NA | Clinical Laboratory Improvement Amendments approved flow cytometric assays | The National Hospital of Sri Lanka, Colombo, Sri Lanka | (i) Serial serum MOG-IgG status | — | Low | Quantitative |
| *Included* | 17 | NA | NA | 13 [76.5] | NA | (i) Monophasic: 12 (12-24)  (ii) Relapsing: 84 (36-204) |  |  |  |  |  |  |  |  |
| Wegener-Panzer, et al., 2020 ^42^  *Total* | 10 | 8 (4–16) | 4 [40.0] | 5 [50.0] | Brain (9), Brain+ON (1) | 18 (6-48) | Live and fixed | (i) Live: ≥1:160  (ii) Fixed: NA | (i) Live: referred CBA to Medical University of Innsbruck, Innsbruck, Austria  (ii) Fixed: commercially available Euroimmun, Lubeck, Germany | Children’s Hospital Datteln, University Witten/Herdecke, Germany | (i) Serum MOG-IgG titre  (ii) Serial serum MOG-IgG status  (iii) CSF WCC  (iv) CSF OCB | (i) Serum MOG-IgG titre  (iii) CSF WCC  (iv) CSF OCB | Medium | Quantitative |
| *Included* | 6 | 6.5 (3-16) | 3 [50.0] | 4 [66.7] | Brain (5), Brain+ON (1) | 33 (12-48) |  |  |  |  |  |  |  |  |
| Zhou, et al., 2019 ^43^  *Total* | 23 | 5.38 (2.33–12.75) | 13 [56.5] | 23 [100.0] | ADEM (2), ADEM+ON (3), NMOSD (11), Uncategorized CNS demyelination (7) | ≥12 | Fixed | NA | Commercially available Euroimmun, Lubeck, Germany | Peking University First Hospital, Beijing, China | (i) CSF OCB | (i) CSF WCC  (ii) CSF protein  (iii) CSF OCB | Low | Quantitative |
| *Included* | 23 | 5.38 (2.33–12.75) | 13 [56.5] | 23 [100.0] | ADEM (2), ADEM+ON (3), NMOSD (11), Uncategorized CNS demyelination (7) | ≥12 |  |  |  |  |  |  |  |  |
| Keller, et al., 2021 ^44^  *Total* | 109 | (i) Paediatric: 9 (1-17)  (ii) Adult: 38 (18–70 | 70 [64.2] | 38 [34.9] | uON (36), bON (19), LETM (14), ADEM (40) | NA | Live | (i) University of Sydney: MFI > mean + 3 SD of control  (ii) Medical University of Innsbruck: ≥1:160 | CBA referred to The Kids Research Institute at the Children’s Hospital at Westmead, Sydney Medical School, University of Sydney, Australia and Innsbruck Medical University, Innsbruck, Austria | University Hospital Münster, Münster, Germany | — | (i) Serum complement | Low | Qualitative |
| *Included* | 109 | (i) Paediatric: 9 (1-17)  (ii) Adult: 38 (18–70 | 70 [64.2] | 38 [34.9] | uON (36), bON (19), LETM (14), ADEM (40) | NA |  |  |  |  |  |  |  |  |
| Baumann, et al., 2015 ^45^  *Total* | 19 | 4 (1-17) | 9 [47.4] | 4 [21.1] | ADEM (16), ADEM+ON (3) | 27 (5-81) | Live | ≥1:160 | In-house CBA at Medical University of Innsbruck, Innsbruck, Austria | Medical University of Innsbruck, Innsbruck, Austria | (i) Serum MOG-IgG titre  (ii) Serial serum MOG-IgG status  (iii) CSF WCC  (iv) CSF OCB | (i) Serum MOG-IgG titre  (ii) CSF WCC  (iii) CSF OCB | Low | Quantitative |
| *Included* | 14 | 4 (1-17) | NA | 4 [28.6] | ADEM (11), ADEM+ON (3) | 33.5 (14-81) |  |  |  |  |  |  |  |  |
| Ramanathan, et al., 2018 ^46^  *Total* | 59 | 12 (1-74) | 40 [67.8] | 59 [100.0] | Brainstem/Cerebellar (1), bON (3), uON (8), uON+bON (6), ADEM+Brainstem (2), bON+Brain (2), ADEM+Brainstem/Cerebellar (1), ADEM+Cerebellar (1), ADEM+bON (2), ADEM+uON (3), ADEM+LETM (2), bON+LETM (3), uON+Brain (2), uON+LETM (2), Cerebellar+Sensory non-spinal (1), bON+Sensory non-spinal (2), TM+LETM (1), uON+Brainstem (1), uON+bON+Brain (1), uON+Brainstem+TM (2), uON+Brainstem/Cerebellar+LETM (1), ADEM+Cerebellar+uON (2), uON+TM+Sensory non-spinal (1), ADEM+LETM+nonencephalitic ADS (1), bON+uON+TM (2), ADEM+uON+bON+LETM (1), ADEM+Brain+uON+bON (1), ADEM+Brainstem/Cerebellar+uON+LETM (1), bON+uON+LETM+Brainstem (1), ADEM+Brainstem/Cerebellar+bON+uON (1), bON+uON+TM+LETM (1) | 45 (12–288) | Live | MFI > mean + 3 SD of control | In-house CBA at the Kids Research Institute at the Children’s Hospital at Westmead, Sydney Medical School, University of Sydney, Australia | The Children’s Hospital at Westmead, Sydney Medical School, University of Sydney, Australia | (i) CSF WCC  (ii) CSF protein  (iii) CSF OCB | (i) CSF WCC  (ii) CSF protein  (iii) CSF OCB | Low | Quantitative |
| *Included* | 51 | 10 (1-74) | 35 [68.6] | 51 [100.0] | Brainstem/Cerebellar (1), bON (3), uON (1), uON+bON (6), ADEM+Brainstem (2), bON+Brain (2), ADEM+Brainstem/Cerebellar (1), ADEM+Cerebellar (1), ADEM+bON (2), ADEM+uON (3), ADEM+LETM (2), bON+LETM (3), uON+Brain (2), uON+LETM (2), Cerebellar+Sensory non-spinal (1), bON+Sensory non-spinal (1), TM+LETM (1), uON+Brainstem (1), uON+bON+Brain (1), uON+Brainstem+TM (2), uON+Brainstem/Cerebellar+LETM (1), ADEM+Cerebellar+uON (2), uON+TM+Sensory non-spinal (1), ADEM+LETM+nonencephalitic ADS (1), bON+uON+TM (2), ADEM+uON+bON+LETM (1), ADEM+Brain+uON+bON (1), ADEM+Brainstem/Cerebellar+uON+LETM (1), bON+uON+LETM+Brainstem (1), ADEM+Brainstem/Cerebellar+bON+uON (1), bON+uON+TM+LETM (1) | 49 (12-288) |  |  |  |  |  |  |  |  |
| Benetou, et al., 2020 ^47^  *Total* | 17 | 6.3 (4.1–9.6)^$^^ | 9 [52.9] | NA | NA | NA | NA | NA | NA | Evelina London Children's Hospital, Guys and St. Thomas' Hospital NHS Foundation Trust, Kings Health Partners, London, UK | — | (i) Peripheral blood count index ratios | Low | Qualitative |
| *Included* | 17 | 6.3 (4.1–9.6)^$^^ | 9 [52.9] | NA | NA | NA |  |  |  |  |  |  |  |  |
| Armangue, et al., 2020 ^48^  *Total* | 116 | 6·2 (3·7–10·0)^$^^ | 57 [49.1] | 33 [28.5] | ADEM (42), ON (5), uON (8), bON (6), Brain (18), LETM (9), ADEM+ON (4), Brain+ON (3), ON+Other (1), NMOSD (11), MS (5), Other (4) | 42 (8–197) | Live | ≥1:160 | In-house CBA at IDIBAPS, Hospital Clínic, Universitat de Barcelona, Barcelona, Spain | IDIBAPS, Hospital Clínic, Universitat de Barcelona, Barcelona, Spain | (i) Serial serum MOG-IgG status | — | Low | Quantitative |
| *Included* | 64 | NA | NA | 11 [17.2] | NA | 24 (12-24) |  |  |  |  |  |  |  |  |
| Nakajima, et al., 2015 ^49^  *Total* | 8 | 31 (16-65)^ | 6 [75.0] | 3 [37.5] | ON (8) | 26.5 (3-53) | Fixed | ≥1:10 | In-house CBA at Nagasaki University Hospital, Nagasaki, Japan | Nagasaki University Hospital, Nagasaki, Japan | (i) CSF WCC  (ii) CSF protein  (iii) CSF OCB | — | Low | Quantitative |
| *Included* | 7 | 25 (15-55)^k^^ | 5 [71.4] | 3 [42.9] | ON (7) | 30 (15-53) |  |  |  |  |  |  |  |  |
| Mao, et al., 2019 ^50^  *Total* | 25 | 6.6 (3–12.4) | 13 [52.0] | 10 [40.0] | ADEM (9), ON (1), bON (5), Brain (4), TM (1), LETM (1), CIS (1), ADEM+ON (1), ADEM+uON (1), ADEM+TM (1) | 15 (7–63) | Fixed | NA | Commercially available Euroimmun, Lubeck, Germany | Xiangya Hospital, Central South University, Changsha, China | (i) Serum MOG-IgG titre  (ii) Serial serum MOG-IgG status  (iii) CSF WCC  (iv) CSF protein  (v) CSF OCB | (i) Serum MOG-IgG titre  (ii) CSF WCC  (iii) CSF protein  (iv) CSF OCB | Medium | Quantitative |
| *Included* | 20 | 6 (3-12) | 10 [50.0] | 10 [50.0] | ADEM (8), ON (1), bON (3), Brain (2), TM (1), LETM (1), CIS (1), ADEM+ON (1), ADEM+uON (1), ADEM+TM (1) | 15.5 (12-63) |  |  |  |  |  |  |  |  |
| Siritho, et al., 2016 ^51^  *Total* | 6 | 33 (18-57) | 5 [83.3] | 5 [83.3] | bON (2), uON (2), bON+TM (1), uON+Brain+LETM (1) | NA | NA | NA | Referred CBA to Tohoku University, Japan | MS and Related Disorders Clinic, Siriraj Hospital, Mahidol University, Bangkok, Thailand | (i) Serum MOG-IgG titre  (ii) CSF OCB | — | Medium | Quantitative |
| *Included* | 4 | 33.5 (19-57) | 3 [75.0] | 4 [100.0] | bON (1), uON (1), bON+TM (1), uON+Brain+LETM (1) | 18 (13-34) |  |  |  |  |  |  |  |  |
| Zhang, et al., 2021 ^52^  *Total* | 34 | 6 (1-14) | 16 [47.1] | 8 [23.5] | ADEM (18), ON (7), NMOSD (4), LETM (2), CIS (1), Brain (2) | 34.5 (14–63) | Fixed | ≥1:10 | Commercially available Euroimmun, Lubeck, Germany | Children’s Hospital of Fudan University, National Children’s Medical Center, Shanghai, China | (i) CSF WCC  (ii) CSF protein | (i) CSF WCC  (ii) CSF protein | Low | Quantitative |
| *Included* | 31 | (i) Monophasic: median 5.8 (1.6–13.6)  (ii) Relapsing: 6.7 (2.9–14.2) | NA | 6 [19.4] | NA | (i) Monophasic: 33 (14–57)  (ii) Relapsing: 42.5 (16–63) |  |  |  |  |  |  |  |  |
| Jitprapaikulsan, et al., 2018 ^53^  *Total* | 31 | 32 (7-66) | 15 [48.4] | 31 [100.0] | ON (27), ON+other CNS demyelination (3) | (i) 44.6 (19.7-64.8)^$^  (ii) 75.4 (41.3-183.1)^$l^ | Live | ≥1:20 | In-house CBA at Mayo Clinic, USA | Mayo Clinic, USA | (i) Serum MOG-IgG titre  (ii) Serial serum MOG-IgG status | (i) Serum MOG-IgG titre | Low | Quantitative |
| *Included* | 11 | NA | NA | 11 [100.0] | ON (11) | 27 (12-237) |  |  |  |  |  |  |  |  |
| Huda, et al., 2021 ^54^  *Total* | 76 | 27 (19–45)^$^ | 41 [54.0] | 42 [55.3] | Onset attack phenotype by site: ADEM (5), ON (46), bON (27), TM (32), LETM (21), ON+TM (14), Brain (19) | 49 (28–113)^$^ | Live | NA | Referred CBA to University of Oxford, UK | The Walton Centre NHS Foundation Trust, Liverpool, UK | (i) Serial serum MOG-IgG status  (ii) CSF OCB | — | Low | Quantitative |
| *Included* | 75 | NA | NA | 41 [54.7] | NA | 51 (12-400) |  |  |  |  |  |  |  |  |
| Dauby, et al., 2021 ^55^  *Total* | 8 | 27.7 (9.8–39.5) | 4 [50.0] | 5 [62.5] | ON (7), ON+TM (1) | 90 (16.8-394.8)^m^ | Live | NA | Referred CBA to University of Oxford, UK | University Hospital of Liège, Liège, Belgium | (i) CSF OCB | — | Low | Quantitative |
| *Included* | 8 | 27.7 (9.8–39.5) | 4 [50.0] | 5 [62.5] | ON (7), ON+TM (1) | 90 (16.8-394.8)^m^ |  |  |  |  |  |  |  |  |
| Solmaz, et al., 2023 ^56^  *Total* | 10 | 7.5 (2–16.5) | 8 [80.0] | 10 [100.0] | Onset attack: bON (1), TM (6), ADEM (3) | 78 (30–216) | Live and fixed | NA | NA | Etlik City Hospital, Ankara, Turkey | (i) Serum MOG-IgG titre  (ii) Serial serum MOG-IgG status  (iii) CSF WCC  (iv) CSF protein  (v) CSF OCB | (i) Serum MOG-IgG titre | Low | Quantitative |
| *Included* | 10 | 7.5 (2–16.5) | 8 [80.0] | 10 [100.0] | Onset attack: bON (1), TM (6), ADEM (3) | 78 (30–216) |  |  |  |  |  |  |  |  |
| Seok, et al., 2023 ^57^  *Total* | 55 | 39.7 ± 17.2* | 29 [52.7] | 37/47 [78.7] | Onset attack: ON (38), TM (5), Brainstem (7), Brain or ADEM (4), Poly-regional (1) | NA | Live | ≥1 | In-house CBA at Soonchunhyang University Hospital Cheonan, Cheonan, Republic of Korea | Soonchunhyang University Hospital Cheonan, Cheonan, Republic of Korea | (i) Serum MOG-IgG epitope | — | Low | Qualitative |
| *Included* | 47 | NA | NA | 37 [78.7] | NA | ≥12 |  |  |  |  |  |  |  |  |
| Martin, et al., 2024 ^58^  *Total* | 58 | 24.3 (1.2–77.3) | 26 [44.8] | 36 [62.1] | Onset attack: ON (35), TM (4), ADEM (10), Brain (3), NMOSD (1), Brainstem/Cerebellar (1), Other (4) | (i) 32.8 (31.0-38.5)^$^  (ii) 50.8 (17.5-206.4)^$^  (iii) 29.3 (22.6–44.3)^$^  (iv) 47.0 (29.8–90.1)^$^ | NA | NA | NA | Oregon Health & Science University, Portland, OR, USA | — | (i) Serum MOG-IgG titre  (ii) CSF WCC  (iii) CSF protein  (iv) CSF OCB | Low | Quantitative |
| *Included* | 104 serum and 56 CSF samples; unclear participants | NA | NA | NA | NA | NA |  |  |  |  |  |  |  |  |
| Liao, et al., 2024 ^59^  *Total* | 12 | 8.5 (2-12) | 8 [72.7] | 9 [75.0] | Brain+uON (1), Brain (8), Brain+bON (2), Brain+bON+uON (1) | NA | Fixed | NA | Commercially available Euroimmun, Lubeck, Germany | Xiangya Hospital of Central South University, Changsha, China | (i) Serum MOG-IgG titre  (ii) Serial serum MOG-IgG status  (iii) CSF OCB | (i) Serum MOG-IgG titre  (ii) CSF OCB | Medium | Quantitative |
| *Included* | 6 | 9 (7-12) | 5 [83.3] | 6 [100.0] | Brain+uON (1), Brain (3), Brain+bON (2) | 32 (range 25.5-68.9) |  |  |  |  |  |  |  |  |
| Nosadini, et al., 2023 ^60^  *Total* | 75 | 7 (1.8–18.6) | 40 [53.3] | 26/65 [40.0] | ADEM (30), ON ± CNS lesions (27), CNS demyelination (6), ADEM+ON (4), NMOSD (3), CIS (2), Brain (2), LETM (1) | 30 (1–130) | Live and fixed | NA | NA | University Hospital of Padova, Italy | (i) CSF WCC  (ii) CSF protein  (iii) CSF OCB | (i) CSF WCC  (ii) CSF protein  (iii) CSF OCB | Medium | Quantitative |
| *Included* | 51 | NA | NA | 18 [35.3] | NA | ≥12 |  |  |  |  |  |  |  |  |
| Kang, et al., 2023 ^61^  *Total* | 48 | 6.57 ± 3.02* | 30 [62.5] | 11 [22.9] | ADEM (24), NMOSD (4), Autoimmune Encephalitis Overlap Syndrome (8), ON (5), Cranial neuritis (2), Brain (2), Meningitis (2), Demyelinating pseudotumor (1) | ≥12 | NA | ≥1:10 | Referred CBA to Guangzhou Medical Laboratory Center and Kindstar Medical Laboratory, China | Hunan Children’s Hospital, Changsha, China | (i) Serum MOG-IgG titre  (ii) CSF WCC | — | Low | Quantitative |
| *Included* | 48 | 6.57 ± 3.02* | 30 [62.5] | 11 [22.9] | ADEM (24), NMOSD (4), Autoimmune Encephalitis Overlap Syndrome (8), ON (5), Cranial neuritis (2), Brain (2), Meningitis (2), Demyelinating pseudotumor (1) | ≥12 |  |  |  |  |  |  |  |  |
| Yao, et al., 2022 ^62^  *Total* | 11 | 27 (16–32) | 4 [36.4] | 2 [18.2] | Brain (11) | 10 (3-23) | Fixed | ≥1:10 | Commercially available Euroimmun, Lubeck, Germany | Xiangya Hospital, Central South University, Changsha, China | (i) Serum MOG-IgG titre  (ii) CSF WCC  (iii) CSF protein | — | Medium | Quantitative |
| *Included* | 4 | 24.5 (18-31) | 1 [25.0] | 1 [25.0] | Brain (4) | 21 (12-23) |  |  |  |  |  |  |  |  |
| Bauer, et al., 2022 ^63^  *Total* | 40 | 23.7 (3.3-72.0)^ | 22 [55.0] | 16/32 [50.0] | NA | 12 (6-38) | Live | ≥1:160 | In-house CBA at Medical University of Innsbruck, Innsbruck, Austria | Medical University of Innsbruck, Innsbruck, Austria | (i) Serum cytokines and chemokines | (i) Serum cytokines and chemokines | Low | Qualitative |
| *Included* | 40 | 23.7 (3.3-72.0)^ | 22 [55.0] | 16/32 [50.0] | NA | 12 (6-38) |  |  |  |  |  |  |  |  |
| Rechtman, et al., 2024 ^64^  *Total* | 26 | 29.91 ± 15.62* | 18 [69.2] | NA | NA | NA | NA | NA | NA | Hadassah-Hebrew University Medical Center, Ein–Kerem, Hebrew University of Jerusalem. Jerusalem, Israel | (i) Serum thyroid function profile | — | Low | Qualitative |
| *Included* | 26 | 29.91 ± 15.62* | 18 [69.2] | NA | NA | NA |  |  |  |  |  |  |  |  |
| Nguyen, et al., 2024 ^65^  *Total* | 43 | (i) 10.7 (5.8-13.2)^$^  (ii) 5.0 (4.3-12.2)^$o^ | 20 [46.5] | 10 [23.3] | ADEM (14), ON (22), TM (3), Other (4) | (i) 13.7 (5.1-30.4)^$^  (ii) 52.1 (28.5-105.2)^$o^ | Live and fixed | NA | NA | University of Texas Southwestern Medical Center, Dallas | (i) Serum MOG-IgG titre  (ii) Serial serum MOG-IgG status  (iii) CSF WCC  (iv) CSF protein | (i) Serum MOG-IgG titre  (ii) CSF WCC  (iii) CSF protein | Medium | Quantitative |
| *Included* | 5 | 4 (3-6) | 2 [40.0] | 2 [40.0] | ADEM (5) | 86 (21-151) |  |  |  |  |  |  |  |  |
| ZhangBao, et al., 2023 ^66^  *Total* | 186 | 25 (2–65) | 100 [53.8] | 112 [60.2] | Onset attack: ON (94), TM (29), Brain (20), ADEM (19), Brainstem (5), Other brain syndrome (3), Mixed (16) | 51 (9–326) | Fixed | NA | NA | Shanghai Medical College, Fudan University, Shanghai, People’s Republic of China | (i) Serial serum MOG-IgG status | — | Medium | Quantitative |
| *Included* | 44 | NA | NA | 38 [86.4] | NA | 108.5 (60-326) |  |  |  |  |  |  |  |  |
| Montalvo, et al., 2022 ^67^  *Total* | 23 | 9 (2-47) | 12 [52.2] | 16 [69.6] | ADEM (2), Brain (3), ADEM+ON (3), ADEM+Brain (1), Brain+ON (2), Brain+TM (1), Brain+ON+TM (4), ADEM+Brain+ON (2), ADEM+ON+TM (3), ADEM+Brain+ON+TM (2) | NA | Live | NA | Referred CBA to Mayo Clinic, USA | Mayo Clinic, USA | (i) Serum MOG-IgG titre  (ii) Serial serum MOG-IgG status | (i) Serum MOG-IgG tire | Medium | Quantitative |
| *Included* | 2 | 13 (6-20) | 1 [50.0] | 2 [100.0] | ADEM+Brain (1), ADEM+Brain+ON (1) | 156 (108-204) |  |  |  |  |  |  |  |  |
| Aktas, et al., 2023 ^68^  *Total* | 7 | NA | NA | NA | NA | ≥24 | NA | NA | NA | Heinrich Heine University Düsseldorf, Düsseldorf, Germany | — | (i) Serum GFAP  (ii) Serum NfL  (iii) Serum tau  (iv) Serum UCHL1 | Low | Qualitative and quantitative |
| *Included* | 8 serum samples; unclear participants | NA | NA | NA | NA | ≥24 |  |  |  |  |  |  |  |  |
| Akaishi, et al., 2023 ^69^  *Total* | 26 | 43 (34.5–56)^$!^ | 18 [69.2] | NA | NA | NA | Live | NA | In-house CBA at Tohoku University School of Medicine, Sendai, Japan | Tohoku University School of Medicine, Sendai, Japan | — | (i) Peripheral blood count index ratios | Low | Qualitative |
| *Included* | 26 | 43 (34.5–56)^$!^ | 18 [69.2] | NA | NA | NA |  |  |  |  |  |  |  |  |
| Wang, et al., 2022 ^70^  *Total* | 4 | 21 (20–51) | 1 [25.0] | 2 [50.0] | Brain (3), Brainstem (1) | 14 (6-72) | NA | NA | NA | Affiliated Hospital Xingtai People’s Hospital, Hebei Medical University, Xingtai, China | (i) Serum MOG-IgG titre  (ii) Serial serum MOG-IgG status  (iii) CSF WCC  (iv) CSF protein  (v) CSF OCB | (i) Serum MOG-IgG titre  (ii) CSF WCC  (iii) CSF protein  (iv) CSF OCB | Medium | Quantitative |
| *Included* | 3 | 20 (20-22) | 0 [0.0] | 2 [66.7] | Brain (3) | 16 (12-72) |  |  |  |  |  |  |  |  |
| Wendel, et al., 2022 ^71^  *Total* | 116 | 7 (4–12)^$^ | 57 [49.1] | 44 [37.9] | Onset attack: ADEM (59), uON (21), bON (16), TM (6), NMOSD (8), Brain (6) | 43 (27-69)^$^ ;  all ≥24 | Live | ≥1:160 | In-house CBA at Medical University of Innsbruck, Innsbruck, Austria | Olga Hospital, Klinikum Stuttgart, Germany | (i) Serum MOG-IgG titre  (ii) Serial serum MOG-IgG status  (iii) CSF WCC  (iv) CSF OCB | (i) Serum MOG-IgG titre | Low | Quantitative |
| *Included* | 107 participants for CSF analysis and 70 participants for serum analysis; unclear overlap | NA | NA | 44 [40.4] | NA | ≥24 |  |  |  |  |  |  |  |  |
| Guzman, et al., 2023 ^72^  *Total* | 35 | 30 (1–64) | 25 [71.4] | 12 [34.3] | Onset attack: ON (12), TM (8), ADEM (5), Brain (4), ON+TM (3), Brainstem (1), Area Postrema Syndrome (1) | 24 (12–348)^p^ | Fixed | ≥1:10 | Commercially available Euroimmun, Lubeck, Germany | Pontifical Catholic University of Chile, Santiago, Chile | (i) Serial serum MOG-IgG status  (ii) CSF WCC | (i) CSF WCC | Medium | Quantitative |
| *Included* | 3 | NA | NA | 2 [66.7] | NA | 30 (12-36) |  |  |  |  |  |  |  |  |
| Gastaldi, et al., 2022 ^73^  *Total* | 102 | 17 (6-33)^$^ | 59 [57.8] | 44 [43.1] | uON (20), bON (18), TM (7), LETM (4), ADEM (28), NMOSD (16), Other (9) | 29 (3-320) | Live | ≥1:160 | In-house CBA at IRCCS Mondino Foundation, Pavia, Lombardia, Italy following protocol from Medical University of Innsbruck, Innsbruck, Austria | IRCCS Mondino Foundation, Pavia, Lombardia, Italy | (i) Serial serum MOG-IgG status | (i) Serum MOG-IgG titre | Medium | Quantitative |
| *Included* | 162 samples for MOG-IgG titre analysis and 80 participants for serial MOG-IgG status analysis; unclear overlap | NA | NA | 36 [45.0] | NA | 30.5 (14-320) |  |  |  |  |  |  |  |  |
| Wu, et al., 2023 ^74^  *Total* | 9 | 29 (15–57) | 4 [44.4] | 2 [22.2] | Brain (9) | 9 (2-36) | NA | ≥1:10 | Referred CBA to Omeng Weiyi Medical Laboratory, Hangzhou, China | The Affiliated Brain Hospital of Nanjing Medical University, Nanjing, China | (i) Serum MOG-IgG titre  (ii) CSF WCC  (iii) CSF protein  (iv) CSF OCB | (i) Serum MOG-IgG titre  (ii) CSF WCC  (iii) CSF protein  (iv) CSF OCB | Medium | Quantitative |
| *Included* | 4 | 23.5 (15-29) | 1 [25.0] | 1 [25.0] | Brain (4) | 25 (13-36) |  |  |  |  |  |  |  |  |
| Liu, et al., 2024 ^75^  *Total* | 15 | 29.20 ± 11.87* | 3 [20.0] | NA | NA | NA | Fixed | ≥1:10 | Commercially available Euroimmun, Lubeck, Germany | Fujian Medical University, Fujian, China | — | (i) Serum prolactin | Low | Qualitative |
| *Included* | 15 | 29.20 ± 11.87* | 3 [20.0] | NA | NA | NA |  |  |  |  |  |  |  |  |
| Zhou, et al., 2022 ^76^  *Total* | 19 | 6 ± 3*^ | 9 [47.4] | NA | ON (2), ADEM (15), Other (2) | NA | NA | NA | NA | Beijing Children’s Hospital, Capital Medical University, National Center for Children’s Health, Beijing, China | — | (i) Serum sTREM2 | Low | Qualitative |
| *Included* | 19 | 6 ± 3*^ | 9 [47.4] | NA | ON (2), ADEM (15), Other (2) | NA |  |  |  |  |  |  |  |  |
| Zhou, et al., 2022 ^77^  *Total* | 30 | 7.05 (2.50–12.75) | 17 [56.7] | 30 [100.0] | NMOSD (10), MDEM (3), ADEM+ON (2), RON (2), Unclassified (13) | 45 (19.9-105.9) | Fixed | ≥1:10 | Commercially available Euroimmun, Lubeck, Germany | Beijing Children’s Hospital, Capital Medical University, National Center for Children’s Health, Beijing, China | (i) Serum MOG-IgG titre | — | Low | Quantitative |
| *Included* | 30 | 7.05 (2.50–12.75) | 17 [56.7] | 30 [100.0] | NMOSD (10), MDEM (3), ADEM+ON (2), RON (2), Unclassified (13) | 45 (19.9-105.9) |  |  |  |  |  |  |  |  |
| Yang, et al., 2024 ^78^  *Total* | 6 | 31.5 (29–40)^ | 1 [16.7] | 2 [33.3] | Brain (6) | 37.5 (6-62) | NA | NA | NA | Tianjin Huanhu Hospital, Tianjin, People’s Republic of China | (i) Serum MOG-IgG titre  (ii) Serial serum MOG-IgG status  (iii) CSF WCC  (iv) CSF protein  (v) CSF OCB | (i) Serum MOG-IgG titre  (ii) CSF WCC  (iii) CSF protein  (iv) CSF OCB | Medium | Quantitative |
| *Included* | 2 | 32 (30-34)^ | 1 [50.0] | 0 [0] | Brain (2) | 47 (39-55) |  |  |  |  |  |  |  |  |
| Wang, et al., 2023 ^79^  *Total* | 2 | 1.75 (0.5-3) | 0 [0] | 2 [100.0] | Brain (2) | 42 (36-48) | Live | NA | NA | Hebei Children’s Hospital, Shijiazhuang, China | i) Serum MOG-IgG titre  (ii) Serial serum MOG-IgG status  (iii) CSF WCC  (iv) CSF protein  (v) CSF OCB | (i) Serum MOG-IgG titre  (ii) CSF WCC  (iii) CSF protein  (iv) CSF OCB | Low | Quantitative |
| *Included* | 2 | 1.75 (0.5-3) | 0 [0] | 2 [100.0] | Brain (2) | 42 (36-48) |  |  |  |  |  |  |  |  |
| Roy, et al., 2023 ^80^  *Total* | 10 | 9 (1-58) | 6 [60.0] | 7 [70.0] | ON (3), ADEM (3), ADEM+ON (3), ON+TM (1) | 33 (16-98) | Live | ≥1:20 | Referred CBA at Mayo Clinic, USA | Johns Hopkins University, Baltimore, MD, USA | (i) Serum MOG-IgG titre  (ii) Serial serum MOG-IgG status | (i) Serum MOG-IgG titre | Low | Quantitative |
| *Included* | 10 | 9 (1-58) | 6 [60.0] | 7 [70.0] | ON (3), ADEM (3), ADEM+ON (3), ON+TM (1) | 33 (16-98) |  |  |  |  |  |  |  |  |
| Lee, et al., 2023 ^81^  *Total* | 40 | 32 (22.5–44.5)^$^ | 11 [27.5] | 16 [40.0] | ADEM (12), Brain (21), ADEM+Brain (5), ADEM+ON (2) | 29.5 (18.5–36.5)^$^; all ≥12 | Live | MFI ≥2.60 | In-house CBA at Seoul National University Hospital, Seoul National University College of Medicine, Seoul, South Korea | Seoul National University Hospital, Seoul National University College of Medicine, Seoul, South Korea | (i) Serum MOG-IgG titre  (ii) Serial serum MOG-IgG status  (iii) CSF WCC  (iv) CSF protein  (v) CSF OCB | — | Low | Quantitative |
| *Included* | 40 | 32 (22.5–44.5)^$^ | 11 [27.5] | 16 [40.0] | ADEM (12), Brain (21), ADEM+Brain (5), ADEM+ON (2) | 29.5 (18.5–36.5)^$^; all ≥12 |  |  |  |  |  |  |  |  |
| Xu, et al., 2023 ^82^  *Total* | 35 | 30 (15–73) | 22 [62.9] | 14 [40.0] | Brainstem involvement (35) | 38 (4–64) | Live | ≥1:10 | In-house CBA at Xuanwu Hospital Neuroimmunology Laboratory | Xuanwu Hospital, Capital Medical University, Beijing, China | (i) Serum MOG-IgG titre  (ii) Serial serum MOG-IgG status  (iii) CSF OCB | (i) Serum MOG-IgG titre  (ii) CSF OCB | Low | Quantitative |
| *Included* | 22 | NA | NA | 13 [59.1] | Brainstem involvement (22) | 34 (13-58) |  |  |  |  |  |  |  |  |
| Lin, et al., 2023 ^83^  *Total* | 31 | 31.94 ± 18.03* | 13 [41.9] | 14 [45.2] | Overall phenotype not available; clinical symptoms at onset: ON (9), TM (9) | ≥12 | Fixed | ≥1:10 | Commercially available Euroimmun, Lubeck, Germany | Affiliated Nanjing Brain Hospital, Nanjing Medical University, Nanjing, Jiangsu, China | (i) Peripheral blood count index ratios | — | Low | Qualitative |
| *Included* | 31 | 31.94 ± 18.03* | 13 [41.9] | 14 [45.2] | Overall phenotype not available; clinical symptoms at onset: ON (9), TM (9) | ≥12 |  |  |  |  |  |  |  |  |
| Horellou, et al., 2023 ^84^  *Total* | 15 | (i) Monophasic: 6.8 ± 3.8*  (ii) Relapsing: 5.8 ± 3.5* | 9 [60.0] | 8 [53.3] | Overall phenotype not available; onset phenotype: ON (2), ADEM (5), Brain (4), NMOSD (4) | (i) Monophasic: 4.5 ± 2.7*^&^  (ii) Relapsing: 8.6 ± 4.8*^&^ | Live | ≥1:160 | In-house CBA at Center for Immunology of Viral, Auto-Immune, Hematological and Bacterial diseases (IMVA-HB/IDMIT), Université Paris- Saclay, CEA, INSERM, Le Kremlin Bicêtre, France | Center for Immunology of Viral, Auto-Immune, Hematological and Bacterial diseases (IMVA-HB/IDMIT), Université Paris- Saclay, CEA, INSERM, Le Kremlin Bicêtre, France | — | (i) Serum NfL | Low | Qualitative and quantitative |
| *Included* | 15 | (i) Monophasic: 6.8 ± 3.8*  (ii) Relapsing: 5.8 ± 3.5* | 9 [60.0] | 8 [53.3] | Overall phenotype not available; onset phenotype: ON (2), ADEM (5), Brain (4), NMOSD (4) | (i) Monophasic: 4.5 ± 2.7*  (ii) Relapsing: 8.6 ± 4.8* |  |  |  |  |  |  |  |  |
| Hacohen, et al., 2014 ^85^  *Total* | 7 | 12 (3-15) | 5 [71.4] | 2 [28.6] | ADEM (2), ON (2), TM (1), MS (2) | 12 | Live | ≥1:20 | In-house CBA at University of Oxford, UK | John Radcliffe Hospital, Oxford University Hospital, Oxford, UK | (i) Serial serum MOG-IgG status  (ii) CSF WCC  (iii) CSF OCB | — | Low | Quantitative |
| *Included* | 4 | 6 (3-12) | 2 [50.0] | 0 [0] | ADEM (2), ON (2) | 12 |  |  |  |  |  |  |  |  |
| Salunkhe, et al., 2023 ^86^  *Total* | 64 | (i) Encephalitis group: 14.5 (11.75–18)^  (ii) Non-encephalitis group: 28 (19.75–42)^ | 33 [51.6] | 36 [56.3] | Encephalitis group: Brain (4), Brain+Brainstem/Cerebellar (2), Brain+ON (4), Brain+TM (3), Brain+ON+TM (3)  Non-encephalitis group: NA | NA | Fixed | NA | NA | All India Institute of Medical Sciences, New Delhi, India | (i) CSF WCC  (ii) CSF protein | — | Medium | Quantitative |
| *Included* | 3 | 13 (11-15) | 1 [33.3] | 2 [66.7] | Brain+ON (1), Brain+ON+TM (2) | 36 (12-36) |  |  |  |  |  |  |  |  |
| Huang, et al., 2024 ^87^  *Total* | 110 | (i) Paediatric onset: 6 (3-13)  (ii) Adult onset: 26.5 (14-49)  (iii) Late onset: 58 (50-78) | 59 [53.6] | 44 [40.0] | MS (12), NMOSD (24), ADEM (17), ON (28), TM (6), IIDDs (23) | ≥12 | Fixed | ≥1:10 | NA | The Third Affiliated Hospital of Sun Yat-sen University, Guangzhou, China | (i) Serum homocysteine | (i) Serum MOG-IgG titre | Low | Qualitative and quantitative |
| *Included* | (i) Homocysteine level: 20  (ii) MOG-IgG titre: 131 samples; unclear participants | NA | NA | NA | NA | ≥12 |  |  |  |  |  |  |  |  |
| Samadzadeh, et al., 2023 ^88^  *Total* | 22 | 48.6 (33.3-56.5)^$!^ | 8 [36.3] | NA | Overall phenotype not available; Most recent attack phenotype prior to sampling: ON (7), TM (3), Brain (4), Unknown (8) | NA | Live | NA | In-house CBA at University of Southern Denmark, Odense, Denmark | University of Southern Denmark, Odense, Denmark | — | (i) CSF MFAP4 | Low | Qualitative |
| *Included* | 22 | 48.6 (33.3-56.5)^$!^ | 8 [36.3] | NA | Overall phenotype not available; Most recent attack phenotype prior to sampling: ON (7), TM (3), Brain (4), Unknown (8) | NA |  |  |  |  |  |  |  |  |
| Liyanage, et al., 2024 ^89^  *Total* | 202 | 39.89 (30.85–51.24)^$^ | 121 [59.9] | 137 [67.8] | uON (62), bON (31), ON NOS (2), uON+bON (24), ON+TM (23), TM (25), Brain (9), Mixed (26) | 48 (26.4-86.4)^$^; all ≥12 | Live | MFI > mean + 3 SD of control | In-house CBA at Kids Neuroscience Centre, Kids Research at the Children’s Hospital at Westmead, Australia | Kids Neuroscience Centre, Kids Research at the Children’s Hospital at Westmead, Australia | (i) Serum MOG epitope recognition pattern | — | Low | Qualitative |
| *Included* | 202 | 39.89 (30.85–51.24)^$^ | 121 [59.9] | 137 [67.8] | uON (62), bON (31), ON NOS (2), uON+bON (24), ON+TM (23), TM (25), Brain (9), Mixed (26) | 48 (26.4-86.4)^$^; all ≥12 |  |  |  |  |  |  |  |  |
| Wang, et al., 2023 ^90^  *Total* | 11 | 20^^q^ | 6 [54.6] | NA | ON (1), TM (6), ON+TM (2), Brainstem (2) | NA | Fixed | NA | Commercially available Euroimmun, Lubeck, Germany | The Sixth People’s Hospital, Shanghai Jiao Tong University, Shanghai, China | — | (i) Serum cytokines | Low | Qualitative |
| *Included* | 11 | 20^^q^ | 6 [54.6] | NA | ON (1), TM (6), ON+TM (2), Brainstem (2) | NA |  |  |  |  |  |  |  |  |
| Yandamuri, et al., 2023 ^91^  *Total* | 25 | Number in Age Range: 0-9 (1), 10-19 (2), 20-29 (5), 30-39 (5), 40-49 (3), 50-59 (8), Unknown (1)^ | 17 [68.0] | NA | NA | NA | Live | NA | In-house CBA at Yale School of Medicine, New Haven, Connecticut, USA | Yale School of Medicine, New Haven, Connecticut, USA | — | (i) Serum MOG-IgG effector functions (CDC and ADCP) | Low | Qualitative |
| *Included* | 25 | Number in Age Range: 0-9 (1), 10-19 (2), 20-29 (5), 30-39 (5), 40-49 (3), 50-59 (8), Unknown (1)^ | 17 [68.0] | NA | NA | NA |  |  |  |  |  |  |  |  |
| Nguyen, et al., 2023 ^92^  *Total* | 67 | (i) 9.99 ± 4.15*^r^  (ii) 6.06 ± 3.56*^r^ | 36 [53.7] | NA | Overall phenotype not available; onset phenotype: ADEM (23), ON (27), TM (5), Other (12) | (i) 45.09 ± 41.05*^r^  (ii) 73.12 ± 55.55*^r^ | Live | NA | Referred CBA at Mayo Clinic, USA and University of Oxford, Oxford, UK | University of Texas Southwestern Medical Center, Dallas, TX, USA | (i) Serum MOG-IgG titre  (ii) Serial serum MOG-IgG status | (i) Serum MOG-IgG titre | Low | Quantitative |
| *Included* | 10 | 7 (3-14)^ | 8 [80.0] | 5 [50.0] | ADEM (3), ON (1), ADEM+Brain (1), ADEM+Brainstem/Cerebellar (2), ADEM+ON (1), Brainstem/Cerebellar+ON (1), ON+Other (1) | 57 (16-192) |  |  |  |  |  |  |  |  |
| Wendel, et al., 2022 ^93^  *Total* | 19 | 5 (4-7)^$^ | 6 [31.6] | 0 [0] | ADEM (14), ON (3), LETM (1), NMOSD (1) | 24 (24-36)^$^ | Live | ≥1:160 | Referred CBA at Innsbruck Medical University, Innsbruck, Austria | Olgahospital, Klinikum Stuttgart, Stuttgart, Germany | — | (i) Serum NfL | Low | Qualitative and quantitative |
| *Included* | 19 | 5 (4-7)^$^ | 6 [31.6] | 0 [0] | ADEM (14), ON (3), LETM (1), NMOSD (1) | 24 (24-36)^$^ |  |  |  |  |  |  |  |  |
| Zhang, et al., 2022 ^94^  *Total* | 12 | 8 (6–14.5) | 3 [37.5] | 4 [33.3] | Overall phenotype NA; onset phenotype: Brain/Brainstem (5), ON+TM (4), Brain/Brainstem+ON (3) | 12 | Fixed | NA | NA | Jinan Central Hospital, Cheeloo College of Medicine, Shandong University, Jinan, China | (i) CSF OCB | — | Low | Quantitative |
| *Included* | 12 | 8 (6–14.5) | 3 [37.5] | 4 [33.3] | Overall phenotype NA; onset phenotype: Brain/Brainstem (5), ON+TM (4), Brain/Brainstem+ON (3) | 12 |  |  |  |  |  |  |  |  |
| Aubart, et al., 2022 ^95^  *Total* | 3 | 4 (1-10) | 1 [33.3] | 0 [0] | ADEM (2), ADEM+ON (1) | 12-18^e^ | Fixed | NA | Commercially available Euroimmun, Lubeck, Germany | University of Paris Cite, Paris, France | (i) Serial serum MOG-IgG status  (ii) CSF WCC  (iii) CSF protein  (iv) CSF OCB | (i) CSF WCC  (ii) CSF protein  (iii) CSF OCB | Low | Quantitative |
| *Included* | 3 | 4 (1-10) | 1 [33.3] | 0 [0] | ADEM (2), ADEM+ON (1) | 12-18^e^ |  |  |  |  |  |  |  |  |
| Tzanetakos, et al., 2022 ^96^  *Total* | 11 | 37 (3-75)^!^ | 8 [72.7] | 4 [36.4] | uON (3), bON (3), Brainstem (1), NMO (1), Brain+uON (1), ADEM (1), TM (1) | 18 (1-49) | Live | ≥1:20 | In-house CBA at Eginition Hospital, National and Kapodistrian University of Athens, Athens, Greece | Eginition Hospital, National and Kapodistrian University of Athens, Athens, Greece | (i) Serum MOG-IgG titre  (ii) CSF OCB | — | Medium | Quantitative |
| *Included* | 7 | 56 (19-75)! | 5 [71.4] | 3 [42.9] | uON (3), bON (2), Brainstem (1), ADEM (1) | 20 (13-49) |  |  |  |  |  |  |  |  |
| Kim, et al., 2022 ^97^  *Total* | 6 | 27.2 ± 18.2*^ | 5 [83.3] | 3 [50.0] | ON (6) | 25.2 (12-48)^%^ | Live | ≥2.5 | In-house CBA at Kyungpook National University, Daegu, Korea | Kyungpook National University, Daegu, Korea | (i) Serum LCN2 | — | Low | Qualitative |
| *Included* | 6 | 27.2 ± 18.2*^ | 5 [83.3] | 3 [50.0] | ON (6) | 25.2 (12-48)^%^ |  |  |  |  |  |  |  |  |
| Nguyen, et al., 2024 ^98^  *Total* | 87 | 8.3 (4.8-12.2)^$^ | 44 [50.6] | 27/61 [44.3] | Overall phenotype NA; onset phenotype: ADEM (32), ON (33), TM (6), Other (16) | 29.5 (8.7–55.0)^$^ (85/87) | Live and unknown | ≥1:20 (live) | Referred CBA at Mayo Clinic, USA, University of Oxford, Oxford, UK, and unknown | University of Texas Southwestern Medical Center, Dallas, TX, USA | (i) Serum MOG-IgG titre  (ii) Serial serum MOG-IgG status  (iii) CSF WCC  (iv) CSF protein  (v) CSF OCB | (i) Serum MOG-IgG titre  (ii) CSF WCC  (iii) CSF protein  (iv) CSF OCB | Medium | Quantitative |
| *Included* | 52 | NA | NA | 20 [38.5] | NA | NA |  |  |  |  |  |  |  |  |
| Fadda, et al., 2022 ^99^  *Total* | 12 | 9.04 (6.50-10.36)^$^ | 8 [66.7] | 7 [58.3] | ON (4), TM (1), ON+TM (4), ADEM+TM (1), ADEM+ON+Other (1), Other (1) | 77.5 (12-132) | Live | ≥1 | In-house CBA at Perelman School of Medicine, University of Pennsylvania, Philadelphia, USA | Perelman School of Medicine, University of Pennsylvania, Philadelphia, USA | (i) Serial serum MOG-IgG status | (i) Serum MOG-IgG titre | Low | Quantitative |
| *Included* | 11 | NA | NA | 7 [63.6] | ON (3), TM (1), ON+TM (4), ADEM+TM (1), ADEM+ON+Other (1), Other (1) | 81 (12-132) |  |  |  |  |  |  |  |  |
| Vosoughi, et al., 2023 ^100^  *Total* | 4 | 37 (18-45) | 2 [50.0] | 2 [50.0] | uON (3), uON+TM+LETM (1) | 54 (12-108) | Live and fixed | NA | NA | Max Rady College of Medicine, University of Manitoba, Winnipeg, Manitoba, Canada | (i) Serum MOG-IgG titre  (ii) Serial serum MOG-IgG status | (i) Serum MOG-IgG titre | Low | Quantitative |
| *Included* | 4 | 37 (18-45) | 2 [50.0] | 2 [50.0] | uON (3), uON+TM+LETM (1) | 54 (12-108) |  |  |  |  |  |  |  |  |
| Jiang, et al., 2023 ^101^  *Total* | 4 | 2.5 (2-5) | 2 [50.0] | 0 [0] | Brain (1), ADEM (3) | 13.5 (11-47) | NA | NA | NA | Hospital of Chongqing Medical University, National Clinical Research Center for Child Health and Disorders, Ministry of Education Key Laboratory of Child Development and Disorders, China | (i) CSF WCC  (ii) CSF protein  (iii) CSF OCB | (i) CSF WCC  (ii) CSF protein  (iii) CSF OCB | Medium | Quantitative |
| *Included* | 4 | 2.5 (2-5) | 2 [50.0] | 0 [0] | Brain (1), ADEM (3) | 13.5 (11-47) |  |  |  |  |  |  |  |  |
| Zeng, et al., 2023 ^102^  *Total* | 15 | 29 (4-65) | 7 [46.7] | 6 [40.0] | ON (7), TM (1), Brain (2), Brainstem (1), Brain+Brainstem/Cerebellar (1), Brain+ON (1), Brain+Brainstem/Cerebellar+ON (1) | 33 (24-64) | Fixed | ≥1:10 | Referred CBA at Guangzhou Jinyu Medical Laboratory, Guangzhou, China | Liuzhou People’s Hospital, Liuzhou, China | (i) Serum MOG-IgG titre  (ii) Serial serum MOG-IgG status  (iii) CSF WCC  (iv) CSF protein | (i) Serum MOG-IgG titre | Medium | Quantitative |
| *Included* | 15 | 29 (4-65) | 7 [46.7] | 6 [40.0] | ON (7), TM (1), Brain (2), Brainstem (1), Brain+Brainstem/Cerebellar (1), Brain+ON (1), Brain+Brainstem/Cerebellar+ON (1) | 33 (24-64) |  |  |  |  |  |  |  |  |
| Baek, et al., 2023 ^103^  *Total* | 39 | 37.4 ± 12* | 20 [51.3] | NA | NA | NA | NA | NA | NA | Sungkyunkwan University School of Medicine, Seoul, South Korea | — | (i) Peripheral blood count index ratios | Low | Qualitative |
| *Included* | 39 | 37.4 ± 12* | 20 [51.3] | NA | NA | NA |  |  |  |  |  |  |  |  |
| Singh, et al., 2022 ^104^  *Total* | 2 | 10 (5-15) | 2 [100] | 2 [100] | uON+Brain (2) | 126 (36-216) | Live | NA | NA | Texas Tech University Health Sciences Center, El Paso, USA | (i) Serum MOG-IgG titre  (ii) Serial serum MOG-IgG status  (iii) CSF OCB | (i) Serum MOG-IgG titre  (ii) CSF OCB | Low | Quantitative |
| *Included* | 2 | 10 (5-15) | 2 [100] | 2 [100] | uON+Brain (2) | 126 (36-216) |  |  |  |  |  |  |  |  |
| Wang, et al., 2023 ^105^  *Total* | 25 | 27 (15-35)^$^^ | 10 [40.0] | 8 [32.0] | Overall phenotype NA; regions affected at NOS time: Brain (18), Brainstem (6), ON (4), ADEM (3), Cerebellar (2) | 12 | Fixed | NA | Commercially available Euroimmun, Lubeck, Germany | Qilu Hospital, Cheeloo College of Medicine, Shandong University, Jinan, Shandong, China | (i) Peripheral blood count index ratios | — | Low | Qualitative |
| *Included* | 25 | 27 (15-35)^$^^ | 10 [40.0] | 8 [32.0] | Overall phenotype NA; regions affected at NOS time: Brain (18), Brainstem (6), ON (4), ADEM (3), Cerebellar (2) | 12 |  |  |  |  |  |  |  |  |
| Song, et al., 2022 ^106^  *Total* | 18 | 9.5 (3-13)^ | 12 [66.7] | 3 [16.7] | Brain (13), Cerebellar (1), Brain+Brainstem (1), Brain+Cerebellar (2), Brain+Brainstem+Cerebellar (1) | 17 (8-39) | Fixed | NA | NA | Children’s Hospital of Chongqing Medical University, National Clinical Research Center for Child Health and Disorders, Ministry of Education Key Laboratory of Child Development and Disorders, Chongqing Key Laboratory of Pediatrics, Chongqing, China | (i) Serial serum MOG-IgG status  (ii) CSF WCC  (iii) CSF protein | (i) CSF WCC  (ii) CSF protein | Medium | Quantitative |
| *Included* | 17 | 9 (3-13)^ | 12 [70.6] | 3 [17.7] | Brain (12), Cerebellar (1), Brain+Brainstem (1), Brain+Cerebellar (2), Brain+Brainstem+Cerebellar (1) | 17 (13-39) |  |  |  |  |  |  |  |  |
| Masuda, et al., 2023 ^107^  *Total* | 18 | 45 (26.25-63.75)^$!^ | 12 [66.7] | NA | NA | NA | NA | NA | NA | Graduate School of Medicine, Chiba University, Inohana, Chuo-Ku, Chiba-Shi, Japan | — | (i) Serum and CSF cytokines | Low | Qualitative |
| *Included* | 18 | 45 (26.25-63.75)^$!^ | 12 [66.7] | NA | NA | NA |  |  |  |  |  |  |  |  |

NA, not available. Phenotypes were directly reported as they appeared in the original manuscript

^$^Median (IQR)

^%^Mean (range)

*Mean ± SD

^Age not otherwise specified

^#^Original study reports phenotypes for n=13; discrepancy with total participants n=12

^@^Original study defines as “disease duration at last follow-up”

^!^Age at sampling

^~^Original study reported characteristic by following subgroups: (i) paediatric persistent MOG-IgG seropositivity, (ii) paediatric transient MOG-IgG seropositivity, (iii) adult persistent MOG-IgG seropositivity, and (iv) adult transient MOG-IgG seropositivity

^a^Phenotype partially available

^b^Original study reported characteristic by following subgroups: (i) persistent MOG-IgG seropositivity and (ii) transient MOG-IgG seropositivity

^c^Original study reported phenotypes for n=97; discrepancy with total participants n=84

^d^Original study reported characteristic by following subgroups: (i) attack and (ii) remission

^e^Range only

^f^All included participants had the same follow-up duration

^g^Original study reported characteristic by following subgroups: (i) MOG-IgG titre 1:160-1:640 and (ii) MOG-IgG titre ≥1:1280

^h^Age partially available

^i^Sex partially available

^j^Disease course partially available

^k^Only age decade specified, therefore, estimated as mid-decade

^l^Original study reported characteristic by following subgroups: (i) isolated recurrent ON (rON only) and (ii) recurrent ON with subsequent central nervous system demyelinating attack beyond the optic nerve (rON-plus)

^m^Follow-up duration for participants with MOGAD, AQP4-IgG positive NMOSD, and double seronegative NMOSD

^n^Original study reported characteristic by following subgroups: (i) paediatric monophasic, (ii) paediatric relapsing, (iii) adult monophasic, (iv) adult relapsing

^o^Original study reported characteristic by following subgroups: lumbar puncture opening pressure (i) ≤28cm H_2_O and (ii) >28cm H_2_O

^p^Unclear if median (IQR) or median (range) from original study

^q^Mean only; no SD reported

^r^Original study reported characteristic by following subgroups: (i) no subclinical disease activity on OCT and (ii) subclinical disease activity on OCT

**Online supplemental table 5: Comparison of follow-up duration between included monophasic and relapsing participants as reported by 48 studies**. Statistical significance assessed with Mann-Whitney U Test.

| **Source** | **Monophasic** | | **Relapsing** | | **Follow-up Data Format** | **P-value** |
| --- | --- | --- | --- | --- | --- | --- |
|  | **Participants (n)** | **Follow-up Duration, Median (range) (months)** | **Participants (n)** | **Follow-up Duration, Median (range) (months)** |  |  |
| Wendel, et al., 2020 ^5^ | 6 | 31.5 (12-36) | 3 | 36 (18-141) | IPD | 0.590 |
| Hyun, et al., 2017 ^7^ | 4 | 39 (23-113) | 15 | 65 (23-200) | IPD | 0.368 |
| Höftberger, et al., 2015 ^8^ | 5 | 43 (18-127) | 10 | 108 (17-415) | IPD | 0.371 |
| Horellou, et al., 2021 ^9^ | 5 | 22.8 ± 26.4* | 7 | 58.8 ± 26.4* | AD | 0.106 |
| Jarius, et al., 2020 ^11^ | 42 | 21 (0-65)^ | 38 | 63 (1-229)^ | AD | **<0.0001** |
| Ikeda,et al., 2019 ^14^ | 1 | 19^&^ | 3 | 46 (18-87) | IPD | 1 |
| Jurynczyk, et al., 2017 ^17^ | 27 | 20 (12-131) | 29 | 50 (12-438) | IPD | **<0.0001** |
| Hino-Fukuyo, et al., 2019 ^18^ | 2 | 140 (68-212) | 3 | 150 (87-322) | IPD | 0.800 |
| Serin, et al., 2021 ^19^ | 5 | 25 (24-34) | 4 | 41 (28-196) | IPD | 0.174 |
| Oliveira, et al., 2019 ^22^ | 8 | 49 (34-77)^$^ | 23 | 80 (38-105)^$^ | AD | 0.133 |
| de Mol, et al., 2020 ^25^ | 12 | 23 (12-88) | 8 | 41 (24-246) | IPD | 0.141 |
| Rostasy, et al., 2012 ^26^ | 2 | 37.5 (35-40) | 11 | 31 (19-74) | IPD | 0.923 |
| Han, et al., 2022 ^31^ | 5 | 19 (12-54) | 1 | 17^&^ | IPD | 1 |
| Alshamrani, et al., 2020 ^35^ | 1 | 13^&^ | 4 | 126 (48-132) | IPD | 0.277 |
| Lui, et al., 2021 ^37^ | 14 | 39 (15-150) | 9 | 100 (18-300) | IPD | **0.034** |
| Hennes, et al., 2017 ^39^ | 40 | 24^#^ | 25 | 24^#^ | AD | — |
| Tea, et al., 2019 ^40^ | 8 | 31.5 (17-77) | 8 | 34 (18-99) | IPD | 0.792 |
| Senanayake, et al., 2019 ^41^ | 4 | 12 (12-24) | 13 | 84 (36-204) | AD | **0.010** |
| Wegener-Panzer, et al., 2020 ^42^ | 2 | 18 (12-24) | 4 | 45 (24-48) | IPD | 0.153 |
| Baumann, et al., 2015 ^45^ | 10 | 27.5 (14-81) | 4 | 41.5 (30-67) | IPD | 0.357 |
| Armangue, et al., 2020 ^48^ | 53 | 24 (12-24) | 11 | 24^#^ | IPD | 0.535 |
| Nakajima, et al., 2015 ^49^ | 4 | 27.5 (15-53) | 3 | 30 (22-52) | IPD | 1 |
| Mao, et al., 2019 ^50^ | 10 | 13.5 (12-20) | 10 | 30.5 (12-63) | IPD | **0.013** |
| Zhang, et al., 2021 ^52^ | 25 | 33 (14-57) | 6 | 42.5 (16-63) | AD | 0.339 |
| Huda, et al., 2021 ^54^ | 34 | 35 (12-82) | 41 | 107 (12-400) | IPD | **<0.0001** |
| Nosadini, et al., 2023 ^60^ | 33 | 29 (12-122) | 18 | 51.5 (18-130) | AD | **0.032** |
| Yao, et al., 2022 ^62^ | 3 | 17 (12-23) | 1 | 20^&^ | IPD | 1 |
| Nguyen, et al., 2024 ^65^ | 3 | 52 (21-86) | 2 | 132.5 (114-151) | IPD | 0.200 |
| ZhangBao, et al., 2023 ^66^ | 6 | 61 (60-66) | 38 | 120 (66-326) | IPD | **<0.001** |
| Wang, et al., 2022 ^70^ | 1 | 16^&^ | 2 | 42 (12-72) | IPD | 1 |
| Wendel, et al., 2022 ^71^ | 65 | 36 (24-60)^$^ | 44 | 60 (36-84)^$^ | AD | **0.008** |
| Guzman, et al., 2023 ^72^ | 1 | 12^&^ | 2 | 33 (30-36) | IPD | 0.667 |
| Gastaldi, et al., 2022 ^73^ | 44 | 28.5 (14-142) | 36 | 40 (14-320) | IPD | **0.003** |
| Wu, et al., 2023 ^74^ | 3 | 24 (13-36) | 1 | 26^&^ | IPD | 1 |
| Roy, et al., 2023 ^80^ | 3 | 34 (30-37) | 7 | 32 (16-98) | IPD | 1 |
| Xu, et al., 2023 ^82^ | 9 | 32 (13-48) | 13 | 35 (13-58) | IPD | 0.547 |
| Horellou, et al., 2023 ^84^ | 7 | 54 ± 32.4* | 8 | 103.2 ± 57.6* | AD | 0.152 |
| Salunkhe, et al., 2023 ^86^ | 1 | 36^&^ | 2 | 24 (12-36) | IPD | 1 |
| Liyanage, et al., 2024 ^89^ | 65 | 33.5 (19.8-50.5)^$^ | 137 | 64 (28.4-115.2)^$^ | AD | **0.003** |
| Nguyen, et al., 2023 ^92^ | 5 | 48 (18-90) | 5 | 66 (16-192) | IPD | 1 |
| Zhang, et al., 2022 ^94^ | 8 | 12^#^ | 4 | 12^#^ | AD | — |
| Tzanetakos, et al., 2022 ^96^ | 4 | 22.5 (19-33) | 3 | 18 (18-49) | IPD | 0.629 |
| Nguyen, et al., 2024 ^98^ | 34 | 35.8 (26.4-49.5)^$^ | 27 | 71.9 (33.1-110.9)^$^ | AD | **0.017** |
| Fadda, et al., 2022 ^99^ | 4 | 76.5 (38-90) | 7 | 84 (12-132) | IPD | 0.649 |
| Vosoughi, et al., 2023 ^100^ | 2 | 60 (12-108 months) | 2 | 54 (36-72) | IPD | 1 |
| Zeng, et al., 2023 ^102^ | 9 | 36 (24-48) | 6 | 31.5 (26-64) | IPD | 0.814 |
| Wang, et al., 2023 ^105^ | 17 | 12^#^ | 8 | 12^#^ | AD | — |
| Song, et al., 2022 ^106^ | 14 | 17 (13-37) | 3 | 35 (16-39) | IPD | 0.229 |

*Mean ± SD

^Original study defined as “disease duration at last follow-up”

^&^Single participant follow-up duration

^$^Median (IQR)

^#^All included participants had the same follow-up duration

IPD=individual participant data, AD=aggregate data

**Online supplemental table 6: Investigating the association of serial serum MOG-IgG measurement at various sample collection intervals and relapsing disease course**

| **Based on ≥2 serum MOG-IgG results ≥3 months apart** | | | | | | | | | |
| --- | --- | --- | --- | --- | --- | --- | --- | --- | --- |
| Predictor of Relapsing Disease Course | | Studies (n) | Total Participants (n) | Monophasic Participants (n) | Relapsing Participants (n) | OR (95% CI) of Relapsing Disease Course | Univariable Random-Effects Meta-Analysis P-value | I^2^ | Egger’s Test P-value |
| Serial Serum MOG-IgG Status | |  | | | | | | | |
|  | Transient seropositivity | 48 | 777 | 178 | 72 | 2.7 (1.8–4.0) | <0.0001 | 0% | 0.28 |
|  | Persistent seropositivity |  |  | 245 | 282 |  |  |  |  |
| **Based on ≥2 serum MOG-IgG results ≥6 months apart** | | | | | | | | | |
| Predictor of Relapsing Disease Course | | Studies (n) | Total Participants (n) | Monophasic Participants (n) | Relapsing Participants (n) | OR (95% CI) of Relapsing Disease Course | Univariable Random-Effects Meta-Analysis P-value | I^2^ | Egger’s Test P-value |
| Serial Serum MOG-IgG Status | |  | | | | | | | |
|  | Transient seropositivity | 44 | 671 | 150 | 59 | 2.3 (1.5–3.4) | <0.0001 | 0% | 0.26 |
|  | Persistent seropositivity |  |  | 211 | 251 |  |  |  |  |
| **Based on ≥2 serum MOG-IgG results ≥12 months apart** | | | | | | | | | |
| Predictor of Relapsing Disease Course | | Studies (n) | Total Participants (n) | Monophasic Participants (n) | Relapsing Participants (n) | OR (95% CI) of Relapsing Disease Course | Univariable Random-Effects Meta-Analysis P-value | I^2^ | Egger’s Test P-value |
| Serial Serum MOG-IgG Status | |  | | | | | | | |
|  | Transient seropositivity | 41 | 567 | 147 | 43 | 2.8 (1.8–4. 5) | <0.0001 | 0% | 0.84 |
|  | Persistent seropositivity |  |  | 169 | 208 |  |  |  |  |

**Online supplemental table 7: Investigating the multivariable association of serial serum MOG-IgG measurement at various sample collection intervals and relapsing disease course alongside participant-level demographics.**

| **When persistent seropositivity is defined as ≥2 positive measurements ≥3 months apart** | | | | | | |
| --- | --- | --- | --- | --- | --- | --- |
| **Predictor of Relapsing Disease Course*** | | **Total Participants (n)** | **Monophasic Participants (n)** | **Relapsing Participants (n)** | **OR (95% CI) of Relapsing Disease Course** | **P value**^ |
| Serial MOG-IgG status |  |  |  |  |  |  |
|  | Transient seropositivity | 32 | 23 | 9 | 1.00 (reference) | **0.0059** |
|  | Persistent seropositivity | 96 | 18 | 78 | 14.56 (2.17-97.89) |  |
| Sex |  |  |  |  |  |  |
|  | Male | 59 | 25 | 34 | 1.00 (reference) | 0.9255 |
|  | Female | 69 | 16 | 53 | 1.07 (0.27-4.23) |  |
| Age |  |  |  |  |  |  |
|  | Paediatric (<18) | 77 | 27 | 50 | 1.00 (reference) | 0.7123 |
|  | Adult (≥18) | 51 | 14 | 37 | 1.51 (0.17-13.54) |  |
| Phenotype |  |  |  |  |  |  |
|  | Brain/Brainstem | 23 | 7 | 16 | 1.00 (reference) | — |
|  | ADEM | 26 | 18 | 8 | 0.05 (0.003-0.84) | **0.0373** |
|  | ON | 20 | 8 | 12 | 0.99 (0.07-13.66) | 0.9957 |
|  | TM | 5 | 3 | 2 | 0.20 (0.01-6.43) | 0.3595 |
|  | ADEM+ON | 16 | 1 | 15 | 5.58 (0.15-205.13) | 0.3497 |
|  | ON+TM | 10 | 2 | 8 | 6.67 (0.12-360.21) | 0.3512 |
|  | Mixed | 28 | 2 | 26 | 10.87 (0.61-193.27) | 0.1042 |
| ^Analysis was with a one-stage IPD meta-analysis with multivariable logistic mixed-effects regression. There was substantial heterogeneity (I^2^=76.93%).  *Minimum subgroup sample size criterion (n ≥5); phenotype ‘Other’ was excluded due to insufficient participant numbers (n=3) | | | | | | |
| **When persistent seropositivity is defined as ≥2 positive measurements ≥6 months apart** | | | | | | |
| **Predictor of Relapsing Disease Course*** | | **Total Participants (n)** | **Monophasic Participants (n)** | **Relapsing Participants (n)** | **OR (95% CI) of Relapsing Disease Course** | **P value**^ |
| Serial MOG-IgG status |  |  |  |  |  |  |
|  | Transient seropositivity | 24 | 16 | 8 | 1.00 (reference) | 0.1428 |
|  | Persistent seropositivity | 86 | 17 | 69 | 5.09 (0.58–44.86) |  |
| Sex |  |  |  |  |  |  |
|  | Male | 50 | 20 | 30 | 1.00 (reference) | 0.5194 |
|  | Female | 60 | 13 | 47 | 1.65 (0.36–7.60) |  |
| Age |  |  |  |  |  |  |
|  | Paediatric (<18) | 64 | 20 | 44 | 1.00 (reference) | 0.7347 |
|  | Adult (≥18) | 46 | 13 | 33 | 1.61 (0.10–24.81) |  |
| Phenotype |  |  |  |  |  |  |
|  | Brain/Brainstem | 22 | 7 | 15 | 1.00 (reference) | — |
|  | ADEM | 19 | 12 | 7 | 0.06 (0.002–2.10) | 0.1220 |
|  | ON | 16 | 6 | 10 | 1.89 (0.08–46.60) | 0.6960 |
|  | TM | 5 | 3 | 2 | 0.24 (0.01–12.12) | 0.4746 |
|  | ADEM+ON | 16 | 1 | 15 | 10.45 (0.15–726.97) | 0.2783 |
|  | ON+TM | 10 | 2 | 8 | 8.36 (0.13–543.20) | 0.3189 |
|  | Mixed | 22 | 2 | 20 | 8.64 (0.38–197.78) | 0.1770 |
| ^Analysis was with a one-stage IPD meta-analysis with multivariable logistic mixed-effects regression. There was substantial heterogeneity (I^2^=94.1%).  *Minimum subgroup sample size criterion (n ≥5); phenotype ‘Other’ was excluded due to insufficient participant numbers (n=2) | | | | | | |
| **When persistent seropositivity is defined as ≥2 positive measurements ≥12 months apart** | | | | | | |
| **Predictor of Relapsing Disease Course*** | | **Total Participants (n)** | **Monophasic Participants (n)** | **Relapsing Participants (n)** | **OR (95% CI) of Relapsing Disease Course** | **P value**^ |
| Serial MOG-IgG status |  |  |  |  |  |  |
|  | Transient seropositivity | 21 | 13 | 8 | 1.00 (reference) | 0.1030 |
|  | Persistent seropositivity | 75 | 15 | 60 | 5.79 (0.70–47.78) |  |
| Sex |  |  |  |  |  |  |
|  | Male | 46 | 18 | 28 | 1.00 (reference) | 0.7766 |
|  | Female | 50 | 10 | 40 | 1.26 (0.26–6.13) |  |
| Age |  |  |  |  |  |  |
|  | Paediatric (<18) | 53 | 16 | 37 | 1.00 (reference) | 0.9459 |
|  | Adult (≥18) | 43 | 12 | 31 | 1.09 (0.09–13.45) |  |
| Phenotype |  |  |  |  |  |  |
|  | Brain/Brainstem | 16 | 4 | 12 | 1.00 (reference) | — |
|  | ADEM | 16 | 11 | 5 | 0.04 (0.002–1.00) | 0.0503 |
|  | ON | 14 | 2 | 18 | 1.70 (0.08–37.57) | 0.7357 |
|  | TM | 5 | 5 | 9 | 0.20 (0.01–7.72) | 0.3875 |
|  | ADEM+ON | 15 | 1 | 14 | 3.39 (0.08–146.58) | 0.5250 |
|  | ON+TM | 10 | 2 | 8 | 5.28 (0.10–279.55) | 0.4111 |
|  | Mixed | 20 | 3 | 2 | 4.32 (0.22–85.67) | 0.3373 |
| ^Analysis was with a one-stage IPD meta-analysis with multivariable logistic mixed-effects regression. There was substantial heterogeneity (I^2^=82.9%).  *Minimum subgroup sample size criterion (n ≥5); phenotype ‘Other’ was excluded due to insufficient participant numbers (n=2) | | | | | | |

**Online supplemental table 8: Random-effects meta-analysis of means to investigate serum GFAP and NfL biomarker levels during different disease activity**

| **Predictor** | **Study** | **Age Category** | **Remission** | | | | **Attack** | | | |
| --- | --- | --- | --- | --- | --- | --- | --- | --- | --- | --- |
|  |  |  | **Samples (n)** | **Mean** | **SD (±)** | **Overall mean (95% CI)** | **Samples (n)** | **Mean** | **SD (±)** | **Overall mean (95% CI)** |
| **GFAP** | Kim, et al., 2020 ^4^ | Adult | 9 | 75.33 pg/mL | 20.17 pg/mL | 97.78 (75.60– 119.97) pg/mL | 7^ | 89.71 pg/mL | 21.65 pg/mL | 176.75 (64.99–288.51) pg/mL |
|  | Chang, et al., 2021 ^10^ | Combined | 19 | 158.42 pg/mL | 232.22 pg/mL |  | 23^ | 309.65 pg/mL | 367.19 pg/mL |  |
|  | Hyun, et al., 2021 ^13^ | Adult | 54 | 107.07 pg/mL | 47.14 pg/mL |  | 17 | 185.88 pg/mL | 81.25 pg/mL |  |
|  | Aktas, et al., 2023 ^68^ | Adult | 8 | 107.14 pg/mL | 42.71 pg/mL |  | — | — | — |  |
| **NfL** | Kim, et al., 2020 ^4^ | Adult | 9 | 11.11 pg/mL | 4.99 pg/mL | 17.25 (10.12–24.37) pg/mL | 7^ | 18.72 pg/mL | 23.11 pg/mL | 71.45 (40.44–103.46) pg/mL |
|  | Chang, et al., 2021 ^10^ | Combined | 19 | 44.16 pg/mL | 78.08 pg/mL |  | 23^ | 68.26 pg/mL | 84.83 pg/mL |  |
|  | Hyun, et al., 2021 ^13^ | Adult | 55 | 22.31 pg/mL | 35.49 pg/mL |  | 17 | 164.76 pg/mL | 154.12 pg/mL |  |
|  | Luo, et al., 2021^29^ | Combined | 47 | 13.00 pg/mL | 11.71 pg/mL |  | 22 | 59.55 pg/mL | 60.58 pg/mL |  |
|  | Aktas, et al., 2023 ^68^ | Adult | 8 | 30.29 pg/mL | 27.91 pg/mL |  | — | — | — |  |
|  | Horellou, et al., 2023 ^84^ | Paediatric | — | — | — |  | 15^ | 89.72 pg/mL | 22.38 pg/mL |  |
|  | Wendel, et al., 2022 ^93^ | Paediatric | — | — | — |  | 19^ | 73.68 pg/mL | 87.77 pg/mL |  |

^Sample collected within 3-months of last clinical symptoms

**Online supplemental table 9: Qualitative assessment of studies reporting novel biomarkers.**

| **Biomarker** | **Sample Size (n)** | **Study** | **Specimen** | **Key Findings** | **Classification of Evidence*** |
| --- | --- | --- | --- | --- | --- |
| ***Properties of serum MOG-IgG*** | | | | | |
| MOG epitope binding pattern | 55 | ^57^ | Serum | - 0% (0/10) of individuals with non-P42 MOG-IgG had a monophasic course compared to 18.3% (2/11) with P42 MOG-IgG (p=0.476) - P42 MOG-IgG was associated with fewer total attacks during follow-up (median 2.0, IQR 1.8-3.5) compared to non-P42 MOG-IgG (median 2.5, IQR 2.0-5.0) (p=0.722) | Class III |
|  | 202 | ^89^ | Serum | - Non-P42 MOG-IgG conferred significantly increased risk for relapsing course compared to P42 MOG-IgG (HR 1.7, 95% CI 1.15–2.60) (p=0.009) - Among individuals with uON at onset, non-P42 MOG-IgG conferred significantly increased risk of relapsing course compared to P42 MOG-IgG (HR 2.7, 95% CI 1.06– 6.98) (p=0.038) - Epitope binding pattern was highly stable over time | Class II |
| ***Immune cells*** | | | | | |
| Complement-dependent cytotoxicity (CDC) and antibody-dependent cellular phagocytosis (ADCP) | 56 total; 25 MOGAD | ^91^ | Serum | - High-throughput assays assessed effector functions (complement activation (CA), CDC, ADCP, and antibody-dependent cellular cytotoxicity (ADCC)) of MOG-IgG from sera of individuals with MOGAD - Engagement of effector functions was bimodal; 67% (12.18) of serum samples induced CDC while 33% (6/18) did not - Magnitude of CDC and ADCP was significantly elevated closer to relapse (p=0.058 and p=0.011, respectively) - CDC and ADCP assays have potential to predict disease activity as well as individual response to complement inhibitor therapies | Class III |
| PBMCs | 29 total; 12 MOGAD | ^9^ | Serum | - PBMCs collected at onset were stimulated with rh-MOG - Th17-cells increased significantly in individuals with a monophasic course but not a relapsing course (*p* = 0.03 and p=0.25, respectively) - CD4+Foxp3+ Tregs increased significantly in individuals with a monophasic course but not a relapsing course (*p* = 0.046 and p=0.375, respectively) - CD45RA-Foxp3+ Tregs decreased significantly in individuals with a relapsing course but not with a monophasic course (*p* = 0.021; p-value for monophasic group not stated) | Class III |
|  | 96 total; 26 MOGAD | ^24^ | Serum | - Th17-cells were significantly increased in MOGAD, AQP4-IgG seropositive NMOSD, and MS compared to HCs at attack (p=0.001, p<0.001, and p=0.005, respectively) and remission (p=0.033, p=0.005, and p=0.004, respectively) - Th17/Treg ratios were significantly increased in MOGAD, AQP4-IgG seropositive NMOSD, and MS compared to HCs at attack (p=0.004, p<0.001, and p=0.006, respectively) and remission (p=0.019, p<0.001, and p=0.005, respectively) - Th17-cells were significantly increased in MOGAD, AQP4-IgG seropositive NMOSD, and MS at attack compared to remission (paired sample comparison) (p=0.015, p=0.012, and p=0.018, respectively) | Class II |
|  | 41 total; 17 MOGAD | ^30^ | Serum | - Plasmablasts were significantly lower at attack in MOGAD compared to AQP4-IgG seropositive NMOSD (*p* < 0.05) - Transitional B-cells were significantly elevated at remission in MOGAD compared to HCs and AQP4-IgG seropositive NMOSD (all p<0.01) - MOGAD attack and remission B- and T-cell subsets were not explicitly compared | Class II |
| Peripheral blood count index ratios, including:   - Neutrophil percentage (N%) - Neutrophil-to-lymphocyte ratio (NLR) - Platelet-to-lymphocyte ratio (PLR) - Monocyte-to-lymphocyte ratio (MLR) - Eosinophil-to-lymphocyte ratio (ELR) - Systemic immune-inflammation index (SII) - Systemic inflammation response index (SIRI) | 156 total; 17 MOGAD | ^47^ | Serum | - NLR was significantly elevated at attack compared to remission in MOGAD (p<0.001) - NLR was significantly elevated at attack in MOGAD compared to MS (p<0.001) | Class II |
|  | 304 total; 26 MOGAD | ^69^ | Serum | - Total white blood cell count, neutrophil count, monocyte count, NLR, and MLR were all significantly elevated at attack compared to controls (p=0.0001, p<0.0001, p=0.0191, p=0.0002, and p=0.0320, respectively) | Class II |
|  | 81 total; 31 MOGAD | ^83^ | Serum | - NLR, PLR, and MLR collected at onset were significantly elevated in MOGAD compared to HCs (all p<0.001) - NLR and PLR collected at onset were significantly elevated in MOGAD compared to MS (p<0.001 and p=0.001, respectively) - PLR was significantly positively associated with relapsing course in MOGAD (OR=1.016, 95% CI 1.001–1.031) (p=0.038) | Class II |
|  | 39 | ^103^ | Serum | - NLR, PLR and N% were significantly elevated at attack compared to remission (all p<0.001) - ELR was significantly lower at attack compared to remission (p<0.001) | Class II |
|  | 125 total;  25 MOGAD | ^105^ | Serum | - Onset MLR was significantly lower in individuals with relapsing MOGAD (median 0.18, IQR 0.14-0.26) compared to monophasic MOGAD (median 0.29, IQR 0.24-0.44) (p=0.013) - ROC analysis found that onset MLR predicted relapsing course with sensitivity and specificity of 75.0% and 88.2%, respectively - Onset MLR < 0.200 was significantly associated with relapsing course (p=0.005) - There were no statistically significant associations with NLR, PLR, SII, or SIRI and disease course | Class II |
| ***Immune molecules*** | | | | | |
| Complement | 210 total; 109 MOGAD | ^44^ | Serum | - Complement proteins C5a, SC5b9, Ba, and Bb were significantly elevated in MOGAD compared to paediatric controls (PCs), relapsing MS (RMS), anti-aquaporin-4 antibody (AQP4-IgG) seropositive neuromyelitis optica spectrum disorder (NMOSD), and healthy donors (HDs) (all p≤0.0001) - Complement protein C3a was significantly elevated in MOGAD compared to PCs, RMS, and HDs (p≥0.0001, p≤0.001, and p≤0.0001, respectively) but not significantly different to AQP4-IgG NMOSD - Complement protein factor H was significantly elevated in MOGAD compared to RMS (p≤0.001) but not significantly different to PCs, AQP4-IgG seropositive NMOSD, or HDs - Within the MOGAD group, no difference in any complement protein levels at attack compared to remission nor individuals with monophasic compared to relapsing course | Class II |
| Cytokines and chemokines | 93 total; 21 MOGAD | ^3^ | Serum | - IL-1β, IL-5, IL-6, IL-10, IL-12p70, IL-17A, tumour necrosis factor (TNF)-α, and interferon (IFN)-γ levels were measured in attack and remission samples - IL-1β (macrophage and dendritic cell-related cytokine) was significantly elevated in MOGAD compared to AQP4-IgG seropositive NMOSD (p<0.001) - IL-10 (regulatory T-cell-related cytokine) was significantly elevated in MOGAD compared to MS and other inflammatory demyelinating diseases (IDDs) (p=0.004 and p=0.002, respectively) - IL-12p70 (macrophage and dendritic cell-related cytokine) was significantly elevated in MOGAD compared to AQP4-IgG seropositive NMOSD and IDDs (both p=0.003) - No significant difference between groups in levels of TNF-α, IFN-γ, IL-5, IL-6, and IL-17A - Th1 dominance was more prominent in MOGAD compared to MS, but similar to AQP4-IgG seropositive NMOSD - IL-1β levels were significantly increased at attack compared to remission most markedly in MOGAD as well as AQP4-IgG seropositive NMOSD, and MS (all p<0.001) | Class I |
|  | 134 total; 40 MOGAD | ^63^ | Serum | - 65 cytokines, chemokines, and related molecules like growth factors and soluble receptors were measured in attack and remission samples - Both antibody-associated demyelinating diseases, MOGAD and AQP4-IgG seropositive NMOSD, had similar patterns of increased cytokines and chemokines compared to MS - IL-6 was significantly elevated in MOGAD compared to MS (p<0.001) but not significantly different to AQP4-IgG seropositive NMOSD, carrying implications for potential therapeutic IL-6 blockade - Within the MOGAD group, no significant differences in levels measured at attack compared to remission nor between individuals with monophasic compared to relapsing course | Class III |
|  | 69 total; 11 MOGAD | ^90^ | Serum and CSF | - IL-2, IL- 4, IL-6, IL-10, and IL-33 levels were measured at attack, pre- and post-treatment with IV methylprednisolone; there were no remission samples for comparison - Pre-treatment, serum IL-2, IL-6, and IL-10 were significantly increased in MOGAD compared to healthy controls (HCs) (p<0.01, p<0.05, and p<0.01, respectively), while IL-4 and IL-33 were significantly decreased in MOGAD compared to HCs (both p<0.05) - Post-treatment, serum IL-6, IL-10, and IL-33 were significantly increased in MOGAD compared to HCs (p<0.01, p<0.05, and p<0.05, respectively), while IL-2 and IL-4 were significantly decreased in MOGAD compared to HCs (both p<0.05) - Pre-treatment, CSF cytokines were all significantly increased (IL-33 > IL-10 > IL-4 > IL-2) in MOGAD (p<0.01, p<0.01, p<0.05, and p<0.05, respectively) and AQP4-IgG seropositive NMOSD (p<0.05, p<0.01, p<0.05, and p<0.05, respectively) compared to HCs, but more so in MOGAD - Metrics of BBB dysfunction (CSF QAlb, IgG index, and 24-h IgG synthesis rate) were all significantly elevated in MOGAD (all p<0.01) and AQP4-IgG seropositive NMOSD (p<0.05, p<0.01, and p<0.05, respectively) compared to HCs, but more so in MOGAD | Class III |
|  | 55 total; 18 MOGAD | ^107^ | Serum and CSF | - Cytokines relevant to vascular remodelling were measured in samples obtained pre-treatment and at attack or near attack (median 14 (range 1-81) days from attack to sampling); there were no remission samples for comparison - Serum hepatocyte growth factor (HGF) was significantly elevated in both MOGAD and AQP4-IgG seropositive NMOSD compared to HCs (p=0.0022 and p<0.001, respectively) - Serum fibroblast growth factor-2 (FGF-2) was significantly elevated in MOGAD compared to HCs (p=0.0070) - No significant difference in serum or CSF IL-6 between MOGAD and AQP4-IgG seropositive NMOSD; no HC measurement for comparison | Class III |
| Lipocalin-2 (LCN2) | 39 total; 6 MOGAD | ^97^ | Serum | - LCN2 levels were significantly higher in MOG-IgG seropositive ON (mean 50.96 ng/mL, SD not stated) at onset attack compared to MOG-IgG seronegative ON (mean 37.60 ng/mL) and HCs (30.86 ng/mL) (p=0.037) - LCN2 levels significantly positively correlated with MOG-IgG titres (r=0.553, p=0.0141) - ROC analysis found that LCN2 level of 36.2 ng/mL predicted ON relapse with sensitivity and specificity of 62.5% and 81.8%, respectively; this was not statistically significant (p=0.133) | Class III |
| sTREM2 | 38 total; 19 MOGAD | ^76^ | Serum and CSF | - sTREM2 levels were significantly elevated in serum and CSF of children with MOGAD compared to non-neuroinflammatory disorder controls (p=0.0012 and p<0.001, respectively); all samples were collected at attack | Class III |
| TNFAIP3 | 68 total; 24 MOGAD | ^28^ | Serum | - TNFAIP3 levels were significantly reduced at attack compared to remission and HCs (p=0.04 and p=0.0001, respectively) | Class II |
| ***Neurological injury molecules*** | | | | | |
| GFAP | 49 total; 16 MOGAD | ^4^ | Serum | - No significant difference between GFAP levels in MOGAD at attack and remission | Class I |
|  | 152 total; 42 MOGAD | ^10^ | Serum | - GFAP levels were significantly elevated in AQP4-IgG seropositive NMOSD (median 274.1, IQR 109.2-1680.6 pg/mL) and MOGAD (median 136.7, IQR 97.8-220.1 pg/mL) compared to HCs (median 61.4, IQR 49.7-81.0 pg/mL) (all p < 0.001) | Class I |
|  | 41 total; 15 MOGAD | ^13^ | Serum | - GFAP levels were not significantly elevated at attack in MOGAD | Class II |
|  | 225 total; 7 MOGAD | ^68^ | Serum | - GFAP levels were elevated (> 2 SDs from HD means) in 14% (1/7) of individuals with MOGAD at baseline - There was no significant attack-related pattern of GFAP in MOGAD | Class I |
| NfL | 49 total; 16 MOGAD | ^4^ | Serum | - No significant difference between NfL levels at attack and remission | Class I |
|  | 152 total; 42 MOGAD | ^10^ | Serum | - NfL levels were significantly higher in AQP4-IgG seropositive NMOSD (median 17.6, IQR 9.6–48.1 pg/mL), MOGAD (median 27.2, IQR 10.8–54.8 pg/mL), and RRMS (median 24.5, IQR 14.5–58.3 pg/mL) compared to HCs (median 7.4, IQR 5.6–9.4 pg/mL) (all p<0.001) - NfL levels were significantly higher at attack (median 34.1, IQR 17.6–64.3 pg/mL) compared to remission (median 2.5, IQR 9.1–48.4 pg/mL) in MOGAD (p=0.049) | Class I |
|  | 41 total; 15 MOGAD | ^13^ | Serum | - NfL levels were elevated in all (17/17) attack samples compared to 24% (14/59) of remission samples (p<0.0001) | Class II |
|  | 120 total; 49 MOGAD | ^29^ | Serum | - NfL levels in adults were significantly higher at attack (median 31.0, IQR 15.8-81.2 pg/mL) compared to remission (median 8.1, IQR 5.7-14.4 pg/mL) and HCs (median 10.3, IQR 8.1-13.3 pg/mL) (p=0.001 and p=0.004, respectively) - NfL levels in children were significantly higher at attack (median 46.8, IQR 5.8-130.2 pg/mL) compared to remission (median 13.1, IQR 4.7-35.7 pg/mL) and HCs (median 8.2, IQR 6.4-11.3 pg/mL) (p=0.001 and p=0.007, respectively) | Class I |
|  | 36 total; 18 MOGAD | ^32^ | Serum | - NfL levels were increased at onset then stable or decreased with no significant elevation at subsequent attacks | Class III |
|  | 76 total; 38 MOGAD | ^34^ | Serum | - NfL levels at onset were significantly higher in individuals with severe attacks (median 15.3 pg/mL, range 4-101.5 pg/mL) compared to individuals with mild-to-moderate attacks (median 7.3 pg/mL, range 2.1–23.0 pg/mL) (p=0.002), independent of age and sex - NfL levels at onset did not significantly correlate with subsequent relapse rate | Class II |
|  | 225 total; 7 MOGAD | ^68^ | Serum | - NfL levels were elevated (>2 SDs from HD means) in 43% (3/7) of individuals with MOGAD at baseline - There was no significant attack-related pattern of NFL in MOGAD | Class I |
|  | 27 total; 15 MOGAD | ^84^ | Serum | - NfL levels were significantly elevated within 3 months of onset in MOGAD (mean 89.72 ± 22.38 pg/mL) compared to HCs (mean 12.47 ± 2.47 pg/mL) (p=0.0012) | Class II |
|  | 164 total; 19 MOGAD | ^93^ | Serum | - NfL levels collected within 3 months of onset were significantly higher in children with MOGAD (median 56.7, range 4.1–372.8 pg/mL), other ADS (median 26.5, range 1.5–2444.3 pg/mL), and MS (median 39.1, range 4.2–474.7 pg/mL) compared to controls with other neurological diseases (median 7.5, range 3.0–31.8 pg/mL) (all p<0.001) | Class I |
| Tau | 49 total; 16 MOGAD | ^4^ | Serum | - Tau levels were significantly higher at attack (median 0.5, IQR 0.4–0.5 pg/mL) compared to remission (median 0.2, IQR 0.1–0.3 pg/mL) (p=0.027) | Class I |
|  | 225 total; 7 MOGAD | ^68^ | Serum | - Tau levels were not elevated (> 2 SDs of HD means) at baseline in any individuals with MOGAD nor was any significant attack-related pattern observed | Class I |
| Ubiquitin Carboxy-terminal Hydrolase L1 (UCHL1) | 225 total; 7 MOGAD | ^68^ | Serum | - UCHL1 levels were not elevated (> 2 SDs of HD means) at baseline in any individuals with MOGAD nor was any significant attack-related pattern observed | Class I |
| ***Genetics*** | | | | | |
| HLA genotype | 576 total; 95 MOGAD | ^33^ | Serum | - Frequency of DQB1*05:02 allele was significantly higher at 18.95% in MOGAD compared to 10.71% in controls (OR=1.95, 95% CI 1.25–3.0) (p=0.002) - Age subgroup analysis revealed a significant association for paediatric-onset MOGAD with DQB1*05:02 (OR=2.43, 95% CI 1.39–4.11) (p=0.001) and DRB1*16:02 (OR=3.28, 95% CI 1.55–6.25) (p=0.001) but no significant associations for adult-onset MOGAD - 80% (8/10) of paediatric-onset MOGAD carriers of the DQB1*05:02–DRB1*16:02 haplotype had a relapsing course compared to 37% (15/41) of non-carriers (p=0.030) | Class II |
| ***Metabolic and endocrine molecules*** | | | | | |
| Homocysteine | 20 | ^87^ | Serum | - Homocysteine was elevated at onset in 40% (8/20) of individuals with late-onset (≥ 50 years) MOGAD; of those, 87.5% (7/8) had a monophasic course and 12.5% (1/8) had a relapsing course - Homocysteine was normal at onset in 60% (12/20) of individuals with late-onset MOGAD; of those, 50% (6/6) had a monophasic course and 50% (6/6) had a relapsing course - No findings reached statistical significance (p=0.158) | Class III |
| Prolactin | 138 total; 15 MOGAD | ^75^ | Serum | - No significant difference between prolactin levels in MOGAD at attack and remission | Class II |
| Thiol | 85 total; 8 MOGAD | ^23^ | Serum | - Total thiol and native thiol levels were significantly lower in samples collected at attack compared to remission in a combined cohort of MOGAD, AQP4-IgG seropositive NMOSD, and MS | Class III |
| Thyroid function profile | 261 total; 26 MOGAD | ^64^ | Serum | - FT4 levels were significantly higher in relapsing MOGAD (mean 16.46 ± 3.14 pM) compared to monophasic MOGAD (mean 13.68 ± 1.46 pM) (p=0.03) - ROC analysis found that FT4 level 15.125 pM predicted relapsing disease course with sensitivity and specificity of 72.7% and 87.5%, respectively | Class III |
| ***Extracellular matrix molecules*** | | | | | |
| MFAP4 | 152 total; 22 MOGAD | ^88^ | CSF | MFAP4 levels were significantly reduced at attack compared to remission (p=0.001) | Class II |

*Classification of evidence was assessed using the American Academy of Neurology (AAN) Criteria for Rating Diagnostic Accuracy Studies.

**Online supplemental references**

1. Banwell B, Bennett JL, Marignier R, et al. Diagnosis of myelin oligodendrocyte glycoprotein antibody-associated disease: International MOGAD Panel proposed criteria. The Lancet Neurology. 2023;22(3):268-82.

2. Baumann M, Hennes E-M, Schanda K, et al. Children with multiphasic disseminated encephalomyelitis and antibodies to the myelin oligodendrocyte glycoprotein (MOG): Extending the spectrum of MOG antibody positive diseases. Multiple sclerosis (Houndmills, Basingstoke, England). 2016;22(14):1821-9.

3. Kwon YN, Kim B, Ahn S, et al. Serum level of IL-1β in patients with inflammatory demyelinating disease: Marked upregulation in the early acute phase of MOG antibody associated disease (MOGAD). Journal of Neuroimmunology. 2020;348.

4. Kim H, Lee E-J, Kim S, et al. Serum biomarkers in myelin oligodendrocyte glycoprotein antibody-associated disease. Neurology(R) neuroimmunology & neuroinflammation. 2020;7(3).

5. Wendel E-M, Baumann M, Barisic N, et al. High association of MOG-IgG antibodies in children with bilateral optic neuritis. European journal of paediatric neurology : EJPN : official journal of the European Paediatric Neurology Society. 2020;27:86-93.

6. Pröbstel AK, Dornmair K, Bittner R, et al. Antibodies to MOG are transient in childhood acute disseminated encephalomyelitis. Neurology. 2011 Aug 9;77(6):580-8.

7. Hyun J-W, Woodhall MR, Kim S-H, et al. Longitudinal analysis of myelin oligodendrocyte glycoprotein antibodies in CNS inflammatory diseases. Journal of neurology, neurosurgery, and psychiatry. 2017;88(10):811-7.

8. Hoftberger R, Sepulveda M, Armangue T, et al. Antibodies to MOG and AQP4 in adults with neuromyelitis optica and suspected limited forms of the disease. Multiple sclerosis (Houndmills, Basingstoke, England). 2015;21(7):866-74.

9. Horellou P, de Chalus A, Giorgi L, et al. Regulatory T Cells Increase After rh-MOG Stimulation in Non-Relapsing but Decrease in Relapsing MOG Antibody-Associated Disease at Onset in Children. Frontiers in immunology. 2021;12:679770.

10. Chang X, Huang W, Wang L, et al. Serum Neurofilament Light and GFAP Are Associated With Disease Severity in Inflammatory Disorders With Aquaporin-4 or Myelin Oligodendrocyte Glycoprotein Antibodies. Frontiers in immunology. 2021;12:647618.

11. Jarius S, Lechner C, Wendel EM, et al. Cerebrospinal fluid findings in patients with myelin oligodendrocyte glycoprotein (MOG) antibodies. Part 2: Results from 108 lumbar punctures in 80 pediatric patients. Journal of neuroinflammation. 2020;17(1):262.

12. Cobo-Calvo A, Ruiz A, D'Indy H, et al. MOG antibody-related disorders: common features and uncommon presentations. Journal of neurology. 2017;264(9):1945-55.

13. Hyun J-W, Kim SY, Kim Y, et al. Absence of attack-independent neuroaxonal injury in MOG antibody-associated disease: Longitudinal assessment of serum neurofilament light chain. Multiple sclerosis (Houndmills, Basingstoke, England). 2021:13524585211063756.

14. Ikeda A, Watanabe Y, Kaba H, Kaneko K, Takahashi T, Takeshita S. MRI findings in pediatric neuromyelitis optica spectrum disorder with MOG antibody: Four cases and review of the literature. Brain & development. 2019;41(4):367-72.

15. Lopez-Chiriboga AS, Majed M, Fryer J, et al. Association of MOG-IgG Serostatus With Relapse After Acute Disseminated Encephalomyelitis and Proposed Diagnostic Criteria for MOG-IgG-Associated Disorders. JAMA neurology. 2018;75(11):1355-63.

16. Waters P, Fadda G, Woodhall M, et al. Serial Anti-Myelin Oligodendrocyte Glycoprotein Antibody Analyses and Outcomes in Children with Demyelinating Syndromes. JAMA Neurology. 2020;77(1):82-93.

17. Jurynczyk M, Messina S, Woodhall MR, et al. Clinical presentation and prognosis in MOG-antibody disease: a UK study. Brain (London, England : 1878). 2017;140(12):3128-38.

18. Hino-Fukuyo N, Haginoya K, Takahashi T, et al. Long-term outcome of a group of Japanese children with myelin-oligodendrocyte glycoprotein encephalomyelitis without preventive immunosuppressive therapy. Brain & development. 2019;41(9):790-5.

19. Serin HM, Yilmaz S, Simsek E, et al. Clinical spectrum, treatment and outcome of myelin oligodendrocyte glycoprotein (MOG) antibody-associated disease in children: a tertiary care experience. Acta neurologica Belgica. 2021;121(1):231-9.

20. Jarius S, Pellkofer H, Siebert N, et al. Cerebrospinal fluid findings in patients with myelin oligodendrocyte glycoprotein (MOG) antibodies. Part 1: Results from 163 lumbar punctures in 100 adult patients. Journal of neuroinflammation. 2020;17(1):261.

21. Jarius S, Ruprecht K, Kleiter I, et al. MOG-IgG in NMO and related disorders: a multicenter study of 50 patients. Part 1: Frequency, syndrome specificity, influence of disease activity, long-term course, association with AQP4-IgG, and origin. J Neuroinflammation. 2016 Sep 26;13(1):279.

22. Oliveira LM, Apostolos-Pereira SL, Pitombeira MS, Bruel Torretta PH, Callegaro D, Sato DK. Persistent MOG-IgG positivity is a predictor of recurrence in MOG-IgG-associated optic neuritis, encephalitis and myelitis. Multiple sclerosis (Houndmills, Basingstoke, England). 2019;25(14):1907-14.

23. Arslan B, Arslan GA, Tuncer A, Karabudak R, Dincel AS. Evaluation of Thiol Homeostasis in Multiple Sclerosis and Neuromyelitis Optica Spectrum Disorders. Frontiers in neurology. 2021;12:716195.

24. Liu J, Mori M, Sugimoto K, et al. Peripheral blood helper T cell profiles and their clinical relevance in MOG-IgG-associated and AQP4-IgG-associated disorders and MS. Journal of neurology, neurosurgery, and psychiatry. 2020;91(2):132-9.

25. de Mol CL, Wong Y, van Pelt ED, et al. The clinical spectrum and incidence of anti-MOG-associated acquired demyelinating syndromes in children and adults. Mult Scler. 2020 Jun;26(7):806-14.

26. Rostasy K, Mader S, Schanda K, et al. Anti-myelin oligodendrocyte glycoprotein antibodies in pediatric patients with optic neuritis. Archives of neurology. 2012;69(6):752-6.

27. Rostasy K, Mader S, Hennes EM, et al. Persisting myelin oligodendrocyte glycoprotein antibodies in aquaporin-4 antibody negative pediatric neuromyelitis optica. Multiple sclerosis (Houndmills, Basingstoke, England). 2013;19(8):1052-9.

28. Saxena S, Lokhande H, Gombolay G, Raheja R, Rooney T, Chitnis T. Identification of TNFAIP3 as relapse biomarker and potential therapeutic target for MOG antibody associated diseases. Sci Rep-uk. 2020;10(1):12405.

29. Luo W, Chen Y, Mao S, et al. Serum neurofilament light chain in adult and pediatric patients with myelin oligodendrocyte glycoprotein antibody-associated disease: Correlation with relapses and seizures. Journal of neurochemistry. 2021.

30. Tanaka S, Hashimoto B, Izaki S, Oji S, Fukaura H, Nomura K. Clinical and immunological differences between MOG associated disease and anti AQP4 antibody-positive neuromyelitis optica spectrum disorders: Blood-brain barrier breakdown and peripheral plasmablasts. Multiple sclerosis and related disorders. 2020;41:102005.

31. Han JY, Kim SY, Kim H, et al. Clinico-radiological characteristics of anti-myelin oligodendrocyte glycoprotein antibody-associated autoimmune encephalitis in children. Developmental medicine and child neurology. 2022.

32. Mariotto S, Gastaldi M, Grazian L, et al. NfL levels predominantly increase at disease onset in MOG-Abs-associated disorders. Multiple sclerosis and related disorders. 2021;50:102833.

33. Sun X, Qiu W, Wang J, et al. Myelin oligodendrocyte glycoprotein-associated disorders are associated with HLA subtypes in a Chinese paediatric-onset cohort. Journal of neurology, neurosurgery, and psychiatry. 2020;91(7):733-9.

34. Mariotto S, Ferrari S, Gastaldi M, et al. Neurofilament light chain serum levels reflect disease severity in MOG-Ab associated disorders. Journal of Neurology, Neurosurgery and Psychiatry. 2019;90(11):1293-6.

35. Alshamrani F, Alnajashi H, Shosha E, Casserly C, Morrow SA. Case Series: Myelin Oligodendrocyte Glycoprotein-Immunoglobulin G-Related Disease Spectrum. Frontiers in neurology. 2020;11:89.

36. Dale RC, Tantsis EM, Merheb V, et al. Antibodies to MOG have a demyelination phenotype and affect oligodendrocyte cytoskeleton. Neurology(R) neuroimmunology & neuroinflammation. 2014;1(1):e12.

37. Lui A, Chong J, Flanagan E, et al. High titers of myelin oligodendrocyte glycoprotein antibody are only observed close to clinical events in pediatrics. Multiple sclerosis and related disorders. 2021;56:103253.

38. Dubey D, Pittock SJ, Krecke KN, et al. Clinical, Radiologic, and Prognostic Features of Myelitis Associated With Myelin Oligodendrocyte Glycoprotein Autoantibody. JAMA neurology. 2019;76(3):301-9.

39. Hennes E-M, Baumann M, Schanda K, et al. Prognostic relevance of MOG antibodies in children with an acquired demyelinating syndrome. Neurology. 2017;89(9):900-8.

40. Tea F, Lopez JA, Ramanathan S, et al. Characterization of the human myelin oligodendrocyte glycoprotein antibody response in demyelination. Acta neuropathologica communications. 2019;7(1):145.

41. Senanayake B, Jitprapaikulsan J, Aravinthan M, et al. Seroprevalence and clinical phenotype of MOG-IgG-associated disorders in Sri Lanka. Journal of neurology, neurosurgery, and psychiatry. 2019;90(12):1381-3.

42. Wegener-Panzer A, Cleaveland R, Wendel E-M, et al. Clinical and imaging features of children with autoimmune encephalitis and MOG antibodies. Neurology(R) neuroimmunology & neuroinflammation. 2020;7(4).

43. Zhou J, Lu X, Ji T, et al. Follow-up study on Chinese children with relapsing MOG-IgG-associated central nervous system demyelination. Multiple Sclerosis and Related Disorders. 2019;28:4-10.

44. Keller CW, Lopez JA, Wendel E-M, et al. Complement Activation Is a Prominent Feature of MOGAD. Annals of neurology. 2021;90(6):976-82.

45. Baumann M, Sahin K, Lechner C, et al. Clinical and neuroradiological differences of paediatric acute disseminating encephalomyelitis with and without antibodies to the myelin oligodendrocyte glycoprotein. Journal of neurology, neurosurgery, and psychiatry. 2015;86(3):265-72.

46. Ramanathan S, Mohammad S, Tantsis E, et al. Clinical course, therapeutic responses and outcomes in relapsing MOG antibody-associated demyelination. Journal of Neurology, Neurosurgery &amp; Psychiatry. 2018;89(2):127.

47. Benetou C, Berti F, Hemingway C, Hacochen Y, Lim M. Neutrophil-to-lymphocyte ratio correlates with disease activity in myelin oligodendrocyte glycoprotein antibody associated disease (MOGAD) in children. Multiple Sclerosis and Related Disorders. 2020;45:102345.

48. Armangue T, Olive-Cirera G, Martinez-Hernandez E, et al. Associations of paediatric demyelinating and encephalitic syndromes with myelin oligodendrocyte glycoprotein antibodies: a multicentre observational study. The Lancet Neurology. 2020;19(3):234-46.

49. Nakajima H, Motomura M, Tanaka K, et al. Antibodies to myelin oligodendrocyte glycoprotein in idiopathic optic neuritis. Bmj Open. 2015;5(4):e007766.

50. Mao L, Yang L, Kessi M, et al. Myelin Oligodendrocyte Glycoprotein (MOG) Antibody Diseases in Children in Central South China: Clinical Features, Treatments, Influencing Factors, and Outcomes. Frontiers in neurology. 2019;10:868.

51. Siritho S, Sato DK, Kaneko K, Fujihara K, Prayoonwiwat N. The clinical spectrum associated with myelin oligodendrocyte glycoprotein antibodies (anti-MOG-Ab) in Thai patients. Multiple sclerosis (Houndmills, Basingstoke, England). 2016;22(7):964-8.

52. Zhang M, Du X, Zhou S, et al. Clinical characteristics, disease course, and outcomes of paediatric patients with myelin oligodendrocyte glycoprotein-Ab associated disease: A retrospective clinical study. Journal of clinical neuroscience : official journal of the Neurosurgical Society of Australasia. 2021;94:1-7.

53. Jitprapaikulsan J, Chen JJ, Flanagan EP, et al. Aquaporin-4 and Myelin Oligodendrocyte Glycoprotein Autoantibody Status Predict Outcome of Recurrent Optic Neuritis. Ophthalmology. 2018 2018/10/01/;125(10):1628-37.

54. Huda S, Whittam D, Jackson R, et al. Predictors of relapse in MOG antibody associated disease: a cohort study. BMJ Open. 2021;11(11):e055392.

55. Dauby S, Dive D, Lutteri L, et al. Comparative study of AQP4-NMOSD, MOGAD and seronegative NMOSD: a single-center Belgian cohort. Acta neurologica Belgica. 2021.

56. Solmaz I, Oncel IH, Konuskan B, et al. Role of serostatus in pediatric neuromyelitis optica spectrum disorders: A nationwide multicentric study. Multiple sclerosis and related disorders. 2023;77:104847.

57. Seok JM, Jeon MY, Chung YH, et al. Clinical characteristics of myelin oligodendrocyte glycoprotein antibody-associated disease according to their epitopes. Frontiers in neurology. 2023;14:1200961.

58. Martin K, Srikanth P, Kanwar A, Falardeau J, Pettersson D, Yadav V. Clinical and radiographic features of a cohort of adult and pediatric subjects in the Pacific Northwest with myelin oligodendrocyte glycoprotein antibody-associated disease (MOGAD). Multiple Sclerosis and Related Disorders. 2024;81:105130.

59. Liao D, Zhong L, Yang L, et al. Clinical and radiological features, treatment responses and prognosis in pediatric patients with co-existing anti-N-methyl-D-aspartate receptor and myelin oligodendrocyte glycoprotein antibody-associated encephalitis: A single center study. Multiple Sclerosis and Related Disorders. 2024;81:105133.

60. Nosadini M, Eyre M, Giacomini T, et al. Early Immunotherapy and Longer Corticosteroid Treatment Are Associated With Lower Risk of Relapsing Disease Course in Pediatric MOGAD. Neurology: Neuroimmunology and NeuroInflammation. 2023;10(1):e200065.

61. Kang Q, Liao H, Yang L, et al. Clinical analysis of 173 pediatric patients with antibody-mediated autoimmune diseases of the central nervous system: a single-center cohort study. Frontiers in Immunology. 2023;14:1140872.

62. Yao T, Zeng Q, Xie Y, et al. Clinical analysis of adult MOG antibody-associated cortical encephalitis. Multiple sclerosis and related disorders. 2022;60:103727.

63. Bauer A, Rudzki D, Berek K, et al. Increased peripheral inflammatory responses in myelin oligodendrocyte glycoprotein associated disease and aquaporin-4 antibody positive neuromyelitis optica spectrum disorder. Frontiers in Immunology. 2022;13:1037812.

64. Rechtman A, Zveik O, Haham N, Freidman-Korn T, Vaknin-Dembinsky A. Thyroid hormone dysfunction in MOGAD and other demyelinating diseases. Journal of the Neurological Sciences. 2024;457:122866.

65. Nguyen L, Miles DK, Harder L, et al. Increased Intracranial Pressure in Pediatric Myelin Oligodendrocyte Glycoprotein Antibody-Associated Disease. Neurology(R) neuroimmunology & neuroinflammation. 2024;11(1).

66. Zhangbao J, Huang W, Zhou L, et al. Clinical feature and disease outcome in patients with myelin oligodendrocyte glycoprotein antibody-associated disorder: A Chinese study. Journal of Neurology, Neurosurgery and Psychiatry. 2023;94(10):825-34.

67. Montalvo M, Khattak J, Redenbaugh V, et al. Acute Symptomatic Seizures Secondary to Myelin Oligodendrocyte Glycoprotein Antibody-associated Disorder. Neurology. 2022;98(18 SUPPL).

68. Aktas O, Hartung H-P, Smith MA, et al. Serum neurofilament light chain levels at attack predict post-attack disability worsening and are mitigated by inebilizumab: analysis of four potential biomarkers in neuromyelitis optica spectrum disorder. Journal of neurology, neurosurgery, and psychiatry. 2023;94(9):757-68.

69. Akaishi T, Misu T, Fujihara K, et al. White blood cell count profiles in anti-aquaporin-4 antibody seropositive neuromyelitis optica spectrum disorder and anti-myelin oligodendrocyte glycoprotein antibody-associated disease. Sci Rep-uk. 2023;13(1):6481.

70. Wang W, Yin J, Fan Z, et al. Case Report: Four Cases of Cortical/Brainstem Encephalitis Positive for Myelin Oligodendrocyte Glycoprotein Immunoglobulin G. Frontiers in Neurology. 2022;12:775181.

71. Wendel EM, Thonke HS, Bertolini A, et al. Temporal Dynamics of MOG Antibodies in Children With Acquired Demyelinating Syndrome. Neurology: Neuroimmunology and NeuroInflammation. 2022;9(6):e200035.

72. Guzman J, Vera F, Soler B, et al. Myelin Oligodendrocyte Glycoprotein Antibody-Associated Disease (MOGAD) in Chile: lessons learned from challenging cases. Multiple Sclerosis and Related Disorders. 2023;69:104442.

73. Gastaldi M, Foiadelli T, Greco G, et al. Prognostic relevance of quantitative and longitudinal MOG antibody testing in patients with MOGAD: a multicentre retrospective study. Journal of neurology, neurosurgery, and psychiatry. 2022.

74. Wu Y, Zhou H, Ci X, Lin L, Zhang D, Lu J. Clinical characteristic of myelin oligodendrocyte glycoprotein antibody associated cortical encephalitis in adults and outcomes following glucocorticoid therapy. Frontiers in Aging Neuroscience. 2023;14:1076361.

75. Liu H, Zhang X, Chen W, Xu Y, Lin X, Lin A. The relationship between plasma prolactin levels and clinical manifestations with neuromyelitis optica spectrum disorders. Neurological Sciences. 2024;45(2):699-707.

76. Zhou A, Zhang W, Ren C, Zhou J, Chang H, Ren X. High levels of cerebrospinal fluid soluble triggering receptor expressed on myeloid cells 2 might be a biomarker of activity in pediatric patients with MOG-AD. Frontiers in Pediatrics. 2022;10:908527.

77. Zhou J, Li J, Ren C, et al. Mycophenolate mofetil: An alternative disease-modifying agent for MOG-IgG-associated disorders in childhood: A single-center bidirectional cohort study. Multiple Sclerosis and Related Disorders. 2022;68:104128.

78. Yang Y, Zhang C, Cao C, Su W, Zhao N, Yue W. Clinical Features of Patients with Myelin Oligodendrocyte Glycoprotein Antibody–Associated Disease and Isolated Seizure Symptoms. Neuropsychiatric Disease and Treatment. 2024;20:61-7.

79. Wang X, Zhao R, Yang H, Liu C, Zhao Q. Two rare cases of myelin oligodendrocyte glycoprotein antibody-associated disorder in children with leukodystrophy-like imaging findings. BMC Neurology. 2023;23(1):247.

80. Roy S, Vasileiou E, Barreras P, et al. Longitudinal evaluation of serum MOG-IgG titers in MOGAD after initiation of maintenance immunoglobulin: A case series. Multiple Sclerosis Journal. 2023.

81. Lee W-J, Kwon YN, Kim B, et al. MOG antibody-associated encephalitis in adult: clinical phenotypes and outcomes. Journal of neurology, neurosurgery, and psychiatry. 2023;94(2):102-12.

82. Xu Q, Yang X, Qiu Z, et al. Clinical features of MOGAD with brainstem involvement in the initial attack versus NMOSD and MS. Multiple Sclerosis and Related Disorders. 2023;77:104797.

83. Lin L, Ji M, Wu Y, Hang H, Lu J. Neutrophil to lymphocyte ratio may be a useful marker in distinguishing MOGAD and MS and platelet to lymphocyte ratio associated with MOGAD activity. Multiple Sclerosis and Related Disorders. 2023;71:104570.

84. Horellou P, Flet-Berliac L, Leroy C, et al. Early blood neurofilament light chain and myelin oligodendrocyte glycoprotein antibody levels associate with different disease courses of myelin oligodendrocyte glycoprotein-associated disease in children. Brain communications. 2023;5(2):fcad063.

85. Hacohen Y, Absoud M, Woodhall M, et al. Autoantibody biomarkers in childhood-acquired demyelinating syndromes: Results from a national surveillance cohort. Journal of Neurology, Neurosurgery and Psychiatry. 2014;85(4):456-61.

86. Salunkhe M, Gupta P, Singh RK, et al. Clinical and radiological spectrum of anti-myelin oligodendrocyte glycoprotein (MOG) antibody encephalitis: single-center observational study. Neurological Sciences. 2023;44(7):2475-89.

87. Huang Y, Luo W, Cheng X, et al. Clinical and imaging features of patients with late-onset myelin oligodendrocyte glycoprotein antibody-associated disease. Multiple Sclerosis and Related Disorders. 2024;82:105405.

88. Samadzadeh S, Olesen MN, Wirenfeldt M, et al. Microfibrillar-associated protein 4 as a potential marker of acute relapse in inflammatory demyelinating diseases of the central nervous system: Pathological and clinical aspects. Multiple Sclerosis Journal. 2023;29(14):1721-35.

89. Liyanage G, Trewin BP, Lopez JA, et al. The MOG antibody non-P42 epitope is predictive of a relapsing course in MOG antibody-associated disease. Journal of neurology, neurosurgery, and psychiatry. 2024.

90. Wang M, Xia D, Sun L, Bi J, Xie K, Wang P. Interleukin-33 as a Biomarker Affecting Intrathecal Synthesis of Immunoglobulin in Neuromyelitis Optica Spectrum Disorder and Myelin Oligodendrocyte Glycoprotein Antibody-Associated Disease. European Neurology. 2023;86(4):256-62.

91. Yandamuri SS, Filipek B, Obaid AH, et al. MOGAD patient autoantibodies induce complement, phagocytosis, and cellular cytotoxicity. JCI Insight. 2023;8(11):e165373.

92. Nguyen L, Wang CX, Conger DL, Sguigna PV, Singh S, Greenberg BM. Subclinical optic neuritis in pediatric myelin oligodendrocyte glycoprotein antibody-associated disease. Multiple Sclerosis and Related Disorders. 2023;76:104802.

93. Wendel E-M, Bertolini A, Kousoulos L, et al. Serum neurofilament light-chain levels in children with monophasic myelin oligodendrocyte glycoprotein-associated disease, multiple sclerosis, and other acquired demyelinating syndrome. Multiple sclerosis (Houndmills, Basingstoke, England). 2022;28(10):1553-61.

94. Zhang S, Qiao S, Li H, et al. Risk Factors and Nomogram for Predicting Relapse Risk in Pediatric Neuromyelitis Optica Spectrum Disorders. Frontiers in Immunology. 2022;13:765839.

95. Aubart M, Roux C-J, Durrleman C, et al. Neuroinflammatory Disease following Severe Acute Respiratory Syndrome Coronavirus 2 Infection in Children. The Journal of pediatrics. 2022;247:22-8.e2.

96. Tzanetakos D, Tzartos JS, Vakrakou AG, et al. Cortical involvement and leptomeningeal inflammation in myelin oligodendrocyte glycoprotein antibody disease: A three-dimensional fluid-attenuated inversion recovery MRI study. Multiple sclerosis (Houndmills, Basingstoke, England). 2022;28(5):718-29.

97. Kim J-H, Lee H, Oh J, Suk K, Chun BY. Increased Plasma Lipocalin-2 Levels in Patients with Myelin Oligodendrocyte Glycoprotein-IgG-Positive Optic Neuritis. Journal of clinical medicine. 2022;11(9).

98. Nguyen L, Wang CX, Hernandez RS, Greenberg BM. Clinical analysis of myelin oligodendrocyte glycoprotein antibody-associated disease in a diverse cohort of children: A single-center observational study. Multiple sclerosis and related disorders. 2024;84:105497.

99. Fadda G, Waters P, Woodhall M, et al. Serum MOG-IgG in children meeting multiple sclerosis diagnostic criteria. Multiple sclerosis (Houndmills, Basingstoke, England). 2022:13524585221093789.

100. Vosoughi AR, Muccilli A, Schneider R, Rotstein D, Micieli JA. Recovery of Vision in Myelin Oligodendrocyte Glycoprotein-IgG Optic Neuritis Without Treatment: A Case Series. Journal of Neuro-Ophthalmology. 2023;43(4):E126-E8.

101. Jiang Y, Tan C, Li X, et al. Clinical features of the first attack with leukodystrophy-like phenotype in children with myelin oligodendrocyte glycoprotein antibody-associated disorders. International Journal of Developmental Neuroscience. 2023;83(3):267-73.

102. Zeng W, Yu L, Wu J, et al. Clinical characteristics and long-term follow-up outcomes of myelin oligodendrocyte glycoprotein antibody-associated disease in Han Chinese participants. Medicine (United States). 2023;102(40):E35391.

103. Baek S-I, Ro S, Chung YH, et al. Novel index, neutrophil percentage (%) is a useful marker for disease activity in MOG antibody-associated disease. Multiple sclerosis and related disorders. 2023;76:104796.

104. Singh B, Cruz-Flores S, Chaudhry MR, Piriyawat P, Ponce CP. Psychiatric manifestations of anti-MOG antibody disease. Neuroimmunology Reports. 2022;2:100073.

105. Wang L, Xia R, Li X, Shan J, Wang S. Systemic inflammation response index is a useful indicator in distinguishing MOGAD from AQP4-IgG-positive NMOSD. Frontiers in Immunology. 2023;14:1293100.

106. Song X, Ma J. Clinical characteristics of myelin-oligodendrocyte glycoprotein antibody-positive pediatric autoimmune encephalitis without demyelination: A case series. Frontiers in Immunology. 2022;13:1050688.

107. Masuda H, Mori M, Uzawa A, et al. Elevated serum levels of bone morphogenetic protein-9 are associated with better outcome in AQP4-IgG seropositive NMOSD. Sci Rep-uk. 2023;13(1):3538.
